# Exploring disulfiram mechanisms in renal fibrosis: insights from biological data and computational approaches

**DOI:** 10.3389/fphar.2025.1480732

**Published:** 2025-03-18

**Authors:** Vishal S. Patil, Chandragouda R. Patil, Harun M. Patel, Anoop Kumar

**Affiliations:** ^1^ Department of Pharmacology, R. C. Patel Institute of Pharmaceutical Education and Research, Shirpur, India; ^2^ Department of Pharmaceutical Chemistry, R. C. Patel Institute of Pharmaceutical Education and Research, Shirpur, India; ^3^ Department of Pharmacology, Delhi Pharmaceutical Sciences and Research University (DPSRU), New Delhi, India

**Keywords:** chronic kidney disease, disulfiram, network pharmacology, molecular dynamics, renal fibrosis

## Abstract

**Background:**

Disulfiram (DSF) is an anti-alcoholic drug that has been reported to inhibit the epithelial-to-mesenchymal transition and crosslinking during fibrosis, pyroptosis, and inflammatory NF-κB and Nrf-2 signaling pathways. However, there is insufficient evidence to support the mechanisms of DSF in preventing renal fibrosis (RF). Therefore, the current study aimed to elucidate the DSF-modulated targets and pathways in renal fibrosis.

**Methods:**

The common proteins between DSF and RF were screened for protein–protein interaction, pathway enrichment, cluster, and gene ontology analysis. Molecular docking was executed for core genes using AutoDock Vina through the POAP pipeline. Molecular dynamics (MD) simulation (100 ns) was performed to infer protein–ligand stability, and conformational changes were analyzed by free energy landscape (FEL).

**Results:**

A total of 78 targets were found to be common between DSF and RF, of which *NFKB, PIK3CA/R1*, *MTOR*, *PTGS2*, and *MMP9* were the core genes. PI3K-Akt signaling followed by JAK-STAT, TNF, Ras, ErbB, p53, phospholipase D, mTOR, IL-17, NF-κB, AMPK, VEGF, and MAPK signaling pathways were modulated by DSF in RF. DSF showed a direct binding affinity with active site residues of core genes, and except for DSF with NF-κB, all other complexes, including the standard, were found to be stable during 100 ns MD simulation with minimal protein–ligand root mean squared deviation and residual fluctuations and higher compactness with broad conformational changes.

**Conclusion:**

DSF protects against renal fibrosis, and this study paves the way for experimental investigation to repurpose DSF for treating RF.

## 1 Introduction

Chronic kidney disease (CKD) is characterized by long-term structural and functional issues pertaining to the kidneys, lasting for at least 3 months and resulting in deterioration of health (Romagnani et al., 2017). The estimated worldwide prevalence of CKD is around 10%–14% of the population, and numerous people with the condition may not have symptoms until it progresses to end-stage renal fibrosis (ESRF) (Kovesdy, 2022). The main consequences of CKD include the irreversible and progressive loss of nephrons, tubular atrophy, chronic inflammatory changes, decreased regeneration capacity, renal microvascular impairment, and metabolic abnormalities, finally resulting in ESRF (Panizo et al., 2021). CKD contributes to global morbidity and mortality rates, emphasizing the demand for a variety of treatment approaches to address CKD and its development into fibrotic changes (O’Callaghan-Gordo et al., 2019). In CKD, the damage to the kidneys prompts the nearby fibroblasts and pericytes to release inflammatory substances and initiate the development of the extracellular matrix (ECM) as a means of repairing the injury. The recurrent renal injuries result in the formation of excessive ECM, which disrupts the structure of the kidneys and has a negative impact on their function, ultimately leading to renal failure (Rayego-Mateos et al., 2021).

Kidney disease affects more than 850 million people worldwide, and by 2040, it is expected to be the fifth leading cause of years of life lost (YLL) globally. The Global Burden of Disease (GBD) reports indicate that the prevalence of CKD increased by 33% worldwide between 1990 and 2017 (Francis et al., 2024). Most CKD patients live outside of middle- and high-income countries (HICs) (in India and China alone), accounting for approximately one-third of the global increase in CKD burden (prevalence and mortality) (Lameire et al., 2021).

The Indian Council of Medical Research (ICMR) has established a standardized workflow for the treatment of CKD (ICD-10-N-18.3) (Fenton and Benigni, 2014). In addition to non-pharmacological measures (diet, physical activity, lifestyle behavior, etc.), the therapy options for CKD and fibrosis include angiotensin-converting enzyme (ACE) inhibitors, angiotensin receptor blockers (ARBs), etc., and dialysis for severe cases (Fried et al., 2021). There are currently no medications that directly focus on addressing fibrosis associated with CKD, and the available drugs are for the management of cardiovascular disorders, urolithiasis, etc. Currently, only two antifibrotic drugs, pirfenidone and nintedanib, are particularly marketed for the treatment of pulmonary fibrosis (Okano et al., 2020; Hadda and Guleria, 2020). Hence, there is an urgent need for drugs for the treatment of RF; however, the discovery and development process of NCE is expensive and time-consuming. Therefore, alternate approaches like drug repurposing can be used, which take less time and money due to the known behavior of these drugs in large populations (Okano et al., 2020; Hadda and Guleria, 2020).

Renal fibrosis (RF) is a complex process driven by several key signaling pathways that contribute to the transformation of renal cells and the accumulation of ECM. The TGF-β signaling pathway is central to fibrogenesis, promoting epithelial-mesenchymal transition (EMT) and myofibroblast activation, leading to ECM deposition in response to renal injury (Zeisberg et al., 2008; Meng et al., 2015). The Wnt and Notch signaling pathways are also critical; while transient activation aids in tissue repair, sustained activation exacerbates fibrosis by inhibiting epithelial differentiation and promoting fibroblast proliferation (Edeling et al., 2016; Huang et al., 2023). Additionally, FGFR1 signaling influences cell proliferation and ECM production, linking it to fibrogenesis (Xu et al., 2022). The Hedgehog signaling pathway plays a role in regulating myofibroblast differentiation and maintaining kidney development, with dysregulation contributing to fibrosis (Edeling et al., 2016; Zhou et al., 2016). Furthermore, DPP-4 and Angptl4 are recognized as key fibrogenic molecules that promote ECM accumulation and myofibroblast activation, underscoring their significance in renal fibrotic processes (Daza-Arnedo et al., 2021; Li et al., 2024).

EMT and endothelial-to-mesenchymal transition (EndMT) are critical mechanisms in the development of RF, where renal epithelial and endothelial cells transform into myofibroblasts, contributing to the excessive accumulation of ECM and kidney scarring (He et al., 2013; Cruz-Solbes and Youker, 2017). In EMT, renal tubular epithelial cells lose their epithelial characteristics, such as E-cadherin expression, and acquire mesenchymal properties, including α-SMA, vimentin, and fibronectin expression, often triggered by factors like TGF-β1, oxidative stress, and inflammation (Singh and Mathew, 2022). This transformation leads to enhanced production of ECM components, promoting fibrosis. Similarly, EndMT involves the conversion of endothelial cells into mesenchymal-like cells, driven by signals like TGF-β1 and mechanical stress (e.g., increased blood pressure, and altered hemodynamics) (Pardali et al., 2017; Jacobs et al., 2024). These cells lose their endothelial markers, such as vascular endothelial (VE)-cadherin, and gain mesenchymal markers like α-SMA and fibroblast-specific protein 1 (FSP-1) (Zeisberg et al., 2008). Both EMT and EndMT play pivotal roles in the progression of renal fibrosis, driving the formation of myofibroblasts that fuel ECM production, inflammation, and tissue remodeling, thus offering potential targets for therapeutic intervention in kidney fibrosis (Zeisberg et al., 2008; Pardali et al., 2017; Jacobs et al., 2024).

The management of RF has been the focus of extensive research, with several therapeutic agents being evaluated for their efficacy in preclinical models. Empagliflozin, a sodium-glucose cotransporter 2 (SGLT2) inhibitor, has shown significant antifibrotic effects in diabetic mouse models by inhibiting the EMT and restoring kidney histology and function (Li J. et al., 2020). Empagliflozin has been shown to normalize levels of SIRT3, a protein involved in mitochondrial function and metabolism, which is often suppressed in diabetic conditions. This restoration helps inhibit the abnormal glycolysis associated with kidney fibrosis (Abbas et al., 2018). Studies indicate that SIRT3 knockout mice exhibit exacerbated RF when subjected to chronic AngII infusion, while SIRT3-overexpressing mice show reduced kidney injury (Li et al., 2017). Compounds like honokiol have been identified as activators of SIRT3, demonstrating protective effects against RF (Quan et al., 2020; Wei et al., 2023). Linagliptin (Lina), a DPP-4 inhibitor, exhibits renoprotective effects (Kanasaki, 2018; Nady et al., 2024) beyond diabetes. In a rat model, Lina reduced renal dysfunction, ECM deposition, and fibrosis markers, including TGF-β1, Smad4, p-ERK1/2, and p-P38 MAPK. Additionally, it inhibited EMT by suppressing vimentin and α-SMA while upregulating E-cadherin. Lina also reduced hypoxia-related proteins, downregulating Snail and Twist. Targeting the ROCK2-TGF-β1-EMT and ROCK2-Nrf2 pathways may offer a novel therapeutic strategy for CKD (You et al., 2020). Ac-SDKP, a naturally occurring peptide, has shown potential to protect against RF by inhibiting fibroblast proliferation and collagen deposition. A study investigating RF in a unilateral ureteral obstruction (UUO) model using wild-type and PAI-1 knockout mice assessed the effects of thymosin β4 (Tβ4) and its degradation product N-acetyl-seryl-aspartyl-lysyl-proline (Ac-SDKP). Ac-SDKP consistently reduced fibrosis, while Tβ4 exhibited profibrotic effects when combined with a prolyl oligopeptidase inhibitor but promoted repair in late-stage fibrosis (Zuo et al., 2013; Nitta et al., 2016).

Disulfiram (DSF), a well-known drug used to treat chronic alcohol dependence, has been identified as a nephroprotective medication that decreases epithelial–mesenchymal transition (EMT) and prevents the accumulation of collagen by regulating many molecular pathways implicated in renal fibrosis (Liu et al., 2021). It is also reported to possess anti-inflammatory (Kanai et al., 2010) and anti-cancer effects (Ekinci et al., 2019; Jiao et al., 2016). Owing to the broad therapeutic potential of DSF, repurposing of DSF has garnered interest recently. Clinical trials on the use of DSF for various diseases, such as COVID-19 (Fillmore et al., 2021), human immunodeficiency virus infection, and refractory multiple myeloma, have been conducted or are ongoing (Elliott et al., 2015; Weiser et al., 2021). DSF, as a novel proteasome inhibitor, is studied to inhibit nuclear translocation and DNA binding activity of NF-κB in certain cancers (Zha et al., 2014). DSF reversed the TGF-β-induced EMT program in a dose-dependent manner in the MCF-7 cell line and the mice model (Han et al., 2015). Combined with copper-inhibited transforming growth factor β1, TGF-β1 induced α-smooth muscle actin (α-SMA) expression and suppressed fibroblast activation (Li Y. et al., 2020). It decreased the expression of GSDMD and downregulated the level of α-SMA in renal tissues, inhibiting pyroptosis and improving renal fibrosis in rats (Zhang et al., 2021). Based on these studies, we intended to extensively explore the overall possible mechanisms of DSF that corroborate its repurposability in the management of renal failure.

In this study, we predicted the overall possible interactions between DSF (ligand) and its modulated genes through target identification, gene set enrichment analysis, and network analysis. As a result, we identified *NFKB* (p65/p50), *PIK3R1/PIK3CA* (p85 regulatory subunit/p110α catalytic subunit), *MTOR, PTGS2*, and *MMP9* as core nodes in RF. We further aimed to evaluate the binding affinity of DSF with core protein targets using molecular docking, assess complex stability by molecular dynamics simulation, and explore conformational changes using principal component analysis and free energy landscape.

## 2 Materials and methods

### 2.1 DSF and RF targets

Canonical SMILES of DSF was retrieved from the PubChem (Kim et al., 2019) database (https://pubchem.ncbi.nlm.nih.gov/; CID: 3,117) and queried for the target prediction in BindingDB (Liu et al., 2007) with probability ≥70%, SuperPred (p ≥ 50%) (Gallo et al., 2022), STITCH (Kuhn et al., 2007), probable mRNA-based gene expression using DIGEP-Pred (Lagunin et al., 2013) servers expressed genes (up and downregulated) with pharmacological activity (Pa) greater than pharmacological inactivity (Pi)), and from the peer review of literature. Similarly, disease genes were retrieved from the therapeutic target database (TTD) (Chen et al., 2002) and the GeneCards (Stelzer et al., 2016) database using the keywords “chronic kidney disease,” “end-stage renal failure,” and “kidney fibrosis.” The relevance score was set to ≥20 to get an optimum number of genes for pathway enrichment analysis. The commonly shared genes between DSF and RF were obtained and subjected to pathway enrichment analysis.

### 2.2 DSF-modulated RF pathways

The common genes were queried in STRING (Mering et al., 2003) *ver* 12.0 to visualize and construct the protein–protein interaction (PPI) network for the same. The high-confidence target protein interaction data was set with a score level greater than 0.4. Within the PPIs, k-means clustering was applied to find a defined number of clusters based on their centroids. Centroid value is the most complex node centrality index and considers couples of nodes (i, j). The centroid value of a node “i” is the number of nodes with the minimum shortest path that are closer to “i” than “j.” The highest centroid node has the highest number of neighbors separated by the shortest path to it (Lu et al., 2016). Afterward, the pathways modulated by a set of genes with respect to *Homo sapiens* were obtained from the Kyoto Encyclopedia of Genes and Genomes (KEGG; https://www.genome.jp/kegg/pathway.html) (Kanehisa et al., 2017). Pathways with p-values less than 0.05 were considered statistically significant. To control for the potential false positives arising from multiple hypothesis testing, we applied a false discovery rate (FDR) correction. This approach ensures that the findings reported as significant are more likely to represent true discoveries rather than being the result of random chance. The pathways related to the RF were identified from published literature. The clusters, biological processes, molecular function, and cellular components were identified through gene ontology (GO) analysis.

### 2.3 Network analysis

The combined network of DSF, regulated proteins, and modulated pathways was constructed using Cytoscape ver. 3.9.1 (https://cytoscape.org/) (Shannon et al., 2003). The duplicate nodes/edges were removed during the network construction to avoid false hits. The whole network was then treated as directed before analysis. The final network was analyzed based on the edge count topological parameter. In addition, the node size and color were set as “low values to small size” and “low values to blue colors (high values to green),” respectively (Patil et al., 2023a; Khanal et al., 2023).

### 2.4 Molecular docking to assess the binding affinity of DSF with macromolecules

#### 2.4.1 Macromolecule preparation

Based on the network analysis, NFκB (p65/p50) PIK3R1/PIK3CA (p85/p110α), mTOR, PTGS2, and MMP9 were chosen for molecular docking. The X-ray crystallographic structures of the protein molecules were retrieved from the RCSB PDB database with PDB IDs 1NFI (Jacobs and Harrison, 1998), 4JPS (Furet et al., 2013), 4JSX (Yang et al., 2013), 5IKQ (Orlando and Malkowski, 2016), and 1GKC (Rowsell et al., 2002), respectively. The missing amino acid residues in the PIK3R1/PIK3CA (PDB ID: 4JPS) were filled through template-based modeling via the SwissModel server (Schwede et al., 2003), validated using the SAVES v6.1 server (https://saves.mbi.ucla.edu/) by considering Ramachandran plot analysis, VERIFY3D, and ERRAT quality checks. The amino acids were renumbered using PDB-Editor (Lee and Kim, 2009).

#### 2.4.2 Identification of ligand binding sites

The active site and functional domain information of the protein residues were obtained through the CASTp (Tian et al., 2018) and P2Rank (Krivák and Hoksza, 2018) servers.

#### 2.4.3 Ligand preparation

The structure of DSF (CID: 3117) and standard compounds from the co-crystal structures, except for NFκB, were obtained from the PDB. The standard compound for NF-κB was sulfasalazine (CID: 5339; targeting p65/p50) retrieved from the PubChem database. The standard compound for PIK3R1/PIK3CA was Alpelisib (CID: 56649450); for mTOR: Torin-2 (CID: 51358113); for PTGS2: meclofenamic acid (CID: 4037); and for MMP9: N∼2∼-[(2R)-2-{[formyl (hydroxy)amino]methyl}-4-methylpentanoyl]-N, 3-dimethyl-L-valinamide (NFH; CID: 5287851). The structures of standards were used from the PDB file. The compound energies were minimized through the POAP pipeline (Samdani and Vetrivel, 2018) ligand preparation script “*POAP_lig.bash*” by applying the *mmff94* force field via the conjugate gradient method.

#### 2.4.4 Ligand–protein docking

Molecular docking of DSF and standard compounds with the prioritized protein targets was performed using AutoDock Vina (Trott and Olson, 2010) through the POAP pipeline employing the ‘POAP_vs.bash’ script (Samdani and Vetrivel, 2018; Patil et al., 2023b). The system exhaustiveness was set to the default value of 8. The docking procedure generated nine docked conformations, from which the best ligand conformation was selected based on a combination of the lowest docking energy (docking, kcal/mol) and the lowest root-mean-square deviation (RMSD). The resulting complex with the lowest RMSD and binding energy was then visualized for intermolecular interactions using Discovery Studio Visualizer version 2019. The docking validation was performed by analyzing the overlay similarity between the docked conformation of the standard drug and its experimentally determined X-ray PDB conformation. The root-mean-square deviation (RMSD) was calculated using the PyMOL tool (DeLano, 2002).

### 2.5 Molecular dynamics

The protein complexes formed with DSF and the standard drug underwent all-atom explicit molecular dynamics (MD) simulations for 100 ns in an explicit solvent using the *ff99SBildn* force field in *xleap* tool of Amber antechamber (Wang et al., 2001) with GROMACS software (Lindahl et al., 2001). The docked complexes were solvated in a cubic box using the TIP3P water model, with periodic boundary conditions applied at a distance of 10 Å from all protein edges. To neutralize the system, Na^+^/Cl^−^ counter ions were added wherever necessary. Energy minimization was performed using the steepest descent method followed by the conjugate gradient method to achieve near-global minimum energy conformations. The systems were equilibrated using canonical (NVT) and isobaric (NPT) ensembles for 1 ns each. During NVT equilibration, a modified Berendsen thermostat maintained a constant volume and temperature at 300 K, while during NPT equilibration, the Parrinello–Rahman barostat maintained a constant pressure of 1 bar. The particle mesh Ewald (PME) method, with a cutoff of 1 nm, was used to compute coulomb, van der Waals, and long-range electrostatic interactions. The LINear Constraint Solver (LINCS) algorithm was employed to constrain bond lengths. The complexes were then subjected to a 100 ns production run, with coordinates recorded every 2 fs. The resulting trajectories were analyzed using built-in GROMACS utilities and additional software tools as needed. The stability of the docked complexes was assessed by examining the RMSD of backbone and complex atoms, root-mean-square fluctuation (RMSF) of C-alpha atoms, radius of gyration (Rg) of ligand and protein atoms, solvent-accessible surface area (SASA) of protein, and hydrogen bonds (H-bonds) between ligand and protein.

### 2.6 MMPBSA and decomposition energy

The MMPBSA approach was employed to estimate binding affinity, utilizing 50 frames for energy calculation. In typical scenarios, where comparing states with similar entropies, such as two ligands binding to the same protein, entropy contributions were excluded. The relative binding energy and its contribution to single residues were calculated using the g_mmpbsa module. Equations specific to the MMPBSA methods for calculating binding energy (Kumari et al., 2014; Shivankar et al., 2024) are as follows:

$${\Delta G}_{\text{bind}}=G_{\text{complex}}-\left(G_{\text{receptor}}+G_{\text{ligand}}\;\right),$$

=ΔH – TΔS,

$$={\Delta E}_{\text{MM}}+{\Delta S}_{\text{solv}}-T\Delta S,$$

$${\Delta G}_{\text{MM}}={\Delta E}_{\text{bonded}}+{\Delta E}_{\text{ele}}+{\Delta E}_{\text{vdw}},$$

$${\Delta G}_{\text{solv}}={\Delta G}_{\text{polar}}+{\Delta G}_{\text{nonpolar},}$$

$${\Delta G}_{nonpolar}=\gamma \times \Delta \text{SASA}+\beta ,$$

where the ΔG_bind_ is the total binding free energy. It represents the free energy difference between the bound state (G_complex_) and the free state (G_receptor_ + G_ligand_) and can be represented by the sum of the enthalpy part (ΔH) and the entropy part (−TΔS). The enthalpy part can be further broken into molecular mechanical energy (ΔE_MM_) and solvation-free energy (ΔG_solv_). The ΔE_MM_ term undertakes the intra-molecular (ΔE_bonded_), the electrostatic (ΔE_ele_), and the van der Waals (ΔE_vdW_) energies. The ΔG_solv_ term contains both polar (ΔG_polar_) and nonpolar (ΔG_nonpolar_) contributions, where the polar contributions are accounted for by the Poisson Boltzmann (PB) model and the nonpolar contributions are assumed to be proportional to the solvent-accessible surface area (SASA). γ links the extent of this surface area to the free energy. γ × ΔSASA models the energetic cost of solvent interactions with the hydrophobic regions of the molecule. The larger the exposed surface area, the greater the solvation-free energy. β is an additive constant that accounts for contributions to the nonpolar solvation-free energy that is not directly dependent on SASA.

### 2.7 Principal component analysis (PCA)-based free energy landscape (FEL) of protein–ligand complexes

Principal component analysis (PCA) was employed to analyze the diverse molecular motions of amino acids using molecular dynamics trajectories (Bhandare and Ramaswamy, 2018; Amadei et al., 1996). This method involved aligning the trajectory to a reference structure using “least square fit” to eliminate translational and rotational mobility. A covariance matrix was then generated through a linear transformation of Cartesian coordinate space. This matrix was diagonalized to yield eigenvectors, each representing a direction of molecular motion and associated eigenvalues, indicating the energy contribution of each motion component. The trajectory was projected onto these eigenvectors to illustrate time-dependent motions, revealing specific vibrational modes. The temporal average of these projections quantified the contribution of atomic vibrations to coordinated motions. Finally, the conformational and transitional states of complexes were visualized and analyzed over the trajectory by extracting different conformations using the FEL graph. GROMACS utilities “*g_covar*,” and covariance matrices were utilized to compute and diagonalize eigenvectors and eigenvalues from the trajectory data. Additionally, the “*g_anaeig*” program facilitated the analysis and visualization of these eigenvectors (Bhandare and Ramaswamy, 2018; Amadei et al., 1996).

## 3 Results

### 3.1 DSF and RF targets

**FIGURE 1:**
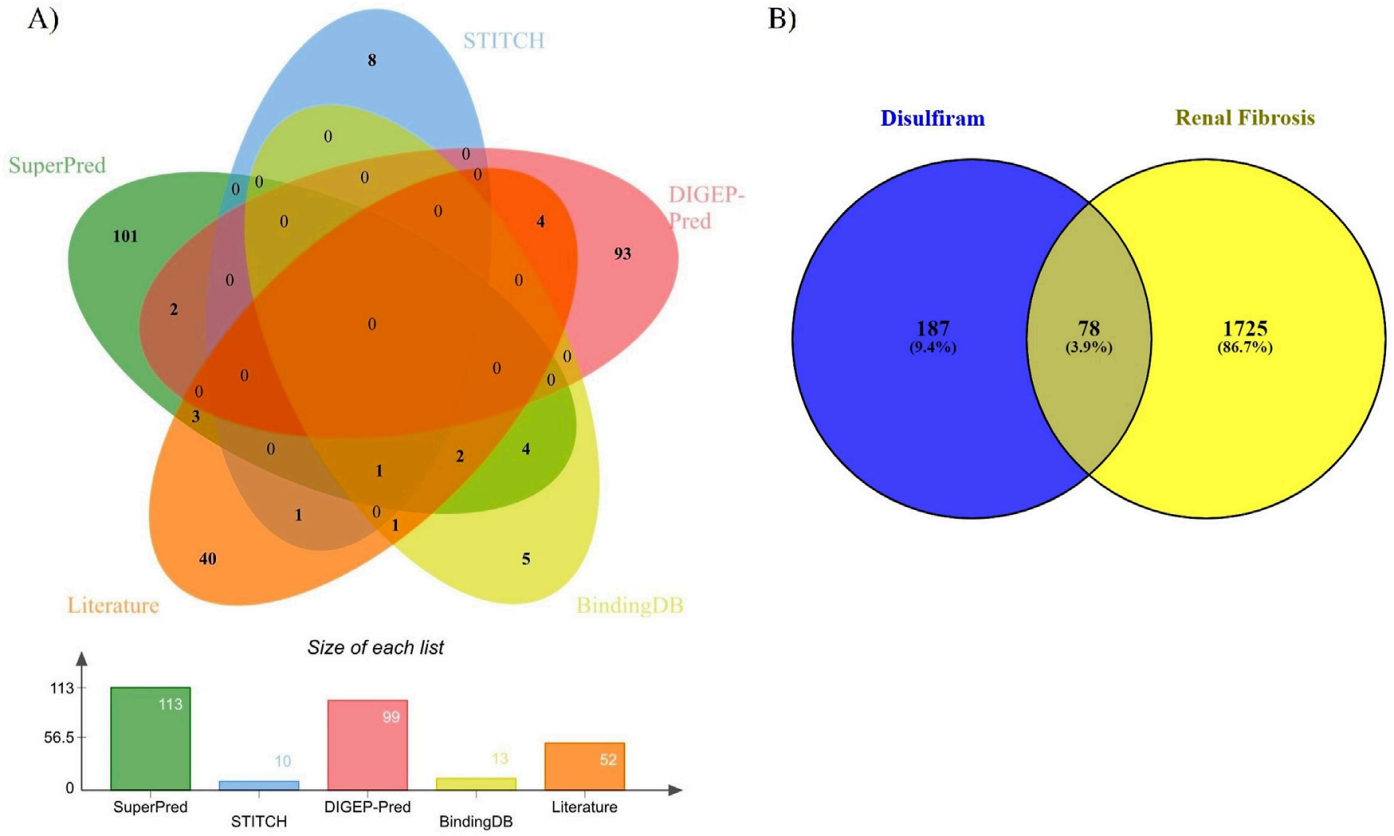
**(A)** Common genes of DSF from SuperPred, STITCH, DIGEP-Pred, BindingDB, and the literature. **(B)** Common genes between DSF and renal fibrosis.

DSF was predicted to target 113 protein molecules in SuperPred, 10 in STITCH, 99 in DIGEP-Pred (51 were downregulated and 48 were upregulated), 13 in BindingDB, and 52 from published literature. Overall, DSF was identified to target 265 protein molecules after removing duplicates (22). Renal fibrosis-associated genes were retrieved from the gene cards database (relevance score ≥20) using different keywords: kidney fibrosis (1), chronic kidney diseases (1707), aZend-stage renal failure (719), and nine genes were retrieved from the TTD database. Overall, 1803 protein targets were identified for RF. The common targets between DSF and RF were analyzed, and 78 were identified (Figure 1). The list of DSF-modulated targets is provided in Supplementary Table 1, and a list of RF targets is provided in Supplementary Table 2.

### 3.2 DSF-targeted protein interactions

**FIGURE 2:**
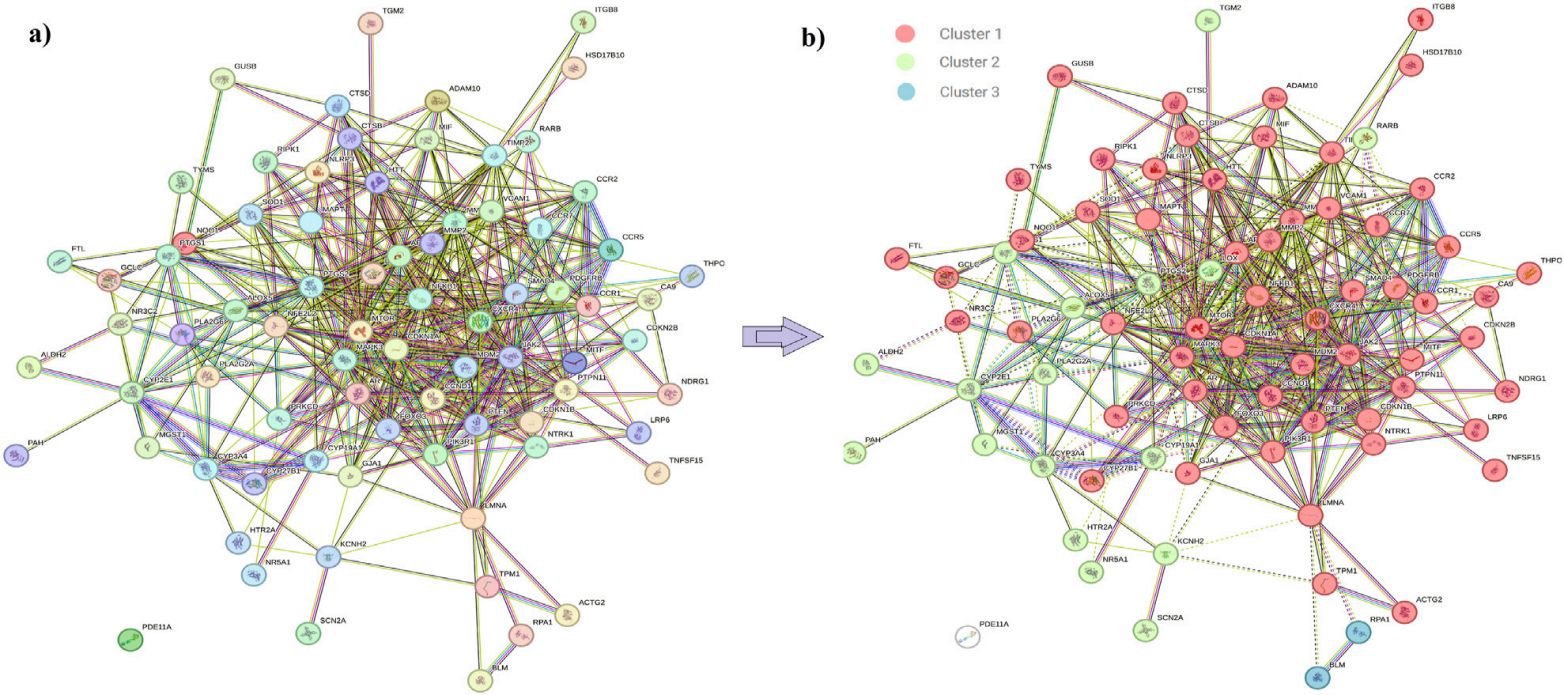
**(A)** Protein–protein interaction of the DSF-triggered protein. Node color; 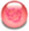 colored nodes: query proteins and first shell of interactions, 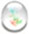 white nodes: second shell of interactors, Node content; 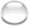
empty nodes: proteins of unknown 3D structure, 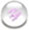 filled nodes: some 3D structure is known or predicted, Known Interactions; 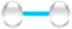 from curated databases,
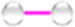
experimentally determined, Predicted Interactions; 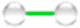
gene neighborhood, 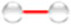 gene fusions, 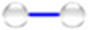 gene co-occurrence and others; 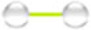
text mining, 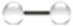
co-expression, 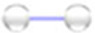 protein homology. **(B)** k-means cluster analysis based on their centroids. Centroid value is the most complex node centrality index and considers couples of nodes (i, j). The centroid value of a node i is the number of nodes with the minimum shortest path that are closer to “i” than “j.” The highest centroid node has the highest number of neighbors separated by the shortest path to it.

In order to enhance visualization and understand the mechanism of the targets, it is important to study the PPI of the target genes. The interactions among the target proteins are depicted in Figure 2, which comprises 78 nodes and 500 edges; each edge represents PPIs. The other parameter is the average node degree, which is valued at 12.8, and the local clustering co-efficient: 0.551 corresponds to the number of targets that are connected to the network. Within the PPIs, k-means clustering of 78 nodes, 58 nodes fell within Cluster 1, 17 in Cluster 2, and 2 in Cluster 3 (Supplementary Table 3; Figure 2), indicating genes within Cluster 1 have a potential role in the pathogenesis of RF.

### 3.3 DSF-targeted pathways involved in RF

**FIGURE 3:**
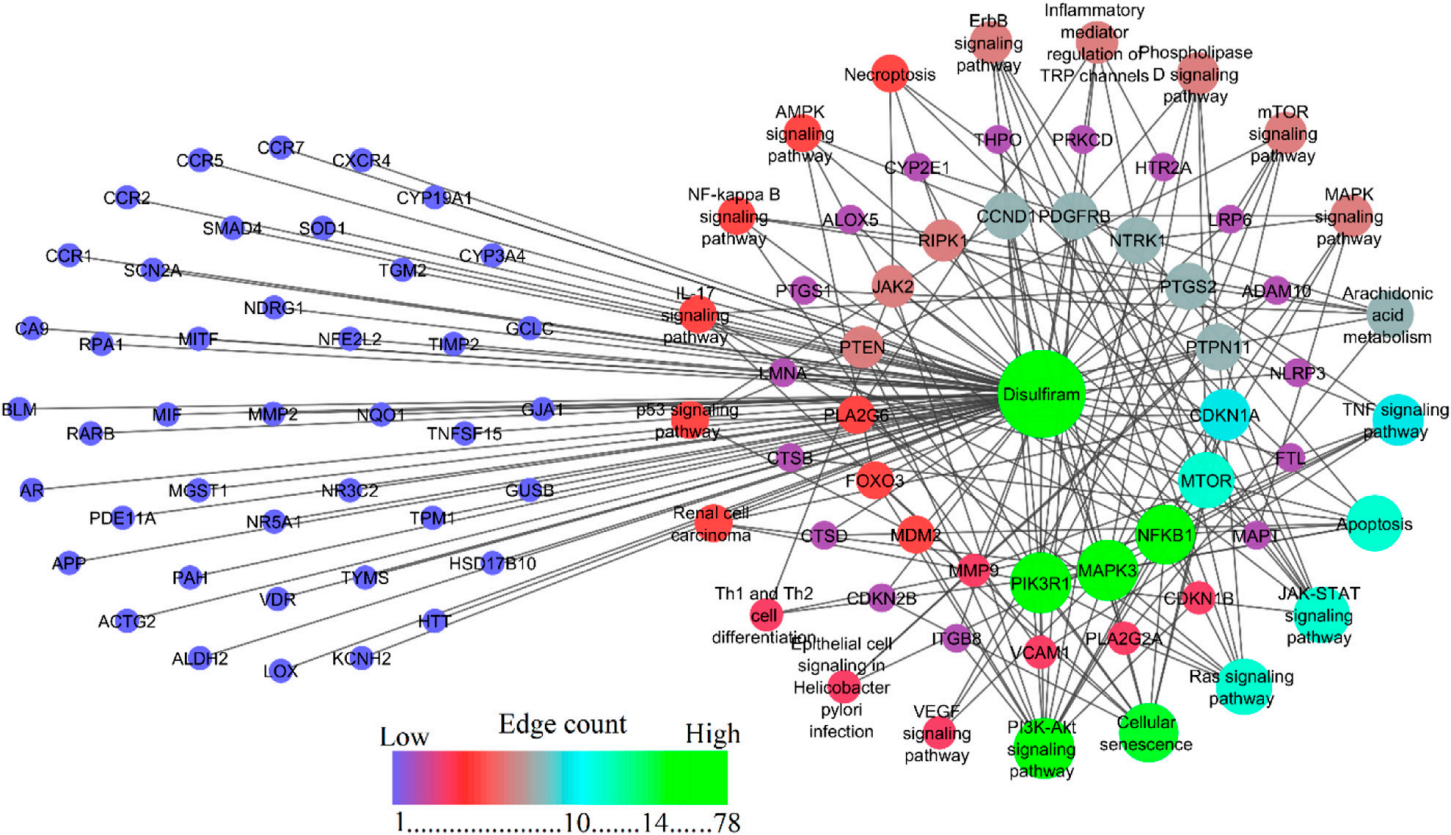
Network representation of DSF, its modulated targets, and pathways involved in RF. Nodes shown in blue are DSF-modulated targets involved in the RF but are not enriched to be involved in the molecular pathways. The provided figure represents a network map showing the interactions and pathways associated with disulfiram. The central green node labeled “disulfiram” indicates the drug around which the interactions are mapped. Edges between nodes are color-coded based on edge betweenness and edge count, with blue, green, and red representing low, medium, and high values, respectively. The color gradient from blue to red represents an increasing edge count.

To study the signaling pathway and function of the selected target genes, the data were imported to Cytoscape to construct the compound-target network. Figure 3 shows the compound-target-disease interaction network, which elucidates the mechanisms of DSF action in RF treatment. DSF was found to modulate 138 molecular pathways, of which 21 are associated with the RF (Table 1). In the pathway enrichment analysis, the PI3K-Akt signaling pathway was identified as the enriched pathway that scored a gene count of 14 and the lowest FDR of 5.70 × 10⁻⁹. The JAK-STAT, TNF, Ras, ErbB, p53, phospholipase D, mTOR, IL-17, NF-κB, AMPK, VEGF, and MAPK signaling pathways were also modulated by the 78 targets. Furthermore, cellular senescence, apoptosis, inflammatory mediator regulation of TRP channels, renal cell carcinoma, arachidonic acid metabolism, epithelial cell signaling in *Helicobacter pylori* infection, necroptosis, and Th1 and Th2 cell differentiation mechanisms were also modulated by DSF targets. *PIK3R1, MAPK3*, NFκB1, mTOR, etc., were identified as hub genes within the network and are represented through the higher node size. Alongside, therapeutic targets of RF, mainly *PTGS2, MMP9, MMP2, SMAD4*, etc., were also modulated by DSF. This fact inferred that the DSF might influence these targets synergistically; it has therapeutic effects on other diseases and disorders in addition to RF. The details of the network between DSF, its targets, and modulated pathways are represented in Figure 3. Supplementary Table 4 gives overall pathways modulated by the 78 targets.

**TABLE 1:** Pathways involved in RF modulated by DSF.

KEGG ID	Pathway description	Gene count	False discovery rate	Set of genes within the network
hsa04151	PI3K-Akt signaling pathway	14	5.70 × 10⁻^9^	*ITGB8, NFKB1, CCND1, CDKN1B, MDM2, PDGFRB, MAPK3, FOXO3, MTOR, PTEN, JAK2, CDKN1A, PIK3R1,* and *NTRK1*
hsa04218	Cellular senescence	10	1.70 × 10⁻^6^	*NFKB1, CCND1, MDM2, MAPK3, CDKN2B, FOXO3, MTOR, PTEN, CDKN1A,* and *PIK3R1*
hsa04210	Apoptosis	8	1.01 × 10⁻^6^	*NFKB1, CTSD, RIPK1, MAPK3, CTSB, LMNA, PIK3R1,* and *NTRK1*
hsa00590	Arachidonic acid metabolism	6	2.61 × 10⁻^6^	*PLA2G6, PTGS1, PTGS2, ALOX5, PLA2G2A,* and *CYP2E1*
hsa04630	JAK-STAT signaling pathway	8	3.11 × 10⁻^6^	*CCND1, PDGFRB, MTOR, JAK2, CDKN1A, PIK3R1, PTPN11,* and *THPO*
hsa04668	TNF signaling pathway	7	4.26 × 10⁻^6^	*NFKB1, RIPK1, MAPK3, VCAM1, PTGS2, MMP9,* and *PIK3R1*
hsa04014	Ras signaling pathway	8	2.90 × 10⁻^5^	*NFKB1, PDGFRB, MAPK3, PLA2G6, PLA2G2A, PIK3R1, NTRK1,* and *PTPN11*
hsa04012	ErbB signaling pathway	5	1.30 × 10⁻^4^	*CDKN1B, MAPK3, MTOR, CDKN1A,* and *PIK3R1*
hsa04750	Inflammatory mediator regulation of TRP channels	5	2.30 × 10⁻^4^	*PRKCD, PLA2G6, PIK3R1, NTRK1,* and *HTR2A*
hsa05211	Renal cell carcinoma	4	7.40 × 10⁻^4^	*MAPK3, CDKN1A, PIK3R1,* and *PTPN11*
hsa04115	p53 signaling pathway	4	1.00 × 10⁻^3^	*CCND1, MDM2, PTEN,* and *CDKN1A*
hsa04072	Phospholipase D signaling pathway	5	1.40 × 10⁻^3^	*PDGFRB, MAPK3, MTOR, PIK3R1,* and *PTPN11*
hsa04150	mTOR signaling pathway	5	1.50 × 10⁻^3^	*LRP6, MAPK3, MTOR, PTEN,* and *PIK3R1*
hsa04657	IL-17 signaling pathway	4	2.10 × 10⁻^3^	*NFKB1, MAPK3, PTGS2,* and *MMP9*
hsa04064	NF-kappa B signaling pathway	4	2.90 × 10⁻^3^	*NFKB1, RIPK1, VCAM1,* and *PTGS2*
hsa04152	AMPK signaling pathway	4	5.10 × 10⁻^3^	*CCND1, FOXO3, MTOR,* and *PIK3R1*
hsa04370	VEGF signaling pathway	3	5.50 × 10⁻^3^	*MAPK3, PTGS2,* and *PIK3R1*
hsa05120	Epithelial cell signaling in *Helicobacter pylori* infection	3	7.80 × 10⁻^3^	*NFKB1, ADAM10,* and *PTPN11*
hsa04217	Necroptosis	4	9.10 × 10⁻^3^	*RIPK1, NLRP3, FTL,* and *JAK2*
hsa04658	Th1 and Th2 cell differentiation	3	1.46 × 10⁻^2^	*NFKB1, MAPK3,* and *JAK2*
hsa04010	MAPK signaling pathway	5	1.63 × 10⁻^2^	*NFKB1, PDGFRB, MAPK3, MAPT,* and *NTRK1*

### 3.4 GO analysis of DSF-targeted genes

#### 3.4.1 Molecular functions

**FIGURE 4:**
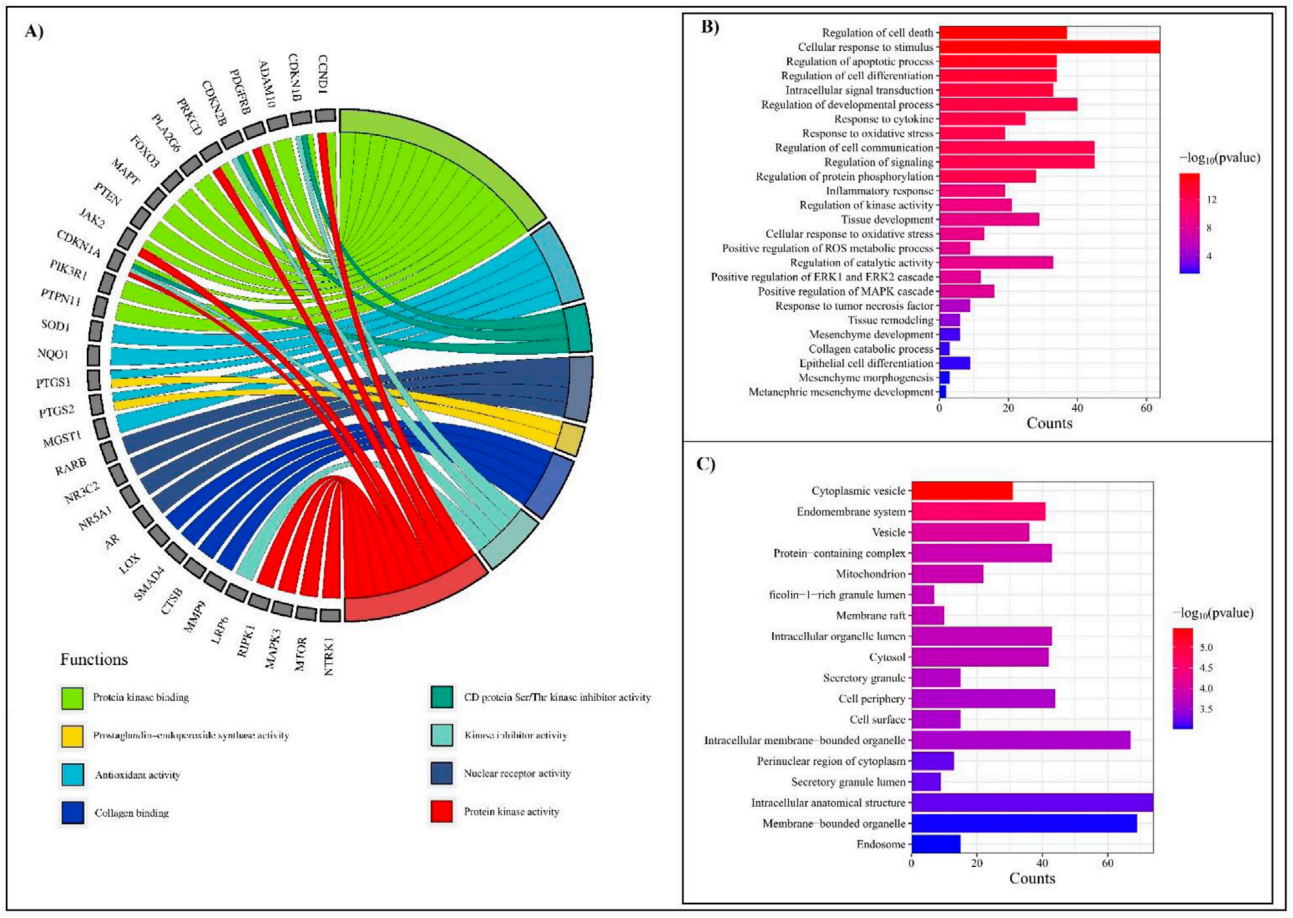
GO analysis depicting molecular functions, biological processes, and cellular components associated with RF. **(A)** Chord diagram representing molecular functions with the lowest FDR scores, highlighting functional interactions. **(B)** Bar chart showing biological processes associated with RF, ranked by significance (−log10 (p-value)). The most enriched processes include regulation of cell death, cellular response to stimuli, and inflammatory pathways. The color gradient represents the significance level, with red indicating the highest and blue indicating the lowest p-values. **(C)** Top cellular components from the GO list. Key enriched components include cytoplasmic vesicles, mitochondria, and intracellular membrane-bounded organelles. The bar length represents the frequency of occurrences, while the color gradient corresponds to −log10 (p-value).

A total of 47 different molecular function GO terms were identified via the PPI with protein catalytic activity. GO:0003824 had the lowest false discovery rate, that is, 9.84 × 10⁻^6^, to trigger 46 genes against 5,522 background genes at 0.32 strength. Herein, a total of 15 functions were associated with RF pathogenesis, viz., C-C chemokine receptor activity, C-C chemokine binding, oxidoreductase activity, protein kinase binding, antioxidant activity, cyclin-dependent protein serine/threonine kinase inhibitor activity, monooxygenase activity, nuclear receptor activity, prostaglandin-endoperoxide synthase activity, collagen binding, aromatase activity, kinase inhibitor activity, superoxide dismutase activity, and protein kinase activity (Supplementary Table 5; Figure 4A).

#### 3.4.2 Biological processes

A total of 804 biological processes were involved in the PPIs, in which programmed cell death, cellular response to stimulus, regulation of developmental process, response to oxidative stress, cytokine, tumor necrosis factor, regulation of cell communication, signaling, protein phosphorylation, inflammatory response, kinase activity, tissue development, catalytic activity, positive regulation of ROS metabolic process, ERK1 and ERK2 cascade, regulation of MAPK cascade, tissue remodeling, mesenchyme development, collagen catabolic process, epithelial cell differentiation, mesenchyme morphogenesis, and metanephric mesenchyme development were traced to be involved in the pathogenesis of RF (Supplementary Table 6; Figure 4B).

#### 3.4.3 Cellular components

GO analysis identified 58 GO terms for cellular components in which cytoplasm GO:0005737 had the lowest false discovery rate, that is, 3.54 × 10⁻^6^, to regulate 71 genes against 12,056 genes at 0.17 strength. In addition, 41 genes were triggered in endomembrane system components with an FDR score of 2.69 × 10⁻^5^ (Supplementary Table 7; Figure 4C).

#### 3.4.4 Cluster analysis

**FIGURE 5:**
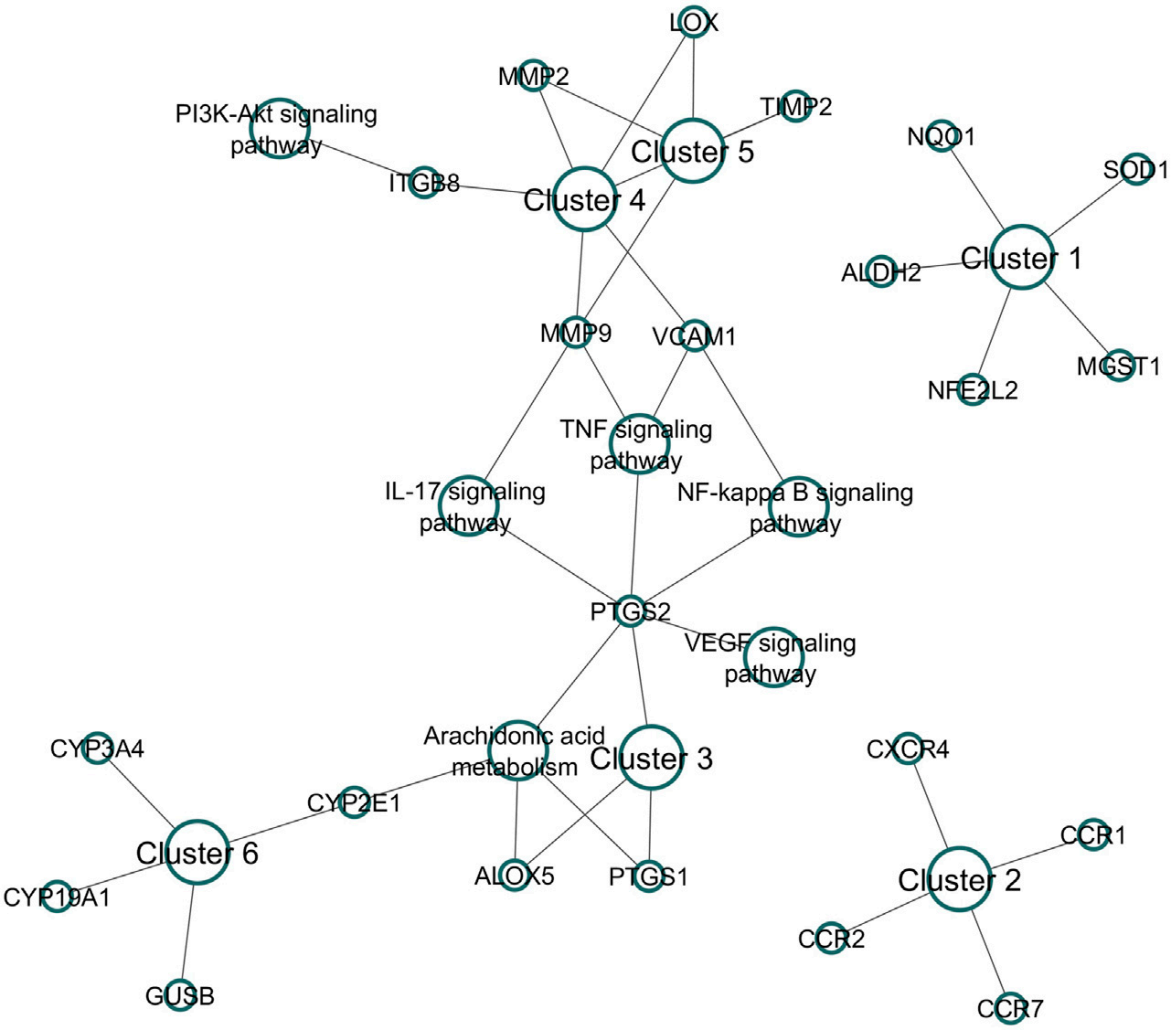
Cluster analysis for DSF-modulated targets. A total of nine clusters were enriched, of which six were associated with RF. The following were the associations of clusters in biological pathways of RF. Cluster 1: Mixed, incl. glutathione metabolism and detoxification of reactive oxygen species; Cluster 2: chemokine receptors bind chemokines; Cluster 3: prostaglandin metabolism and leukotriene receptor activity; Cluster 4: extracellular matrix organization; Cluster 5: elastic fiber formation and matrix metalloproteinases; and Cluster 6: steroid hormone biosynthesis and arachidonic acid monooxygenase activity.

Nine clusters were generated for 78 genes (Supplementary Table 8). Among them, six clusters were associated with RF (Figure 5). Cluster 1 contains six genes (*CCND1, CDKN1B, MDM2, CDKN2B, CDKN1A*), is identified as mixed for glutathione metabolism and detoxification of ROS, and scored the lowest FDR of 6.3 × 10⁻³. Cluster 2 contains four genes (*CCR7, CCR2, CCR1, CXCR4*) and is identified as mixed for chemokine receptors binding chemokines, and scored the lowest FDR of 1.34 × 10⁻^2^. Cluster 3 includes *PTGS1, PTGS2*, and *ALOX5* involved in prostaglandin metabolism and leukotriene receptor activity, whereas Cluster 4 (*MMP9, MMP2, ITGB8, LOX, TIMP2,* and *VCAM1*) and Cluster 5 (*MMP9, MMP2, LOX, TIMP2*) were found to trigger extracellular matrix organization, elastic fiber formation, and matrix metalloproteinases activity. Likewise, Cluster 6 contains four genes (*GUSB, CYP19A1, CYP2E1, CYP3A4*) that were found to be involved in the steroid hormone biosynthesis and arachidonic acid monooxygenase activity.

### 3.5 Molecular docking

Among the selected targets, PIK3R1/PIK3CA (PDB ID: 4JPS) contained missing residues and were remodeled using the Swiss Model. The model passed the Varify3D with an 82.33% score and showed an ERRAT2 quality factor of 95.45% (Supplementary Figures S1A–E). Furthermore, the Ramachandran plot revealed around 98.9% of the residues in the most favored and additionally allowed region, indicating a good quality model. Supplementary Table 9 provides active site residues and the grid box information of each target. In molecular docking, all targets except NFKB were validated by redocking with their native ligands. The redocking results confirmed that the ligands remained within the active site, and their interactions closely matched those observed in the co-crystal structures (Table 2). From the docking, nine conformations were generated for each ligand, and conformations with the lowest docking energy and zero RMSD were chosen (Supplementary Table 10). DSF scored the lowest energy with PTGS2 (−5.0 kcal/mol), followed by PIK3 (−4.6 kcal/mol) and MMP9 (−4.5 kcal/mol). DSF showed docking scores of −4.2 and −3.5 kcal/mol with mTOR and NFKB, respectively. With PTGS2, DSF formed 13 hydrophobic bonds with Val116 (2), Tyr355, Leu359, Leu531, Val349 (2), Tyr348, Tyr385 (2), Trp387, Phe381, and Leu384, whereas meclofenamic acid formed two H-bonds with Ser530 and Tyr385 and 11 hydrophobic bonds with Leu531 (2), Val341 (2), Ala527 (2), Val116, Val523 (2), and Leu352 (2). With PIK3, DSF formed 11 hydrophobic bonds with Pro778, Lys802, Met772, Ile800, Trp780, Phe930, Val850, Ile932, Val851, Glu849, and Tyr836, whereas Alpelisib formed five H-bonds with Val851 (2), Ser854, and Gln859 (2) and 11 hydrophobic bonds with His855, Val851 (4), Ile932, Met922, Val850, Tyr836 (2), and Met772. Likewise, with MMP9, DSF formed one H-bond with Ala189, 11 hydrophobic bonds with His411 (3), His405, Tyr423, Leu188 (2), Val398, His401, His190, and Phe110. NFH formed five H-bonds with Glu402, Leu188, Tyr423, Gly186, and Pro421 and five hydrophobic bonds with Leu188, Tyr423, Leu187, Val398, and His401. DSF formed very poor interactions with NFKB, that is, one H-bond with Ile224 and one hydrophobic bond with Lys221, whereas sulfasalazine formed four H-bonds with Ala243, His245, Gln220, and Gln247, and six hydrophobic bonds with Val254, Arg255, Leu272, His245 (2), and Lys221. While in complex with MTOR, DSF formed 10 hydrophobic bonds with Leu2185 (2), Trp2239 (2), Val2240, Met2345, Ile2237, Pro2169 (2), and Ile2163, whereas Torin-2 formed one H-bond with Glu2190 and 14 hydrophobic bonds with Ile2237, Ile2356 (2), Tyr2225, Val2240 (2), Met2345 (2), Trp2239 (2), Leu2185 (2), Pro2169, and Ile2163. The docking scores, H-bonds, hydrophobic interactions, and interactions with active site residues of DSF and standard molecules with their respective targets are provided in Table 2. Supplementary Figures S2–S6 represent the 2D and 3D representations of the interaction of DSF and standard molecules with their respective targets.

**TABLE 2:** Docking score and intermolecular interaction of DSF and standard molecules with the prioritized targets involved in RF.

Target name (PDB ID)	Compound name (PubChem ID)	Docking score (kcal/mol)	RMSD (Å)	HBI	Non-HBI (hydrophobic interactions)	N	N′	N″
NFKB (p50/p65-RelA) (1NFI)	DSF	−3.5	--	Ile224	Lys221	1	1	2
	Sulfasalazine	−7.7		Ala243, His245, Gln220, and Gln247	Val254, Arg255, Leu272, His245 (2), and Lys221	4	6	1
PIK3R1/PIK3CA (4JPS)	DSF	−4.6		Nil	Pro778, Lys802, Met772, Ile800, Trp780, Phe930, Val850, Ile932, Val851, Glu849, and Tyr836	0	11	11
	Alpelisib (redock)	−10.2	0.498	Val851 (2), Ser854, and Gln859 (2)	His855, Val851 (4), Ile932, Met922, Val850, Tyr836 (2), and Met772	5	11	15
	Alpelisib (from PDB)	--		Val851 (2), Ser854, and Gln859 (2)	His855, Val851 (2), Ile932 (3), Met922, Val850, Tyr836 (2), Met772, Lys802, Ile800, and Ile848 (2)	5	15	19
MTOR (4JSX)	DSF	−4.2		Nil	Leu2185 (2), Trp2239 (2), Val2240, Met2345, Ile2237, Pro2169 (2), and Ile2163	0	10	10
	Torin-2 (redock)	−11.2	0.657	Glu2190	Ile2237, Ile2356 (2), Tyr2225, Val2240 (2), Met2345 (2), Trp2239 (2), Leu2185 (2), Pro2169, and Ile2163	1	14	12
	Torin-2 (from PDB)	--		Val2240	Ile2237, Ile2356, Val2240 (2), Met2345 (3), Trp2239 (2), Leu2185, Pro2169, Ile2163, and Cys2243	1	13	14
PTGS2 (5IKQ)	DSF	−5.0		Nil	Val116 (2), Tyr355, Leu359, Leu531, Val349 (2), Tyr348, Tyr385 (2), Trp387, Phe381, and Leu384	0	13	13
	Meclofenamic acid (redock)	−9.1	0.625	Ser530, Tyr385	Leu531 (2), Val341 (2), Ala527 (2), Val116, Val523 (2), and Leu352 (2)	2	11	11
	Meclofenamic acid (from PDB)	--		Ser530, Tyr385	Leu531, Ala527 (3), Val116, Val523, Val349	2	7	9
MMP9 (1GKC)	DSF	−4.5		Ala189	His411 (3), His405, Tyr423, Leu188 (2), Val398, His401, His190, and Phe110	1	11	12
	NFH (redock)	−6.7	1.336	Glu402, Leu188, Tyr423, Gly186, Pro421	Leu188, Tyr423, Leu187, Val398, and His401	5	5	10
	NFH (from PDB)	--		Leu188, Tyr423, Gly186	Leu188 (2), Gly186, Tyr393, and Ala189	3	5	7

BE: binding energy; HBI: conventional hydrogen bond interaction; Non-HBI: van der Waals, Pi–alkyl, CH, Pi–cation, Pi–sigma, Pi–Pi stacked, Pi–Pi T-shaped, etc., interactions, N: Total number of H-bond interactions; N′: Total number of other (hydrophobic) interactions; N″: Total number of interactions with active site residues. The RMSD represents the deviation between the docked conformation and the experimentally determined X-ray PDB conformation.and

To validate the docking protocol, the overlays between the docked conformations and the experimental (X-ray) PDB conformations of the standard molecules were analyzed (Table 2). The RMSD values for Alpelisib (PIK3R1/PIK3CA), Torin-2 (mTOR), meclofenamic acid (PTGS2), and NFH (MMP9) were calculated as 0.498 Å, 0.657 Å, 0.625 Å, and 1.336 Å, respectively. These low RMSD values indicate the accuracy of the docking protocol in predicting binding poses closely aligned with experimental structures (Supplementary Figure S7).

### 3.6 Molecular dynamics

**FIGURE 6:**
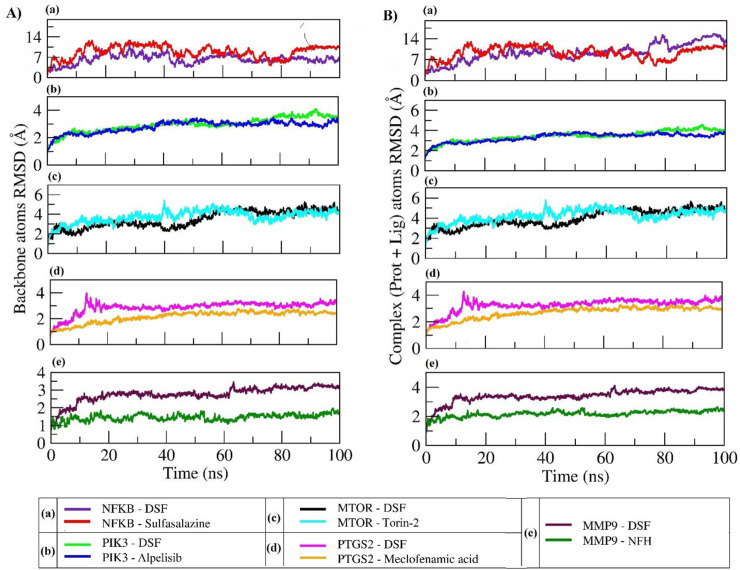
RMSD plot of backbone atoms **(A)** and complex atoms (Prot + Lig) **(B)** for the selected proteins in complex with DSF and their standard molecules during a 100 ns MD simulation.

The RMSD parameter was used to calculate the average change in atom displacement of frames over 100 ns simulation with respect to a reference frame (0 ns). All the simulated complexes except NFKB in complex with DSF exhibited stable dynamics during the 100 ns simulation. The backbone and complex atoms RMSD value for DSF and sulfasalazine with NFKB ranged between ∼10 and 14 Å with fluctuation throughout the simulation. Whereas DSF in complex with PIK3 showed very stable dynamics with a backbone and complex atoms RMSD value ranging at ∼3 Å and was comparable with the standard molecule Alpelisib. A similar trend was observed in complexes with MTOR, PTGS2, and MMP9. DSF and Torin-2 in complex with MTOR showed an average backbone and complex atoms RMSD value of ∼3.5 Å and 4.0 Å, respectively. Likewise, DSF and meclofenamic acid in complex with PTGS2 showed RMSDs ranges from 3 Å to 3.5 Å for DSF and 2 Å to 2.5 Å for meclofenamic acid, respectively. DSF and NFH in complex with MMP showed a similar trend in RMSD with average RMSDs of ∼3 Å and ∼1.5 Å for backbone and ∼3.5 Å and ∼2 Å for complex atoms, respectively (refer Figure 6).

**FIGURE 7:**
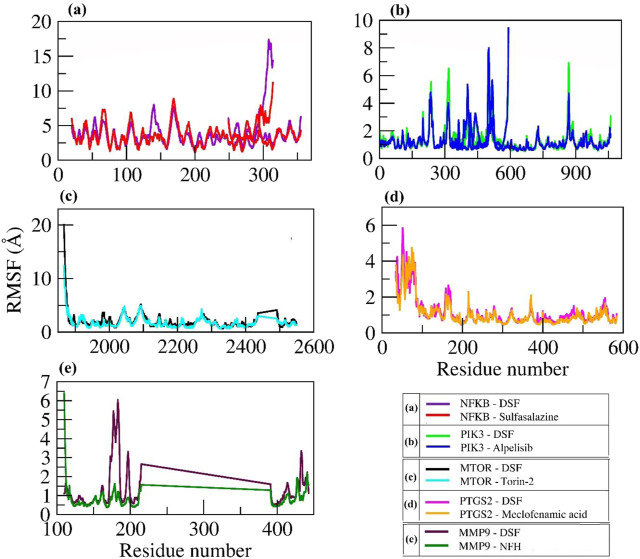
RMSF Plot of C-alpha atoms for the selected proteins in complex with DSF and their standard molecules during a 100 ns MD simulation.

Furthermore, C-α atom RMSF values were calculated to know the local changes within the protein structure. In DSF and sulfasalazine with NFκB complexes, the NFκB p50 subunit C-terminal residues showed larger fluctuations up to 17 Å and 12 Å, respectively. The residues from Arg174 to Lys195 with the longest loop showed larger fluctuation up to 8 Å, and the residues participating in the ligand contact showed minimal fluctuation. Due to a smaller number of interactions, that is, one H-bond with Ile224 and one hydrophobic bond with Lys221, DSF could not form a stable complex, and it jumped out of the binding site during the equilibration period (Movie 1). In contrast, sulfasalazine was found to be stable at the binding pocket (Movie 2), but a residual fluctuation up to 5 Å at the binding site was observed. In the DSF- and Alpelisib- PIK3 complex, residues from PIK3R1 showed larger fluctuations up to 9 Å, and the residues of PIK3CA involved in ligand binding (Met772, Pro778, Trp780, Ile800, Lys802, Tyr836, Glu849, Val850, Val851, Phe930, and Ile932) for both DSF and Alpelisib showed least RMSF of <2 Å. In both mTOR and PTGS2, the N-terminal residues showed larger fluctuations up to 20 Å and 6 Å, respectively. Whereas the residues involved in ligand binding (refer to Table 2) showed less fluctuation, <3 Å. In the DSF and NFH complexes with MMP9, residues participating with DSF showed larger fluctuations up to 6 Å; however, the ligand was found to be within the binding pocket throughout the simulation period. Figure 7 shows the RMSF plot of C-alpha atom protein targets during a 100 ns MD production run. Supplementary Movies 1–10 show the trajectory visualizations for DSF and standard compounds with their respective targets.

**FIGURE 8:**
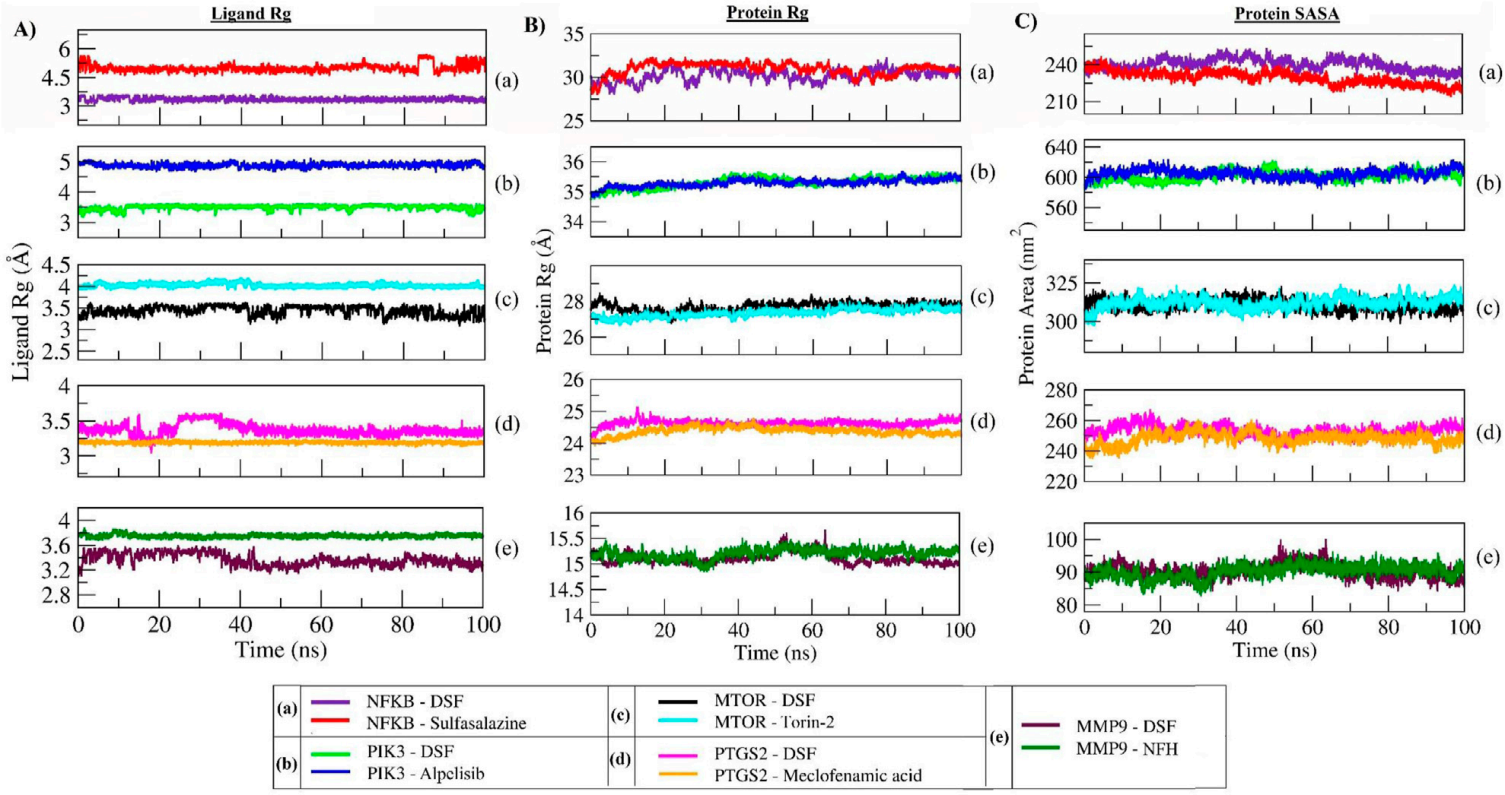
Rg plot of DSF and standard molecules **(A)**, protein atoms **(B)**, and SASA of protein atoms **(C)** during a 100 ns MD simulation.

The radius of gyration (Rg) measures the extendedness of a ligand (as shown in Figure 8A) and stable folding of the protein (as shown in Figure 8B) during simulation through the equivalent to its principal moment of inertia and confirms the stable complex formation. In the DSF- NFκB complex, an increase in the protein Rg during the equilibration period indicated an unfolding behavior and could be the reason for the unstable binding of DSF. However, sulfasalazine was found to be stable at ∼5 Å, and the protein Rg was stable at ∼32 Å, which indicates the ligand is within the binding pocket until ∼80 ns. A steady decrease in the SASA also indicates a stable complex formation with sulfasalazine until ∼80 ns. An increase in the Rg and fluctuating SASA after 80 ns indicates unstable complex formation with sulfasalazine (Figure 8C). DSF formed very stable conformations with PIK3 throughout 100 ns, which is indicated by stable ligand Rg at 3.5 Å and stable protein Rg (35.5 Å) and SASA (∼610 nm^2^). The protein Rg and SASA in other proteins, viz, mTOR (27 Å and 312 nm^2^), PTGS2 (24.5 Å and 250 nm^2^), and MMP9 (15.25 Å and 90 nm^2^) were found to be stable and maintained steady states throughout the 100 ns MD run. Meanwhile, the DSF Rg was found to fluctuate in complex with mTOR, PTGS2, and MMP9, with Rg values ranging from 3 Å to 3.6 Å throughout the 100 ns run. However, DSF was found to be within the binding pocket, and fluctuation could be due to unstable hydrogen bond formation and larger conformational changes (refer to Supplementary Movies).

The number of hydrogen bonds formed between DSF and standard compounds with selected targets was analyzed over the 100 ns MD trajectory. DSF did not form H-bonds with PIK3, mTOR, and MMP9. It formed one H-bond with NFκB and PTGS2; however, these were not consistent throughout the simulation. All the standard molecules with their respective protein targets formed >1 H-bond except for PTGS2, which formed one H-bond. Similarly, we accessed the individual residue contribution in complex formation with DSF and standard compounds. NFκB residues Arg50, Arg30, Gln29, and Glu225 (forming a loop at binding pocket) showed energy contribution of −6.63, −2.44, −0.95, and −0.71 kJ/mol with DSF in stable complex formation during the equilibration period, however, due to the larger fluctuation of the loop, the DSF did not form stable interactions with NFκB, and the residues Glu25, Glu22, Glu49, and Lys28 (beta-sheet residues at binding pocket) opposed the interaction with positive energies of 0.81 kJ/mol, 0.93 kJ/mol, 2.20 kJ/mol, and 2.54 kJ/mol. Similarly, sulfasalazine was found to move to the adjacent binding pocket after 80 ns, and hence, a larger amino acid residual contribution was observed (shown in red).

**FIGURE 9:**
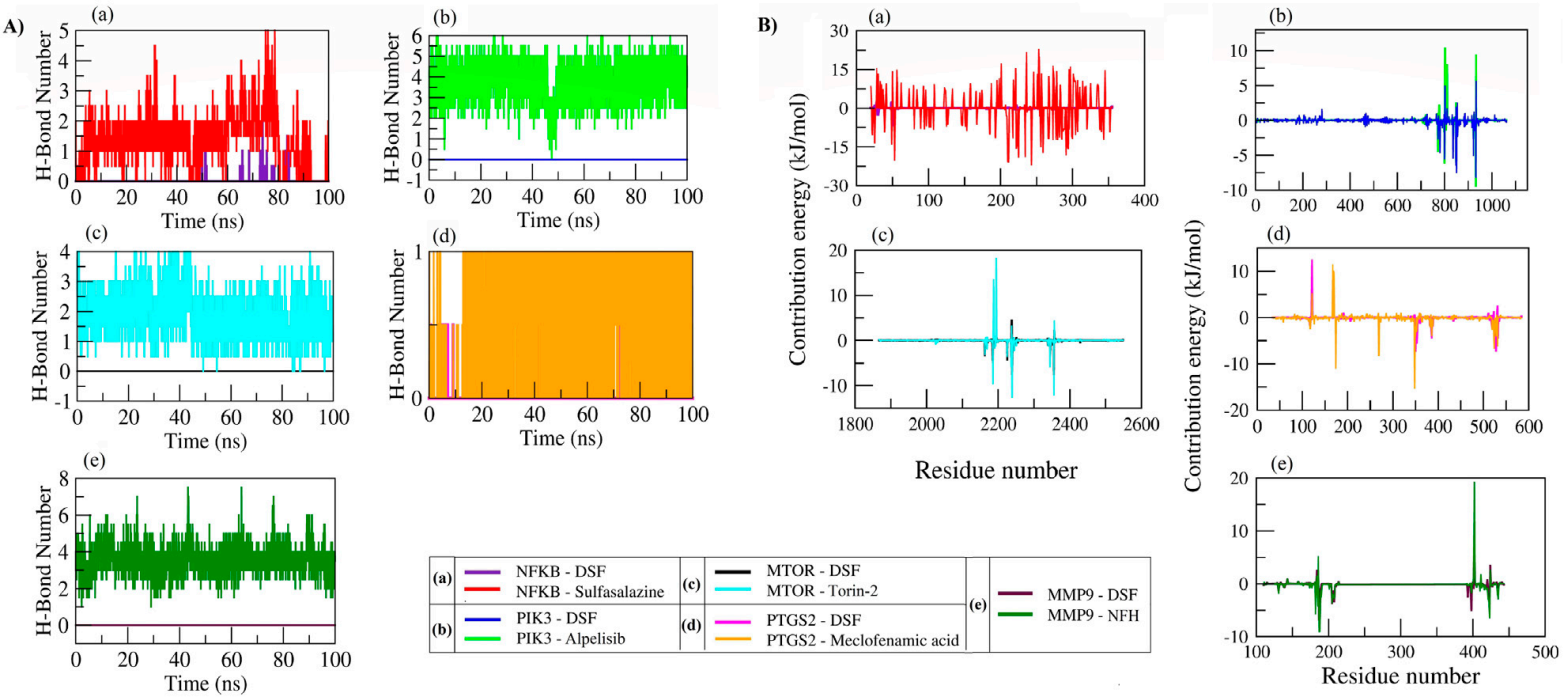
Number of hydrogen bond formations **(A)** and individual residue contribution in stable complex formation **(B)** of proteins with DSF and standard molecules during a 100 ns MD simulation.

DSF formed a stable complex with PIK3 via forming interaction with Ile932, Ile800, Ile848, Met772, and Trp780 by scoring energy contributions of −9.37 kJ/mol, −6.07 kJ/mol, −5.87 kJ/mol, −4.41 kJ/mol, and −2.75 kJ/mol, while the residues Asp810, Asp933, and Lys802 opposed the interaction with positive energy of 7.86 kJ/mol, 9.29 kJ/mol, and 10.28 kJ/mol. In contrast, Alpelisib formed stable contacts with Ile932, Val850, Ile848, Ile800, and Tyr836 of PIK3 via forming energy contributions of −8.14 kJ/mol, −7.48 kJ/mol, −6.37 kJ/mol, −6.37 kJ/mol, and −5.53 kJ/mol and opposing the interaction with Ser854, Lys802, and Asp933. DSF formed stable complexes with mTOR, PTGS2, and MMP9 through the hydrophobic interactions, as DSF did not form hydrogen bonds, and the interactions were consistent at the active site throughout the 100 ns MD simulation as both DSF and standard molecules interactions were overlapped in the decomposition graph (Figure 9) and were also confirmed through visualizing the trajectory (refer to Supplementary Movies for MTOR, PTGS2, and MMP9).

### 3.7 MMPBSA analysis

DSF scored the lowest free binding energies of −29.49 ± 2.37 kcal/mol and −24.34 ± 3.22 kcal/mol with PTGS2 and MMP9 compared to the standard molecules meclofenamic acid (−18.14 ± 3.06 kcal/mol) and NFH (−19.86 ± 3.79 kcal/mol), respectively. DSF also possessed the lowest free binding energies of −19.01 ± 3.03 kcal/mol and −21.26 ± 3.22 kcal/mol with PIK3 and mTOR, whereas their standard molecules Alpelisib and Torin-2 scored −27.60 ± 3.33 kcal/mol and −22.23 ± 4.03 kcal/mol, respectively. During the equilibration period, DSF showed the lowest free binding energy of −10.48 ± 2.99 kcal/mol with NFκB, whereas sulfasalazine scored −7.59 ± 9.59 kcal/mol. Table 3 presents the overall energy contributions: van der Waals electrostatic, polar solvation solvent-accessible surface area, binding free energy of DSF, and standard molecules with their targets.

**TABLE 3:** MMPBSA calculations of the binding free energy and interaction energies of DSF and standard molecules with their respective targets.

Complex name	MMPBSA (kcal/mol)				
	ΔE_VDW_	ΔE_ELE_	ΔG_Sol_	ΔG_Surf_	ΔG_bind_
NFKB - DSF	−18.25 ± 2.87	−10.27 ± 2.24	12.68 ± 3.43	−2.44 ± 0.35	−10.48 ± 2.99
NFKB - Sulfasalazine	−21.22 ± 4.47	−10.83 ± 8.84	26.52 ± 13.20	−2.05 ± 0.63	−7.59 ± 9.59
PIK3 - DSF	−36.48 ± 3.184	−2.89 ± 1.53	24.49 ± 3.89	−4.129 ± 0.21	−19.01 ± 3.03
PIK3 - Alpelisib	−45.92 ± 2.68	−14.39 ± 3.11	37.63 ± 3.96	−4.91 ± 0.17	−27.60 ± 3.33
MTOR - DSF	−30.01 ± 2.72	−2.00 ± 1.27	14.25 ± 2.63	−3.50 ± 0.25	−21.26 ± 3.22
MTOR - Torin-2	−48.34 ± 1.93	−15.09 ± 2.57	45.82 ± 4.48	−4.61 ± 0.17	−22.23 ± 4.03
PTGS2 - DSF	−42.80 ± 2.12	−1.23 ± 0.78	18.84 ± 1.40	−4.30 ± 0.20	−29.49 ± 2.37
PTGS2 - Meclofenamic acid	−38.04 ± 2.72	−19.85 ± 3.20	43.45 ± 2.94	−3.70 ± 0.14	−18.14 ± 3.06
MMP9 - DSF	−32.61 ± 3.11	−6.22 ± 1.79	18.23 ± 2.88	−3.74 ± 0.20	−24.34 ± 3.22
MMP9 - NFH	−33.33 ± 3.79	−6.53 ± 4.88	23.95 ± 5.17	−3.94 ± 0.12	−19.86 ± 3.79

ΔE_VDW_, van der Waals contribution; ΔE_ELE_, electrostatic energy; ΔG_Sol_, polar solvation-free energy; ΔG_Surf_, solvent-accessible surface area; ΔG_bind_ = binding free energy.

### 3.8 Principal component analysis (PCA)-based free energy landscape (FEL) of protein–ligand complexes

**FIGURE 10:**
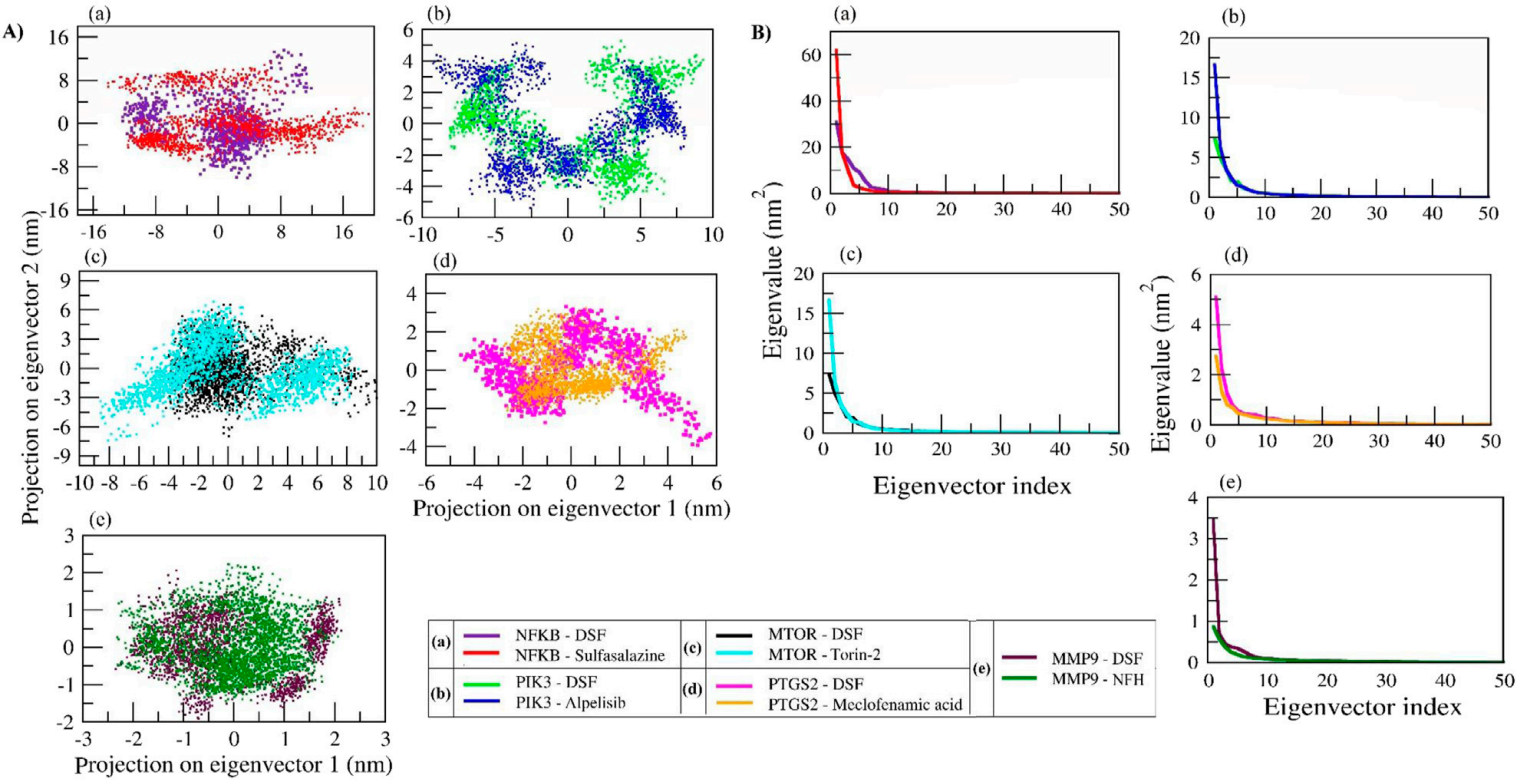
Principal component analysis (PCA) of protein–ligand complexes was performed as follows: **(A)** The collective motion of DSF and standard molecules with their respective protein targets was analyzed by projecting MD trajectories onto the two eigenvectors corresponding to the first two principal components. **(B)** The first 50 eigenvectors were plotted against their eigenvalues for DSF and standard molecules with their respective protein targets.

We analyzed the collective motion captured by the first two principal components (PCs) and plotted 2D projections for PC1 and PC2 (Figures 10A, B). In addition, enhanced sampling was carried out by FEL to analyze trajectories (FEL coordinates at different time intervals are provided in Supplementary Table 11). The DSF and sulfasalazine complexes with NFκB expressed diverse conformational spaces that range from −16 to 17 and eigenvalues of 30 and 61, respectively. As shown in Supplementary Figure S8, DSF was found to be within the binding pocket during the equilibration period, and a few of its conformations were observed until 10 ns. It escaped from the binding pocket at ∼25 ns and remained so throughout the simulation. Sulfasalazine formed two types of conformations (Supplementary Figure S9). At ∼33 ns, it was within the active site, and it moved to an adjacent pocket at ∼75 ns, where it was stabilized until 100 ns. In the MD trajectory of complexes DSF and Alpelisib with PIK3, conformational spaces ranged from −10 to 10 and an eigenvalue of 7.5. Both DSF and Alpelisib were stable at the binding pocket (catalytic region), and diverse conformations of the complexes (Supplementary Figures S10, S11) were due to the significant movement of the longest loop (Ser62 to Ile111) of the p85 regulatory protein. However, the system was stabilized after 50 ns for both complexes.

In the DSF and Torin-2 with mTOR complexes, the DSF complex showed a smaller conformational space range (−4 to 10; EV: 7.5) than Torin-2 (−10 to 10; EV: 17). The FEL indicates that DSF was stabilized from the beginning of MD simulation with slight fluctuation at ∼33 ns, whereas the Torin-2 complex showed three types of diverse conformations at ∼23 ns, 40 ns, and 80 ns and was stabilized from 80 ns to 100 ns (Supplementary Figures S12, 13). In complexes of DSF and meclofenamic acid with PTGS2, meclofenamic acid showed a uniform distribution across the configurational space and clustered in the range of −3 to 5 compared to DSF (−5 to 6). The DSF-PTGS2 complex formed two types of diverse conformations, until 50 ns and after 50 ns. During the equilibration period of 50 ns, the N-terminal residues “Asn1 to Lys50” were found to be unstable, and as a result, two types of conformations were seen, and the system stabilized after 50 ns–100 ns (Supplementary Figures S14, 15). Similarly, DSF and NHF in complexes with MMP9 also showed two types of conformations (Supplementary Figures S16, 17) with PC1 and PC2 ranging from −2.5 to 2, respectively. Both the complexes were stabilized after 50 ns. Herein, we propose that the DSF with NFκB complex has undergone significant conformational changes in the secondary structure during the simulation and was unstable throughout the simulation. However, other complexes, namely, DSF with PIK3, mTOR, MMP9, and PTGS2, were well stabilized and underwent comparatively smaller conformational changes in the secondary structure and hence exhibited compact clusters in the conformations space after the equilibration period.

## 4 Discussion

RF and CKD are consequences of long-term conditions such as diabetes, hypertension, and other polygenic diseases. They have rapidly increased in recent years due to the progressive increase in these conditions (Amadei et al., 1996). Although there is a greater focus on CKD prevention and therapy, many patients ultimately require kidney transplantation or dialysis, as existing treatments can only partially prevent the disease’s progression (Romagnani et al., 2017). Currently, medications for CKD and RF include ACEIs and ARBs (e.g., lisinopril, losartan, valsartan), diuretics (e.g., hydrochlorothiazide), and SGLT2 inhibitors (e.g., empagliflozin) (Momoniat et al., 2019; Johnson and Spurney, 2015). However, these drugs are primarily used for cardiovascular diseases and are associated with adverse drug reactions such as hyperkalemia, hyponatremia, hypotension, electrolyte imbalances, and risks of urinary tract infections, genital infections, and dehydration (Sica, 2005). Therefore, identifying novel compounds with better efficacy and lower risk profiles becomes a priority. Drug repurposing has proven to be an effective method for disease management, and researchers are actively exploring innovative therapeutic uses for existing drugs to accelerate the development of effective treatments. Recently, preclinical tests have proven that DSF reduces renal fibrosis by targeting key genes and molecular pathways involved in RF. Hence, this study intended to explore the overall possible mechanisms of DSF and study the intermolecular interactions with key protein targets. This approach not only saves time and resources but also offers hope for more effective therapies for RF and CKD.

The current study identified approximately 265 protein targets for DSF and 1803 genes associated with RF, and the intersection revealed 78 common proteins, suggesting DSF’s potential role in influencing pathways relevant to RF. The K-means clustering analysis of 78 proteins within the PPI network showed that 58 targets are involved in Cluster 1, indicating highly interconnected proteins in RF.

DSF was found to modulate 138 molecular pathways, of which 21 are directly associated with RF. For instance, the PI3K-Akt signaling pathway, identified as the most enriched with a gene count of 14, plays a pivotal role in cell survival, proliferation, and fibrosis. Studies have shown that PI3K-Akt signaling is often upregulated in fibrotic diseases, making it a key therapeutic target (Wang et al., 2022; Liu et al., 2022). Previous studies have shown that DSF inhibits key components of the PI3K-Akt pathway and reduces cell survival and proliferation, thereby exerting anti-tumor and antifibrotic effects. DSF, through its metabolite diethyldithiocarbamate (DDC), can chelate metal ions such as copper, which are essential for the activity of various enzymes involved in the PI3K-Akt pathway (Zhang et al., 2010). Additionally, by inhibiting the PI3K-Akt pathway, DSF can downregulate downstream signaling molecules such as mTOR, which is involved in protein synthesis and can lead to reduced fibrosis (Dou et al., 2019). In addition, inhibition of these also affects other downstream targets like GSK-3β and FOXO transcription factors, contributing to apoptosis and reduced fibrotic responses (Zheng et al., 2020).

**FIGURE 11:**
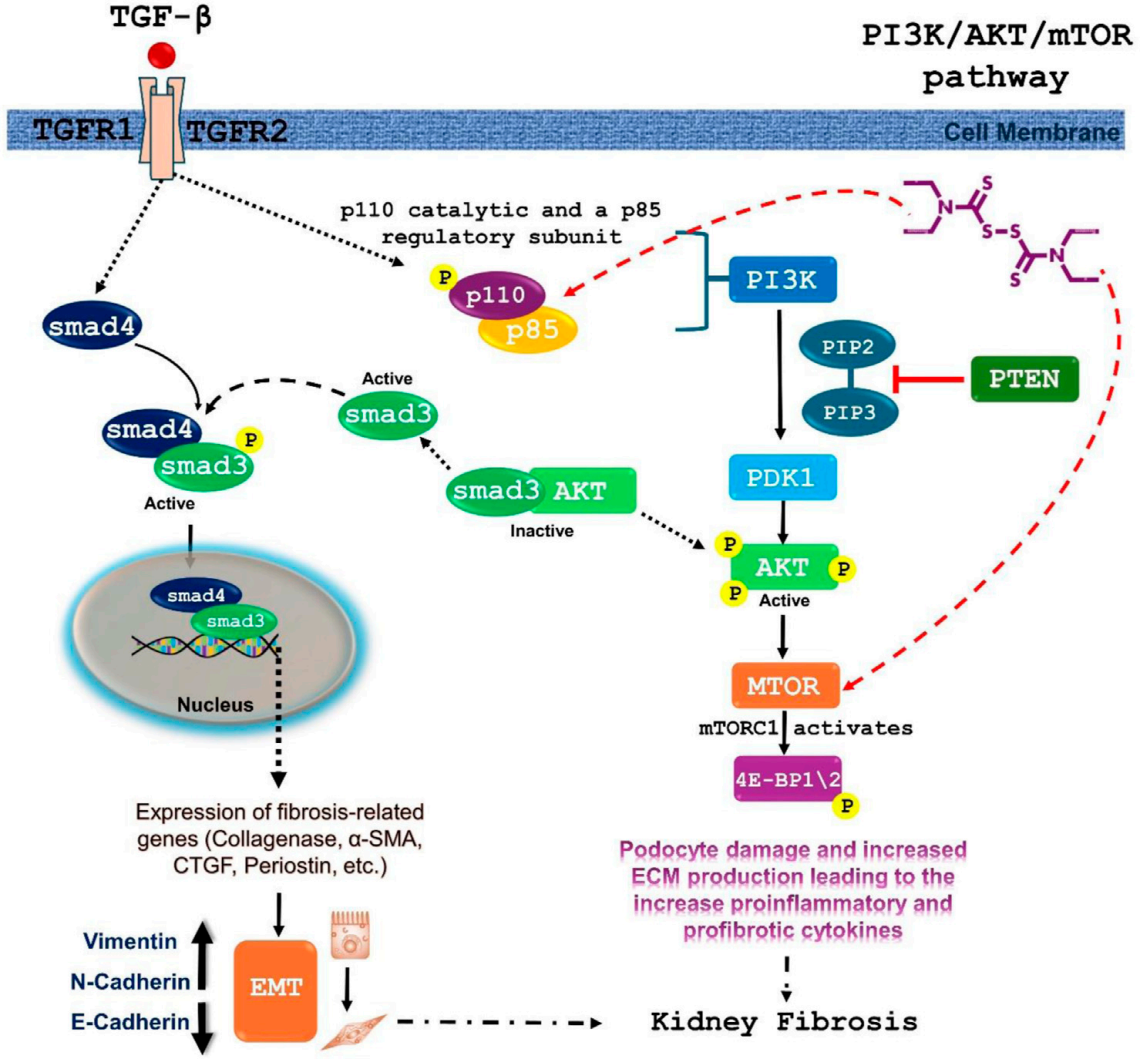
Proposed mechanism of DSF against renal fibrosis via inhibition of PIK3/Akt/mTOR signaling pathway. TGF-β activates the PI3K pathway, leading to the activation of Akt. Activated Akt then stimulates the mTOR pathway. mTOR signaling is involved in cell growth, proliferation, and survival, and its activation can contribute to fibrotic processes. TGF-β binding to its receptors leads to the phosphorylation of SMAD proteins. Phosphorylated SMADs form a complex and translocate to the nucleus, where they regulate the expression of fibrosis-related genes, contributing directly to renal fibrosis. DSF was found to interact stably with both PIK3CA/PIK3R1 and mTOR during 100 ns MD simulation.

Studies in cancer research have demonstrated that DSF can reduce the activity of the PI3K-Akt pathway, leading to decreased tumor growth and increased apoptosis (Zhang et al., 2010). This provides a mechanistic basis for its potential antifibrotic effects. The PI3K-Akt pathway is crucial in fibrotic diseases as it promotes the proliferation and survival of fibroblasts and myofibroblasts, key cells involved in fibrosis (Wang et al., 2022). By inhibiting this pathway, DSF can potentially reduce fibroblast activation and extracellular matrix production, thereby mitigating fibrosis. Figure 11 illustrates the DSF effect in the PI3K/Akt/mTOR signaling pathway. The molecular docking and dynamics studies revealed that DSF formed a stable complex with PIK3 via interactions with residues Ile932, Ile800, Ile848, Met772, and Trp780, which contributed significantly to stability.

The partial double-bond character in the N-C=S (thioamide) and S-C=S (dithioester) groups of DSF plays a crucial role in its structural and functional behavior during interactions with target proteins. These groups impart planarity and rigidity to the molecule, restricting rotational freedom around these bonds. This rigidity enforces a defined molecular conformation that aligns DSF favorably within the binding pockets of different proteins, optimizing hydrophobic and van der Waals interactions. The rigidity of DSF ensures that its hydrophobic surface aligns effectively with these residues, contributing to strong binding affinity during docking. The stability of DSF in the PIK3CA binding site is further supported by the molecular dynamics simulation results, which show consistent stabilization over time, highlighting the importance of rigidity in maintaining these interactions. Alpelisib, a standard molecule, also formed stable interactions with PIK3 but with slightly different residue contributions in the same site, highlighting the specificity of binding sites and interactions based on the ligand positioning.

DSF is a well-known electrophilic molecule, and its interactions with nucleophiles such as cysteine (Cys) were observed for covalent inhibition. Upon cross-checking the molecular docking and dynamics results, no direct interaction between DSF and cysteine residues in the target proteins studied was observed. This suggests that while DSF may not form covalent bonds with cysteine residues under the docking conditions used, its mechanism of action could involve other non-covalent interactions.

Other pathways modulated by DSF, such as JAK-STAT, TNF, Ras, ErbB, p53, mTOR, IL-17, NF-kappa B, AMPK, VEGF, and MAPK, are well-documented in the literature for their roles in inflammation, apoptosis, and fibrosis. NF-κB signaling promotes fibrosis by activating fibroblasts and inducing the expression of fibrogenic factors like TGF-β, which drives ECM production and fibroblast activation (Ren et al., 2024; Lan, 2011). By inhibiting NF-κB, DSF can prevent the upregulation of TGF-β and other fibrotic mediators. DSF is reported to inhibit NF-κB signaling primarily by blocking the degradation of IκB proteins. Normally, IκB proteins bind to NF-κB dimers (such as p65/p50), sequestering them in the cytoplasm. Upon inflammatory stimuli, IκB is degraded, releasing NF-κB dimers to translocate to the nucleus and activate target genes. DSF interferes with this process by chelating zinc, a crucial cofactor for the activity of various enzymes involved in IκB degradation and NF-κB activation (Ren et al., 2024; Giridharan and Srinivasan, 2018).

**FIGURE 12:**
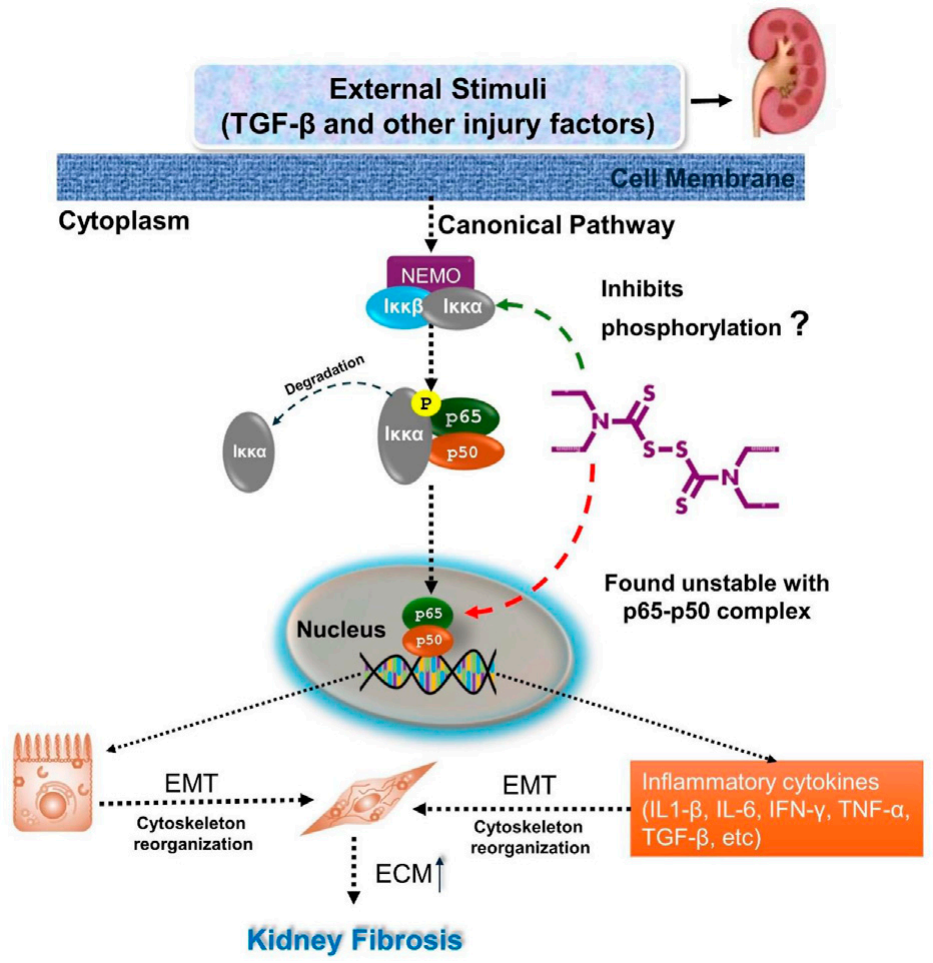
Illustrates the inhibition of the canonical NF-κB signaling pathway by disulfiram in renal fibrosis. TGF-β and other injury factors activate this pathway, leading to the phosphorylation of IκBα. This results in the release of NF-κB complex (p65 and p50), which translocates to the nucleus to promote the expression of genes related to epithelial-mesenchymal transition (EMT) and inflammatory cytokines. Disulfiram inhibits this pathway by preventing the phosphorylation of IκBα (*in vitro*), thereby blocking the release of the NF-κB complex. DSF was found to be unstable within the p65-p50 complex, and further investigations need to be carried out.

DSF’s ability to inhibit NF-κB has been demonstrated in several studies. For instance, DSF has been shown to reduce NF-κB p65 phosphorylation and nuclear translocation in various cell types, leading to decreased expression of NF-κB target genes like TNF-α and IL-6 (Kannappan et al., 2021). In the present study, NFκB p65 residues Arg50, Arg30, Gln29, and Glu225 contributed significantly to the binding energy during equilibration with DSF, indicating stable complex formation. However, after ∼25 ns, the DSF was found to be unstable as it moved out of the binding pocket until 100 ns. Here, the rigidity of DSF helps maintain a defined conformation during docking. The molecule’s restricted flexibility may hinder its ability to adapt to conformational changes in the binding site during molecular dynamics. This is evident in the simulation results, where DSF moves out of the active site over time, suggesting that the rigidity, while beneficial for initial binding, limits its adaptability in this specific protein environment. Hence, further intermolecular interaction of DSF with IκB proteins needs to be verified (as represented in Figure 12).

DSF also influences other fibrotic and inflammatory pathways, such as PI3K-Akt and MAPK, which often interact with NF-κB signaling. For example, PI3K-Akt signaling can activate NF-κB by promoting the degradation of IκB proteins, while MAPK pathways can enhance NF-κB activity. DSF’s effects on these pathways have been shown to contribute to its antifibrotic and anti-inflammatory properties. For instance, DSF-induced inhibition of PI3K-Akt signaling leads to decreased NF-κB activity, illustrating a broader antifibrotic effect (Kanai et al., 2010). In animal models of inflammation, DSF treatment resulted in reduced levels of TNF-α and IL-6, demonstrating that DSF effectively suppresses NF-κB-driven inflammatory responses (Huang et al., 2022; Yoshiyasu et al., 2024).

The JAK-STAT pathway is involved in cytokine signaling and has been implicated in chronic kidney disease and fibrosis. In RF, the JAK-STAT pathway mediates the effects of cytokines like IL-6, IL-11, and others, which are involved in fibroblast activation and extracellular matrix production (Liu et al., 2023; Malemud and Pearlman, 2009). Disulfiram has been shown to inhibit the activation of JAKs, leading to decreased phosphorylation and activation of STAT proteins, and hence, DSF can reduce the inflammatory responses that contribute to fibrosis (Kim et al., 2017).

**FIGURE 13:**
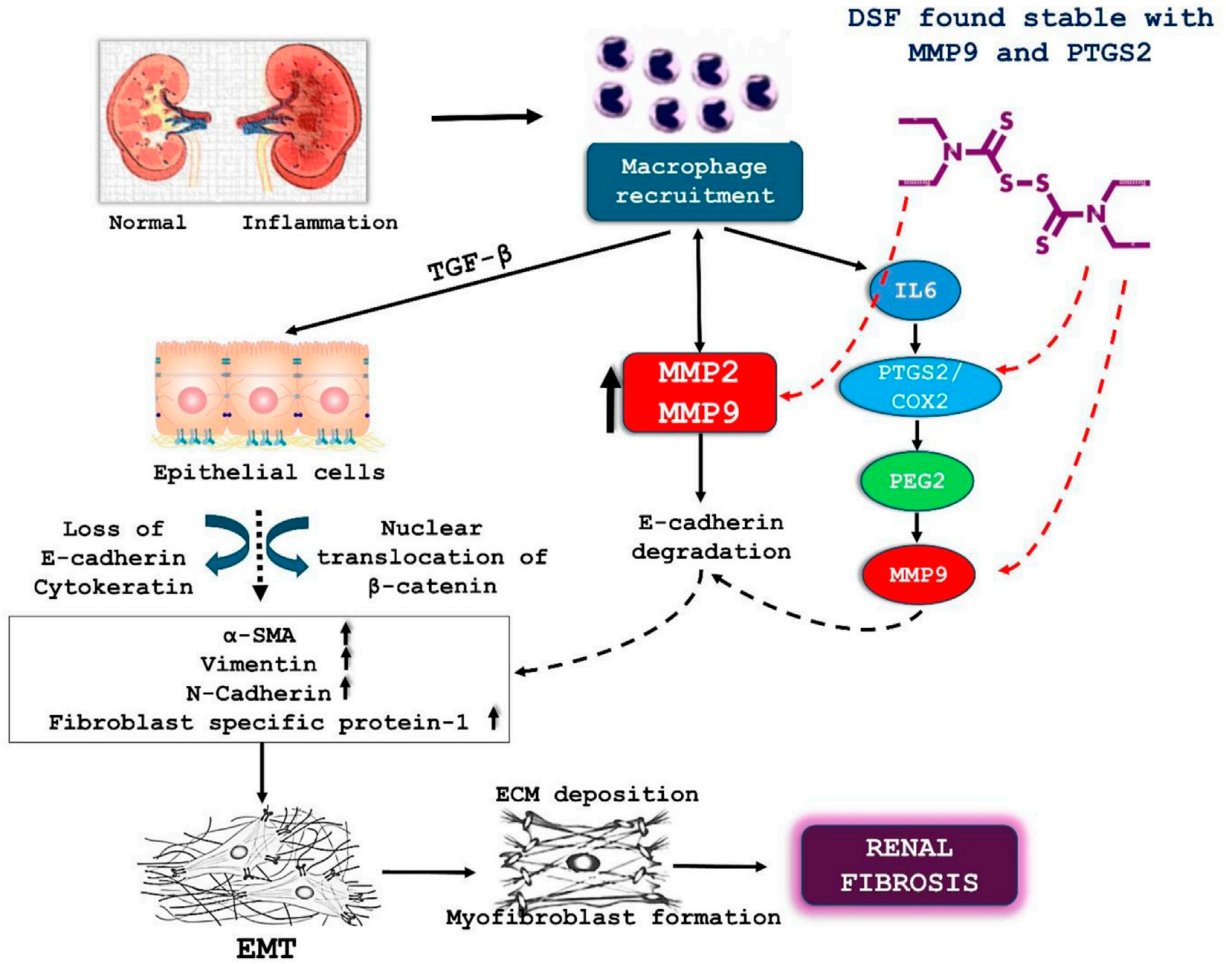
Illustrate the role of DSF in mitigating renal fibrosis through its interaction with MMP9 and PTGS2/COX2. This cytokine induces the loss of E-cadherin and cytokeratin in epithelial cells, promoting the nuclear translocation of β-catenin. This process leads to the upregulation of markers such as α-SMA, vimentin, and N-cadherin, contributing to EMT and the formation of myofibroblasts, which produce excessive ECM, resulting in renal fibrosis. DSF is shown to interact stably with MMP9 and PTGS2/COX2. By stabilizing these molecules, DSF inhibits the downstream effects (degradation of E-cadherin and activation of MMP9), thus reducing ECM deposition and myofibroblast formation. This inhibition of EMT and ECM deposition ultimately helps to prevent the progression of renal fibrosis.

The network analysis identified the hub genes PIK3R1, MAPK3, NFKB1, and MTOR, which are central to the regulation of fibrosis and inflammation. Previous literature on DSF’s modulation of key RF therapeutic targets such as PTGS2, MMP9, MMP2, and SMAD4 suggests its potential to influence these targets synergistically. MMP9 is involved in extracellular matrix remodeling, and its inhibition has been shown to mitigate fibrosis (La Russa et al., 2024). DSF has been shown to reduce MMP9 expression and activity in osteosarcoma cells (Cho et al., 2007). DSF-induced inhibition of MMP9 activity is accompanied by changes in other fibrotic markers and pathways. The molecular docking and dynamic studies revealed that DSF with mTOR, PTGS2, and MMP9 formed stable complexes through hydrophobic interactions rather than H-bonds. The mechanistic effect of DSF in the prevention of renal fibrosis via inhibiting MMP9 and PTGS2 is illustrated in Figure 13. The consistency of these interactions throughout the 100 ns MD simulation, as confirmed by the decomposition graph and trajectory analysis by FEL, suggests that hydrophobic interactions play a critical role in DSF’s binding mechanism. The current study suggests that DSF’s therapeutic effects in RF extend beyond inhibiting PI3K, mTOR, MMP9, and PTGS2. It can also modulate additional signaling pathways such as TNF, Ras, ErbB, p53, IL-17, AMPK, and VEGF.

Disulfiram (DSF) and its metabolites have been found to have various biological activities, including antibacterial and anti-fibrosis effects. Disulfiram metabolites, including diethyldithiocarbamate (DDC), carbon disulfide (CS2), S-methyl N,N-diethylthiocarbamate sulfoxide (DETC-sulfoxide), S-methyl N,N-diethylthiocarbamate sulfone (DETC-sulfone), diethylthiomethylcarbamate, S-methyl-DDC, methyl diethylthiocarbamate sulfoxide, and methyl diethylthiocarbamate sulfone, play a crucial role in its pharmacological effects (PharmGKB, 2023). DDC is the primary metabolite and has been shown to contribute to DSF’s activity through metal chelation and interaction with cellular pathways (Mays et al., 1996). DETC-sulfoxide and DETC-sulfone, which are oxidative metabolites of DDC, may also be involved in DSF’s effects (Shen.et al., 2024; Sedlacek et al., 2014). Other metabolites, such as S-methyl-DDC and methylated forms of DDC, might exhibit enhanced biological activity and influence protein interactions and enzymatic pathways (Mays et al., 1996).

Studies have investigated the antibacterial activity of disulfiram and its metabolites, with results indicating that most DSF metabolites do not possess significant antibacterial activity, except for DDTC in *Bacillus anthracis*. The combination of DSF and DDTC with standard antibiotics has been shown to have synergistic effects (Mays et al., 1996; Zaldívar-Machorro et al., 2011). In addition, the effects of DSF metabolites on rat liver mitochondrial low Km ALDH have been examined, with MeDTC sulfoxide and MeDTC sulfone found to inhibit ALDH activity (Nagendra et al., 1994). The mechanism of action of disulfiram involves the inhibition of hepatic aldehyde dehydrogenase (ALDH), with the ultimate inhibitor thought to be a metabolite of disulfiram. MeDTC sulfoxide is considered a better candidate for the ultimate active metabolite of disulfiram due to its stability and ability to diffuse from a distant site of formation and react with ALDH (Mays et al., 1996).

Overall, the metabolites of disulfiram play a significant role in its pharmacological effects, and understanding their mechanisms of action is essential for comprehensively evaluating DSF’s therapeutic and toxic profile. Further studies are needed to investigate the roles of these metabolites in DSF’s activity and to explore their potential applications in the treatment of various diseases, including renal fibrosis.

## 5 Conclusion

In conclusion, this comprehensive study delves into the intricate dynamics of DSF with RF-associated protein targets. Notably, NFκB, PIK3R1/CA, MTOR, PTGS2, and MMP9 have emerged as central targets of DSF involved in the key signaling cascades crucial to the RF progression. DSF may showcase diverse pharmacological therapeutic effects by targeting multiple enzymes and pathways. DSF binding to the core genes has been explored using molecular docking, simulation, and FEL investigations; however, its agonistic or antagonistic properties are yet unknown. Therefore, additional experimental testing is required to grasp its true nature in RF. DSF affects the identified genes involved in inflammation and fibrogenesis associated with CKD, resulting in the inhibition of pro-inflammatory cytokines and profibrotic factors that cause chronic inflammation and fibroblast activation. DSF affects renal fibroblast survival and proliferation, reducing ECM production and deposition and inhibiting fibrogenic proteins. However, the predicted complexity of the DSF-RF protein network extends beyond NFκB, PIK3R1/CA, mTOR, PTGS2, and MMP9 and warrants further investigations, particularly concerning its true biological activities, via *in vitro* and *in vivo* studies.

## Data Availability

The original contributions presented in the study are included in the article/Supplementary Material; further inquiries can be directed to the corresponding author/s.

## References

[B1] Abbas N. A. T. El Salem A. Awad M. M. (2018). Empagliflozin, SGLT2 inhibitor, attenuates renal fibrosis in rats exposed to unilateral ureteric obstruction: potential role of klotho expression. Naunyn Schmiedeb. Arch. Pharmacol. 391 (12), 1347–1360. 10.1007/s00210-018-1544-y 30090949

[B2] Amadei A. Linssen A. B. de Groot B. L. Van Aalten D. M. Berendsen H. J. (1996). An efficient method for sampling the essential subspace of proteins. J. Biomol. Struct. Dyn. 13, 615–625. 10.1080/07391102.1996.10508874 8906882

[B3] Bhandare V. V. Ramaswamy A. (2018). The proteinopathy of D169G and K263E mutants at the RNA Recognition Motif (RRM) domain of tar DNA-binding protein (tdp43) causing neurological disorders: a computational study. J. Biomol. Struct. Dyn. 36, 1075–1093. 10.1080/07391102.2017.1310670 28330421

[B4] Chen X. Ji Z. L. Chen Y. Z. (2002). TTD: therapeutic target database. Nucleic Acids Res. 30, 412–415. 10.1093/nar/30.1.412 11752352 PMC99057

[B5] Cho H. J. Lee T. S. Park J. B. Park K. K. Choe J. Y. Sin D. I. (2007). Disulfiram suppresses invasive ability of osteosarcoma cells via the inhibition of MMP-2 and MMP-9 expression. BMB Rep. 40, 1069–1076. 10.5483/bmbrep.2007.40.6.1069 18047805

[B6] Cruz-Solbes A. S. Youker K. (2017). Epithelial to mesenchymal transition (EMT) and endothelial to mesenchymal transition (EndMT): role and implications in kidney fibrosis. Kidney Dev. Dis. 60, 345–372. 10.1007/978-3-319-51436-9_13 28409352

[B7] Daza-Arnedo R. Rico-Fontalvo J. E. Pájaro-Galvis N. Leal-Martínez V. Abuabara-Franco E. Raad-Sarabia M. (2021). Dipeptidyl peptidase-4 inhibitors and diabetic kidney disease: a narrative review. Kidney Med. 3 (6), 1065–1073. 10.1016/j.xkme.2021.07.007 34939016 PMC8664739

[B8] DeLano W. L. (2002). Pymol: an open-source molecular graphics tool. CCP4 Newsl. Protein Crystallogr. 40 (1), 82–92.

[B9] Dou F. Liu Y. Liu L. Wang J. Sun T. Mu F. (2019). Aloe-emodin ameliorates renal fibrosis via inhibiting PI3K/Akt/mTOR signaling pathway *in vivo* and *in vitro* . Rejuvenation Res. 22, 218–229. 10.1089/rej.2018.2104 30215298

[B10] Edeling M. Ragi G. Huang S. Pavenstädt H. Susztak K. (2016). Developmental signalling pathways in renal fibrosis: the roles of Notch, Wnt and Hedgehog. Nat. Rev. Nephrol. 12 (7), 426–439. 10.1038/nrneph.2016.54 27140856 PMC5529143

[B11] Ekinci E. Rohondia S. Khan R. Dou Q. P. (2019). Repurposing disulfiram as an anti-cancer agent: updated review on literature and patents. Recent Pat. Anti-Cancer Drug Discov. 14, 113–132. 10.2174/1574892814666190514104035 31084595

[B12] Elliott J. H. McMahon J. H. Chang C. C. Lee S. A. Hartogensis W. Bumpus N. (2015). Short-term administration of disulfiram for reversal of latent HIV infection: a phase 2 dose-escalation study. Lancet HIV 2, e520–e529. 10.1016/S2352-3018(15)00226-X 26614966 PMC5108570

[B13] Fenton S. H. Benigni M. S. (2014). Projected impact of the ICD-10-CM/PCS conversion on longitudinal data and the joint commission Core measures. Perspect. Health Inf. Manag. 11, 1g. PMC4142515 25214824

[B14] Fillmore N. Bell S. Shen C. Nguyen V. La J. Dubreuil M. (2021). Disulfiram use is associated with lower risk of COVID-19: a retrospective cohort study. PLoS One 16, e0259061. 10.1371/journal.pone.0259061 34710137 PMC8553043

[B15] Francis A. Harhay M. N. Ong A. Tummalapalli S. L. Ortiz A. Fogo A. B. (2024). Chronic kidney disease and the global public health agenda: an international consensus. Nat. Rev. Nephrol. 20, 473–485. 10.1038/s41581-024-00820-6 38570631

[B16] Fried L. F. Petruski-Ivleva N. Folkerts K. Schmedt N. Velentgas P. Kovesdy C. P. (2021). ACE inhibitor or ARB treatment among patients with diabetes and chronic kidney disease. Am. J. Manag. Care 27 (Dec 3), S360–S368. 10.37765/ajmc.2021.88806 34878753

[B18] Furet P. Guagnano V. Fairhurst R. A. Imbach-Weese P. Bruce I. Knapp M. (2013). Discovery of NVP-BYL719 a potent and selective phosphatidylinositol-3 kinase alpha inhibitor selected for clinical evaluation. Bioorg. Med. Chem. Lett. 23, 3741–3748. 10.1016/j.bmcl.2013.05.007 23726034

[B19] Gallo K. Goede A. Preissner R. Gohlke B. O. (2022). SuperPred 3.0: drug classification and target prediction—a machine learning approach. Nucleic Acids Res. 50, W726–W731. 10.1093/nar/gkac297 35524552 PMC9252837

[B20] Giridharan S. S. Srinivasan M. (2018). Mechanisms of NF-κB p65 and strategies for therapeutic manipulation. J. Inflamm. Res. 11, 407–419. 10.2147/JIR.S140188 30464573 PMC6217131

[B21] Hadda V. Guleria R. (2020). Antifibrotic drugs for idiopathic pulmonary fibrosis: what we should know? Indian J. Med. Res. 152, 177–180. 10.4103/ijmr.IJMR_90_20 33107479 PMC7881818

[B22] Han D. Wu G. Chang C. Zhu F. Xiao Y. Li Q. (2015). Disulfiram inhibits TGF-β-induced epithelial-mesenchymal transition and stem-like features in breast cancer via ERK/NF-κB/Snail pathway. Oncotarget 6, 40907–40919. 10.18632/oncotarget.5723 26517513 PMC4747377

[B23] He J. Xu Y. Koya D. Kanasaki K. (2013). Role of the endothelial-to-mesenchymal transition in renal fibrosis of chronic kidney disease. Clin. Exp. Nephrol. 17, 488–497. 10.1007/s10157-013-0781-0 23430391

[B24] Huang J. Wei S. Peng Z. Xiao Z. Yang Y. Liu J. (2022). Disulfiram attenuates lipopolysaccharide-induced acute kidney injury by suppressing oxidative stress and NLRP3 inflammasome activation in mice. J. Pharm. Pharmacol. 74, 259–267. 10.1093/jpp/rgab171 34923585

[B25] Huang R. Fu P. Ma L. (2023). Kidney fibrosis: from mechanisms to therapeutic medicines. Signal Transduct. Target Ther. 8 (1), 129. 10.1038/s41392-023-01379-7 36932062 PMC10023808

[B26] Jacobs M. D. Harrison S. C. (1998). Structure of an IkappaBalpha/NF-kappaB complex. Cell 95, 749–758. 10.1016/s0092-8674(00)81698-0 9865693

[B27] Jacobs M. E. de Vries D. K. Engelse M. A. Dumas S. J. Rabelink T. J. (2024). Endothelial to mesenchymal transition in kidney fibrosis. Nephrol. Dial. Transpl. 39 (5), 752–760. 10.1093/ndt/gfad238 37968135

[B28] Jiao Y. Hannafon N. Ding W. Q. (2016). Disulfiram's anticancer activity: evidence and mechanisms. Anti-Cancer Agents Med. Chem. 16, 1378–1384. 10.2174/1871520615666160504095040 27141876

[B29] Johnson S. A. Spurney R. F. (2015). Twenty years after ACEIs and ARBs: emerging treatment strategies for diabetic nephropathy. Am. J. Physiol. Ren. Physiol. 309, F807–F820. 10.1152/ajprenal.00266.2015 PMC4652076 26336162

[B30] Kanai K. Itoh N. Yoshioka K. Yonezawa T. Ikadai H. Hori Y. (2010). Inhibitory effects of oral disulfiram on endotoxin-induced uveitis in rats. Curr. Eye Res. 35, 892–899. 10.3109/02713683.2010.495442 20858110

[B31] Kanasaki K. (2018). The role of renal dipeptidyl peptidase-4 in kidney disease: renal effects of dipeptidyl peptidase-4 inhibitors with a focus on linagliptin. Clin. Sci. 132 (4), 489–507. 10.1042/CS20180031 PMC5828949 29491123

[B32] Kanehisa M. Furumichi M. Tanabe M. Sato Y. Morishima K. (2017). KEGG: new perspectives on genomes, pathways, diseases and drugs. Nucleic Acids Res. 45, D353-D361–D361. 10.1093/nar/gkw1092 27899662 PMC5210567

[B33] Kannappan V. Ali M. Small B. Rajendran G. Elzhenni H. Taj H. (2021). Recent advances in repurposing disulfiram and disulfiram derivatives as copper-dependent anticancer agents. Front. Mol. Biosci. 8, 741316. 10.3389/fmolb.2021.741316 34604310 PMC8484884

[B34] Khanal P. Patil V. S. Patil B. M. Bhattacharya K. Shrivastava A. K. Chaudhary R. K. (2023). The marijuana-schizophrenia multifaceted nexus: connections and conundrums towards neurophysiology. Comput. Biol. Chem. 107, 107957. 10.1016/j.compbiolchem.2023.107957 37729848

[B35] Kim S. Chen J. Cheng T. Gindulyte A. He J. He S. (2019). PubChem 2019 update: improved access to chemical data. Nucleic Acids Res. 47, D1102-D1109–D1109. 10.1093/nar/gky1033 30371825 PMC6324075

[B36] Kim Y. J. Kim J. Y. Lee N. Oh E. Sung D. Cho T. M. (2017). Disulfiram suppresses cancer stem-like properties and STAT3 signaling in triple-negative breast cancer cells. Biochem. Biophys. Res. Commun. 486, 1069–1076. 10.1016/j.bbrc.2017.03.164 28373070

[B37] Kovesdy C. P. (2022). Epidemiology of chronic kidney disease: an update 2022. Kidney Int. Suppl. 12, 7–11. 10.1016/j.kisu.2021.11.003 PMC9073222 35529086

[B38] Krivák R. Hoksza D. (2018). P2Rank: machine learning based tool for rapid and accurate prediction of ligand binding sites from protein structure. J. Cheminform. 10, 39. 10.1186/s13321-018-0285-8 30109435 PMC6091426

[B39] Kuhn M. von Mering C. Campillos M. Jensen L. J. Bork P. (2007). STITCH: interaction networks of chemicals and proteins. Nucleic Acids Res. 36, D684–D688. 10.1093/nar/gkm795 18084021 PMC2238848

[B40] Kumari R. Kumar R. Lynn A. (2014). g_mmpbsa: a GROMACS tool for high-throughput MM-PBSA calculations. J. Chem. Inf. Model. 54, 1951–1962. 10.1021/ci500020m 24850022

[B41] Lagunin S. Ivanov S. Rudik A. Filimonov D. Poroikov V. (2013). DIGEP-Pred: web service for *in silico* prediction of drug-induced gene expression profiles based on structural formula. Bioinformatics 29, 2062–2063. 10.1093/bioinformatics/btt322 23740741

[B103] Lameire N. H. Levin A. Kellum J. A. Cheung M. Jadoul M. Winkelmayer W. C. (2021). Harmonizing acute and chronic kidney disease definition and classification: report of a Kidney Disease: Improving Global Outcomes (KDIGO) consensus conference. Kidney International 100 (3), 516–526. 10.1016/j.kint.2021.06.028 34252450

[B42] Lan H. Y. (2011). Diverse roles of TGF-β/Smads in renal fibrosis and inflammation. Int. J. Biol. Sci. 7, 1056–1067. 10.7150/ijbs.7.1056 21927575 PMC3174390

[B43] La Russa A. Serra R. Faga T. Crugliano G. Bonelli A. Coppolino G. (2024). Kidney fibrosis and matrix metalloproteinases (MMPs). Front. Biosci.-Landmark 29, 192. 10.31083/j.fbl2905192 38812325

[B44] Lee J. Kim S. H. (2009). PDB editor: a user-friendly Java-based Protein Data Bank file editor with a GUI. Acta Crystallogr. D. Biol. Crystallogr. 65, 399–402. 10.1107/S090744490900451X 19307724

[B45] Li J. Liu H. Takagi S. Nitta K. Kitada M. Srivastava S. P. (2020). Renal protective effects of empagliflozin via inhibition of EMT and aberrant glycolysis in proximal tubules. JCI insight 5 (6), e129034. 10.1172/jci.insight.129034 32134397 PMC7213787

[B46] Li N. Zhang J. Yan X. Zhang C. Liu H. Shan X. (2017). SIRT3-KLF15 signaling ameliorates kidney injury induced by hypertension. Oncotarget 8 (24), 39592–39604. 10.18632/oncotarget.17165 28465484 PMC5503635

[B47] Li Y. Chen F. Chen J. Chan S. He Y. Liu W. (2020). Disulfiram/copper induces antitumor activity against both nasopharyngeal cancer cells and cancer-associated fibroblasts through ROS/MAPK and ferroptosis pathways. Cancers 12, 138. 10.3390/cancers12010138 31935835 PMC7017005

[B48] Li Y. Chen S. Yang Q. Liu X. Zhou W. Kang T. (2024). The ANGPTL4-HIF-1α loop: a critical regulator of renal interstitial fibrosis. J. Transl. Med. 22 (1), 649. 10.1186/s12967-024-05466-3 38992710 PMC11241841

[B49] Lindahl E. Hess B. Van Der Spoel D. (2001). GROMACS 3.0: a package for molecular simulation and trajectory analysis. Mol. Modell. Annu. 7, 306–317. 10.1007/s008940100045

[B50] Liu B. Deng J. Jie X. Lu F. Liu X. Zhang D. (2022). Protective effects of the Bupi Yishen formula on renal fibrosis through PI3K/AKT signaling inhibition. J. Ethnopharmacol. 293, 115242. 10.1016/j.jep.2022.115242 35367329

[B51] Liu J. Wang F. Luo F. (2023). The role of JAK/STAT pathway in fibrotic diseases: molecular and cellular mechanisms. Biomolecules 13, 119. 10.3390/biom13010119 36671504 PMC9855819

[B52] Liu T. Lin Y. Wen X. Jorissen R. N. Gilson M. K. (2007). BindingDB: a web-accessible database of experimentally determined protein–ligand binding affinities. Nucleic Acids Res. 35, D198–D201. 10.1093/nar/gkl999 17145705 PMC1751547

[B53] Liu Z. Mi M. Zheng X. Zhang C. Zhu F. Liu T. (2021). miR-30a/SOX4 double negative feedback loop is modulated by disulfiram and regulates EMT and stem cell-like properties in breast cancer. J. Cancer 12, 5053–5065. 10.7150/jca.57752 34234874 PMC8247377

[B54] Lu J. Chen L. Yin J. Huang T. Bi Y. Kong X. (2016). Identification of new candidate drugs for lung cancer using chemical–chemical interactions, chemical–protein interactions and a K-means clustering algorithm. J. Biomol. Struct. Dyn. 34, 906–917. 10.1080/07391102.2015.1060161 26849843

[B55] Malemud C. J. Pearlman E. (2009). Targeting JAK/STAT signaling pathway in inflammatory diseases. Curr. Signal Transduct. Ther. 4, 201–221. 10.2174/157436209789057467

[B56] Mays D. C. Nelson A. N. Lam‐Holt J. Fauq A. H. Lipsky J. J. (1996). S‐Methyl‐N, N‐diethylthiocarbamate sulfoxide and S‐methyl‐N, N‐diethylthiocarbamate sulfone, two candidates for the active metabolite of disulfiram. Alcohol Clin. Exp. Res. 20 (3), 595–600. 10.1111/j.1530-0277.1996.tb01099.x 8727261

[B57] Meng X. M. Tang P. M. Li J. Lan H. Y. (2015). TGF-β/Smad signaling in renal fibrosis. Front. Physiol. 6, 82. 10.3389/fphys.2015.00082 25852569 PMC4365692

[B58] Mering C. V. Huynen M. Jaeggi D. Schmidt S. Bork P. Snel B. (2003). STRING: a database of predicted functional associations between proteins. Nucleic Acids Res. 31, 258–261. 10.1093/nar/gkg034 12519996 PMC165481

[B59] Momoniat T. Ilyas D. Bhandari S. (2019). ACE inhibitors and ARBs: managing potassium and renal function. Clevel. Clin. J. Med. 86, 601–607. 10.3949/ccjm.86a.18024 31498767

[B60] Nady M. E. Abd El-Raouf O. M. El-Sayed E. S. (2024). Linagliptin mitigates TGF-β1 mediated epithelial–mesenchymal transition in tacrolimus-induced renal interstitial fibrosis via Smad/ERK/P38 and HIF-1α/LOXL2 signaling pathways. Biol. Pharm. Bull. 47 (5), 1008–1020. 10.1248/bpb.b23-00737 38797693

[B61] Nagendra S. N. Madan A. Faiman M. D. (1994). S-methyl N, N-diethylthiolcarbamate sulfone, an *in vitro* and *in vivo* inhibitor of rat liver mitochondrial low Km aldehyde dehydrogenase. Biochem. Pharmacol. 47 (8), 1465–1467. 10.1016/0006-2952(94)90350-6 8185656

[B62] Nitta K. Shi S. Nagai T. Kanasaki M. Kitada M. Srivastava S. P. (2016). Oral administration of N-acetyl-seryl-aspartyl-lysyl-proline ameliorates kidney disease in both type 1 and type 2 diabetic mice via a therapeutic regimen. Biomed. Res. Int. 2016 (1), 9172157. 10.1155/2016/9172157 27088094 PMC4818806

[B63] O’Callaghan-Gordo C. Shivashankar R. Anand S. Ghosh S. Glaser J. Gupta R. (2019). Prevalence of and risk factors for chronic kidney disease of unknown aetiology in India: secondary data analysis of three population-based cross-sectional studies. BMJ Open 9, e023353. 10.1136/bmjopen-2018-023353 PMC6429742 30850400

[B64] Okano T. Kobayashi T. Yasuma T. D’Alessandro-Gabazza C. N. Toda M. Fujimoto H. (2020). Low-dose of intrapulmonary pirfenidone improves human transforming growth factorβ1-driven lung fibrosis. Front. Pharmacol. 11, 593620. 10.3389/fphar.2020.593620 33390975 PMC7774321

[B65] Orlando B. J. Malkowski M. G. (2016). Crystal structure of rofecoxib bound to human cyclooxygenase-2. Acta Crystallogr. F. Struct. Biol. Commun. 72, 772–776. 10.1107/S2053230X16014230 27710942 PMC5053162

[B66] Panizo S. Martínez-Arias L. Alonso-Montes C. Cannata P. Martín-Carro B. Fernández-Martín J. L. (2021). Fibrosis in chronic kidney disease: pathogenesis and consequences. Int. J. Mol. Sci. 22, 408. 10.3390/ijms22010408 33401711 PMC7795409

[B67] Pardali E. Sanchez-Duffhues G. Gomez-Puerto M. C. Ten Dijke P. (2017). TGF-β-induced endothelial-mesenchymal transition in fibrotic diseases. Int. J. Mol. Sci. 18 (10), 2157. 10.3390/ijms18102157 29039786 PMC5666838

[B68] Patil V. S. Harish D. R. Charla R. Vetrivel U. Jalalpure S. S. Bhandare V. V. (2023a). Structural insights into modeling of hepatitis B virus reverse transcriptase and identification of its inhibitors from potential medicinal plants of Western Ghats: an *in silico* and *in vitro* study. J. Biomol. Struct. Dyn. 27, 11731–11749. 10.1080/07391102.2023.2264400 37811543

[B69] Patil V. S. Harish D. R. Sampat G. H. Roy S. Jalalpure S. S. Khanal P. (2023b). System biology investigation revealed lipopolysaccharide and alcohol-induced hepatocellular carcinoma resembled hepatitis B virus immunobiology and pathogenesis. Int. J. Mol. Sci. 24, 11146. 10.3390/ijms241311146 37446321 PMC10342420

[B70] PharmGKB (2023). Disulfiram pathway, pharmacokinetics. Pathway: PA166287601. PharmGKB. Available at: https://www.pharmgkb.org/pathway/PA166287601 (Accessed January 9, 2025).

[B71] Quan Y. Park W. Jin J. Kim W. Park S. K. Kang K. P. (2020). Sirtuin 3 activation by honokiol decreases unilateral ureteral obstruction-induced renal inflammation and fibrosis via regulation of mitochondrial dynamics and the renal NF-κBTGF-β1/Smad signaling pathway. Int. J. Mol. Sci. 21, 402. 10.3390/ijms21020402 31936371 PMC7014106

[B72] Rayego-Mateos S. Campillo S. Rodrigues-Diez R. R. Tejera-Muñoz A. Marquez-Exposito L. Goldschmeding R. (2021). Interplay between extracellular matrix components and cellular and molecular mechanisms in kidney fibrosis. Clin. Sci. 135, 1999–2029. 10.1042/CS20201016 34427291

[B73] Ren N. Wang W. F. Zou L. Zhao Y. L. Miao H. Zhao Y. Y. (2024). The nuclear factor kappa B signaling pathway is a master regulator of renal fibrosis. Front. Pharmacol. 14, 1335094. 10.3389/fphar.2023.1335094 38293668 PMC10824958

[B74] Romagnani P. Remuzzi G. Glassock R. Levin A. Jager K. J. Tonelli M. (2017). Chronic kidney disease. Nat. Rev. Dis. Prim. 3, 17088–17124. 10.1038/nrdp.2017.88 29168475

[B75] Rowsell S. Hawtin P. Minshull C. A. Jepson H. Brockbank S. M. Barratt D. G. (2002). Crystal structure of human MMP9 in complex with a reverse hydroxamate inhibitor. J. Mol. Biol. 319, 173–181. 10.1016/S0022-2836(02)00262-0 12051944

[B76] Samdani A. Vetrivel U. (2018). POAP: a GNU parallel based multithreaded pipeline of open babel and AutoDock suite for boosted high throughput virtual screening. Comput. Biol. Chem. 74, 39–48. 10.1016/j.compbiolchem.2018.02.012 29533817

[B77] Schwede T. Kopp J. Guex N. Peitsch M. C. (2003). SWISS-MODEL: an automated protein homology-modeling server. Nucleic Acids Res. 31, 3381–3385. 10.1093/nar/gkg520 12824332 PMC168927

[B78] Sedlacek J. Martins L. M. Danek P. Pombeiro A. J. Cvek B. (2014). Diethyldithiocarbamate complexes with metals used as food supplements show different effects in cancer cells. J. Appl. Biomed. 12 (4), 301–308. 10.1016/j.jab.2014.04.002

[B79] Shannon P. Markiel A. Ozier O. Baliga N. S. Wang J. T. Ramage D. (2003). Cytoscape: a software environment for integrated models of biomolecular interaction networks. Genome Res. 13, 2498–2504. 10.1101/gr.1239303 14597658 PMC403769

[B80] Shen X. Sheng H. Zhang Y. Dong X. Kou L. Yao Q. (2024). Nanomedicine-based disulfiram and metal ion co-delivery strategies for cancer treatment. Int. J. PHARM- X, 100248. 10.1016/j.ijpx.2024.100248 PMC11059435 38689600

[B81] Shivankar B. R. Bhandare V. V. Joshi K. Patil V. S. Dhotare P. S. Sonawane K. D. (2024). Investigation of cathinone analogs targeting human dopamine transporter using molecular modeling. J. Biomol. Struct. Dyn. 18, 1–16. 10.1080/07391102.2024.2335303 38698732

[B82] Sica D. A. (2005). Angiotensin-converting enzyme inhibitors' side effects—physiologic and non-physiologic considerations. J. Clin. Hypertens. 7, 17–23. 10.1111/j.1524-6175.2005.04556.x PMC8109542 15249800

[B83] Singh B. M. Mathew M. (2022). Epithelial-mesenchymal transition and its role in renal fibrogenesis. Braz Arch. Biol. Technol. 65, e22210260. 10.1590/1678-4324-2022210260

[B84] Stelzer G. Rosen N. Plaschkes S. Zimmerman S. Twik M. Fishilevich S. (2016). The GeneCards suite: from gene data mining to disease genome sequence analyses. Curr. Protoc. Bioinform. 54, 1.30.1–1.30.33. 10.1002/cpbi.5 27322403

[B85] Tian W. Chen C. Lei X. Zhao J. Liang J. (2018). CASTp 3.0: computed atlas of surface topography of proteins. Nucleic Acids Res. 46, W363-W367–W367. 10.1093/nar/gky473 29860391 PMC6031066

[B86] Trott O. Olson A. J. (2010). AutoDock Vina: improving the speed and accuracy of docking with a new scoring function, efficient optimization, and multithreading. J. Comput. Chem. 31, 455–461. 10.1002/jcc.21334 19499576 PMC3041641

[B87] Wang J. Hu K. Cai X. Yang B. He Q. Wang J. (2022). Targeting PI3K/AKT signaling for treatment of idiopathic pulmonary fibrosis. Acta Pharm. Sin. B 12, 18–32. 10.1016/j.apsb.2021.07.023 35127370 PMC8799876

[B88] Wang J. Wang W. Kollman P. A. Case D. A. (2001). Antechamber: an accessory software package for molecular mechanical calculations. J. Am. Chem. Soc. 222, 2001.

[B89] Wei X. Wang Y. Lao Y. Weng J. Deng R. Li S. (2023). Effects of honokiol protects against chronic kidney disease via BNIP3/NIX and FUNDC1-mediated mitophagy and AMPK pathways. Mol. Biol. Rep. 50 (8), 6557–6568. 10.1007/s11033-023-08592-1 37338733

[B90] Weiser D. Drozdkova D. Smesny K. Trtkova K. (2021). Possible therapeutic potential of disulfiram for multiple myeloma. Curr. Oncol. 28, 2087–2096. 10.3390/curroncol28030193 34205025 PMC8293232

[B91] Xu Z. Luo W. Chen L. Zhuang Z. Yang D. Qian J. (2022). Ang II (angiotensin II)–Induced FGFR1 (fibroblast growth factor receptor 1) activation in tubular epithelial cells promotes hypertensive kidney fibrosis and injury. Hypertension 79 (9), 2028–2041. 10.1161/HYPERTENSIONAHA.122.18657 35862110

[B92] Yang H. Rudge D. G. Koos B. Vaidialingam B. Yang H. J. Pavletich N. P. (2013). mTOR kinase structure, mechanism and regulation. Nature 497, 217–223. 10.1038/nature12122 23636326 PMC4512754

[B93] Yoshiyasu N. Matsuki R. Sato M. Urushiyama H. Toda E. Terasaki Y. (2024). Disulfiram, an anti-alcoholic drug, targets macrophages and attenuates acute rejection in rat lung allografts. Transpl. Int. 2024, 12556–12612. 10.3389/ti.2024.12556 PMC11033352 38650846

[B94] You R. Zhou W. Li Y. Zhang Y. Huang S. Jia Z. (2020). Inhibition of ROCK2 alleviates renal fibrosis and the metabolic disorders in the proximal tubular epithelial cells. Clin. Sci. 134 (12), 1357–1376. 10.1042/CS20200030 32490513

[B95] Zaldívar-Machorro V. J. López-Ortiz M. Demare P. Regla I. Muñoz-Clares R. A. (2011). The disulfiram metabolites S-methyl-N, N-diethyldithiocarbamoyl sulfoxide and S-methyl-N, N-diethylthiocarbamoyl sulfone irreversibly inactivate betaine aldehyde dehydrogenase from *Pseudomonas aeruginosa*, both *in vitro* and *in situ*, and arrest bacterial growth. Biochimie 93 (2), 286–295. 10.1016/j.biochi.2010.09.022 20933050

[B96] Zeisberg E. M. Potenta S. E. Sugimoto H. Zeisberg M. Kalluri R. (2008). Fibroblasts in kidney fibrosis emerge via endothelial-to-mesenchymal transition. J. Am. Soc. Nephrol. 19 (12), 2282–2287. 10.1681/ASN.2008050513 18987304 PMC2588112

[B97] Zha J. Chen F. Dong H. Shi P. Yao Y. Zhang Y. (2014). Disulfiram targeting lymphoid malignant cell lines via ROS-JNK activation as well as Nrf2 and NF-kB pathway inhibition. J. Transl. Med. 12, 163–169. 10.1186/1479-5876-12-163 24915933 PMC4075939

[B98] Zhang H. Chen D. Ringler J. Chen W. Cui Q. C. Ethier S. P. (2010). Disulfiram treatment facilitates phosphoinositide 3-kinase inhibition in human breast cancer cells *in vitro* and *in vivo* . Cancer Res. 70, 3996–4004. 10.1158/0008-5472.CAN-09-3752 20424113 PMC3827685

[B99] Zhang Y. Zhang R. Han X. (2021). Disulfiram inhibits inflammation and fibrosis in a rat unilateral ureteral obstruction model by inhibiting gasdermin D cleavage and pyroptosis. Inflamm. Res. 70, 543–552. 10.1007/s00011-021-01457-y 33851234

[B100] Zheng H. Yang Z. Xin Z. Yang Y. Yu Y. Cui J. (2020). Glycogen synthase kinase-3β: a promising candidate in the fight against fibrosis. Theranostics 10, 11737–11753. 10.7150/thno.47717 33052244 PMC7545984

[B101] Zhou D. Tan R. J. Liu Y. (2016). Sonic hedgehog signaling in kidney fibrosis: a master communicator. Sci. China Life Sci. 59, 920–929. 10.1007/s11427-016-0020-y 27333788 PMC5540157

[B102] Zuo Y. Chun B. Potthoff S. A. Kazi N. Brolin T. J. Orhan D. (2013). Thymosin β4 and its degradation product, Ac-SDKP, are novel reparative factors in renal fibrosis. Kidney Int. 84 (6), 1166–1175. 10.1038/ki.2013.209 23739235 PMC3830708

