# Supplementary material for: Exploring disulfiram mechanisms in renal fibrosis: insights from biological data and computational approaches
Source: Front Pharmacol. 2025 Mar 18;16:1480732. doi: 10.3389/fphar.2025.1480732 (PMC11958968; doi:10.3389/fphar.2025.1480732)

```
4JPS            PPRPSSGELWGIHLMPPRILVECLLPNGMIVTLECLREATLITIKHELFKEARKYPLHQL
4JPS_model      PPRPSSGELWGIHLMPPRILVECLLPNGMIVTLECLREATLITIKHELFKEARKYPLHQL
                ************************************************************

4JPS            LQDESSYIFVSVTQEAEREEFFDETRRLCDLRLFQPFLKVIEPVGNREEKILNREIGFAI
4JPS_model      LQDESSYIFVSVTQEAEREEFFDETRRLCDLRLFQPFLKVIEPVGNREEKILNREIGFAI
                ************************************************************

4JPS            GMPVCEFDMVKDPEVQDFRRNILNVCKEAVDLRDLNSPHSRAMYVYPPNVESSPELPKHI
4JPS_model      GMPVCEFDMVKDPEVQDFRRNILNVCKEAVDLRDLNSPHSRAMYVYPPNVESSPELPKHI
                ************************************************************

4JPS            YNKLDKGQIIVVIWVIVSPNNDKQKYTLKINHDCVPEQVIAEAIR---------------
4JPS_model      YNKLDKGQIIVVIWVIVSPNNDKQKYTLKINHDCVPEQVIAEAIRKKTRSMLLSSEQLKL
                *********************************************

4JPS            -KLEYQGKYILKVCGCDEYFLEKYPLSQYKYIRSCIMLGRMPNLMLMAKESLYSQLPMDC
4JPS_model      CVLEYQGKYILKVCGCDEYFLEKYPLSQYKYIRSCIMLGRMPNLMLMAKESLYSQLPMDC
                  **********************************************************

4JPS            FTMPSYSRRIS----------TTKSLWVINSALRIKILCATYVNVNIRDIDKIYVRTGIY
4JPS_model      FTMPSYSRRISTATPYMNGETSTKSLWVINSALRIKILCATYVNVNIRDIDKIYVRTGIY
                ***********          :**************************************

4JPS            HGGEPLCDNVNTQRVPCSNPRWNEWLNYDIYIPDLPRAARLCLSICSVKGRKGAKEEHCP
4JPS_model      HGGEPLCDNVNTQRVPCSNPRWNEWLNYDIYIPDLPRAARLCLSICSVKGRKGAKEEHCP
                ************************************************************

4JPS            LAWGNINLFDYTDTLVSGKMALNLWPVPHGLEDLLNPIGVTGSNPNKETPCLELEFDWFS
4JPS_model      LAWGNINLFDYTDTLVSGKMALNLWPVPHGLEDLLNPIGVTGSNPNKETPCLELEFDWFS
                ************************************************************

4JPS            SVVKFPDMSVIEEHANE---------------------------NDKEQLKAISTRDPLS
4JPS_model      SVVKFPDMSVIEEHANWSVSREAGFSYSHAGLSNRLARDNELRENDKEQLKAISTRDPLS
                ****************                            ****************

4JPS            EITEQEKDFLWSHRHYCVTIPEILPKLLLSVKWNSRDEVAQMYCLVKDWPPIKPEQAMEL
4JPS_model      EITEQEKDFLWSHRHYCVTIPEILPKLLLSVKWNSRDEVAQMYCLVKDWPPIKPEQAMEL
                ************************************************************

4JPS            LDCNYPDPMVRGFAVRCLEKYLTDDKLSQYLIQLVQVLKYEQYLDNLLVRFLLKKALTNQ
4JPS_model      LDCNYPDPMVRGFAVRCLEKYLTDDKLSQYLIQLVQVLKYEQYLDNLLVRFLLKKALTNQ
                ************************************************************

4JPS            RIGHFFFWHLKSEMHNKTVSQRFGLLLESYCRACGMYLKHLNRQVEAMEKLINLTDILKQ
4JPS_model      RIGHFFFWHLKSEMHNKTVSQRFGLLLESYCRACGMYLKHLNRQVEAMEKLINLTDILKQ
                ************************************************************

4JPS            EKKDETQKVQMKFLVEQMRRPDFMDALQGFLSPLNPAHQLGNLRLEECRIMSSAKRPLWL
4JPS_model      EKKDETQKVQMKFLVEQMRRPDFMDALQGFLSPLNPAHQLGNLRLEECRIMSSAKRPLWL
                ************************************************************

4JPS            NWENPDIMSELLFQNNEIIFKNGDDLRQDMLTLQIIRIMENIWQNQGLDLRMLPYGCLSI
4JPS_model      NWENPDIMSELLFQNNEIIFKNGDDLRQDMLTLQIIRIMENIWQNQGLDLRMLPYGCLSI
                ************************************************************

4JPS            GDCVGLIEVVRNSHTIMQIQCK--------FNSHTLHQWLKDKNKGEIYDAAIDLFTRSC
4JPS_model      GDCVGLIEVVRNSHTIMQIQCKGGLKGALQFNSHTLHQWLKDKNKGEIYDAAIDLFTRSC
                **********************        ******************************

4JPS            AGYCVATFILGIGDRHNSNIMVKDDGQLFHIDFGHFLDHKKKKFGYKRERVPFVLTQDFL
4JPS_model      AGYCVATFILGIGDRHNSNIMVKDDGQLFHIDFGHFLDHKKKKFGYKRERVPFVLTQDFL
                ************************************************************

4JPS            IVISKGAQECTKTREFERFQEMCYKAYLAIRQHANLFINLFSMMLGSGMPELQSFDDIAY
4JPS_model      IVISKGAQECTKTREFERFQEMCYKAYLAIRQHANLFINLFSMMLGSGMPELQSFDDIAY
                ************************************************************

4JPS            IRKTLALDKTEQEALEYFMKQMNDAHHGGWTTKMDWIFHTEWYWGDISREEVNEKLRDTA
4JPS_model      IRKTLALDKTEQEALEYFMKQMNDAHHGGWTTKMDWIFHTEWYWGDISREEVNEKLRDTA
                ************************************************************

4JPS            DGTFLVRDASTKDY---TLTLRKGGNNKLIKIFHR----GFSDPLTFKLLYPVS------
4JPS_model      DGTFLVRDASTKMHGDYTLTLRKGGNNKLIKIFHRDGKYGFSDPLTFSSVVELINHYRNE
                ************ :   ******************    ********. :  :

4JPS            ---------------------KDQVVKEDNIEAVGKKLHEYNTQFQEKSREYDRLYEEYT
4JPS_model      SLAQYNPKLDVKLLYPVSKYQQDQVVKEDNIEAVGKKLHEYNTQFQEKSREYDRLYEEYT
                                     :**************************************

4JPS            RTSQEIQMKRTAIEAFNETIKIFEEQCQTQERYSKEYIEKFKREGNEKEIQRIMHNYDKL
4JPS_model      RTSQEIQMKRTAIEAFNETIKIFEEQCQTQERYSKEYIEKFKREGNEKEIQRIMHNYDKL
                ************************************************************

4JPS            KSRISEIIDSRRRLEEDLKKQAAEYREIDKRMNSIKPDLIQLRKTRDQYLMWLTQKGVRQ
4JPS_model      KSRISEIIDSRRRLEEDLKKQAAEYREIDKRMNSIKPDLIQLRKTRDQYLMWLTQKGVRQ
                ************************************************************

4JPS            K
4JPS_model      K
                *
```

Supplementary Figure 1a: CLUSTAL 2.1 Multiple Sequence Alignments of PDB ID: 4JPS (PIK3CA) with its model structure from the SwissModel.

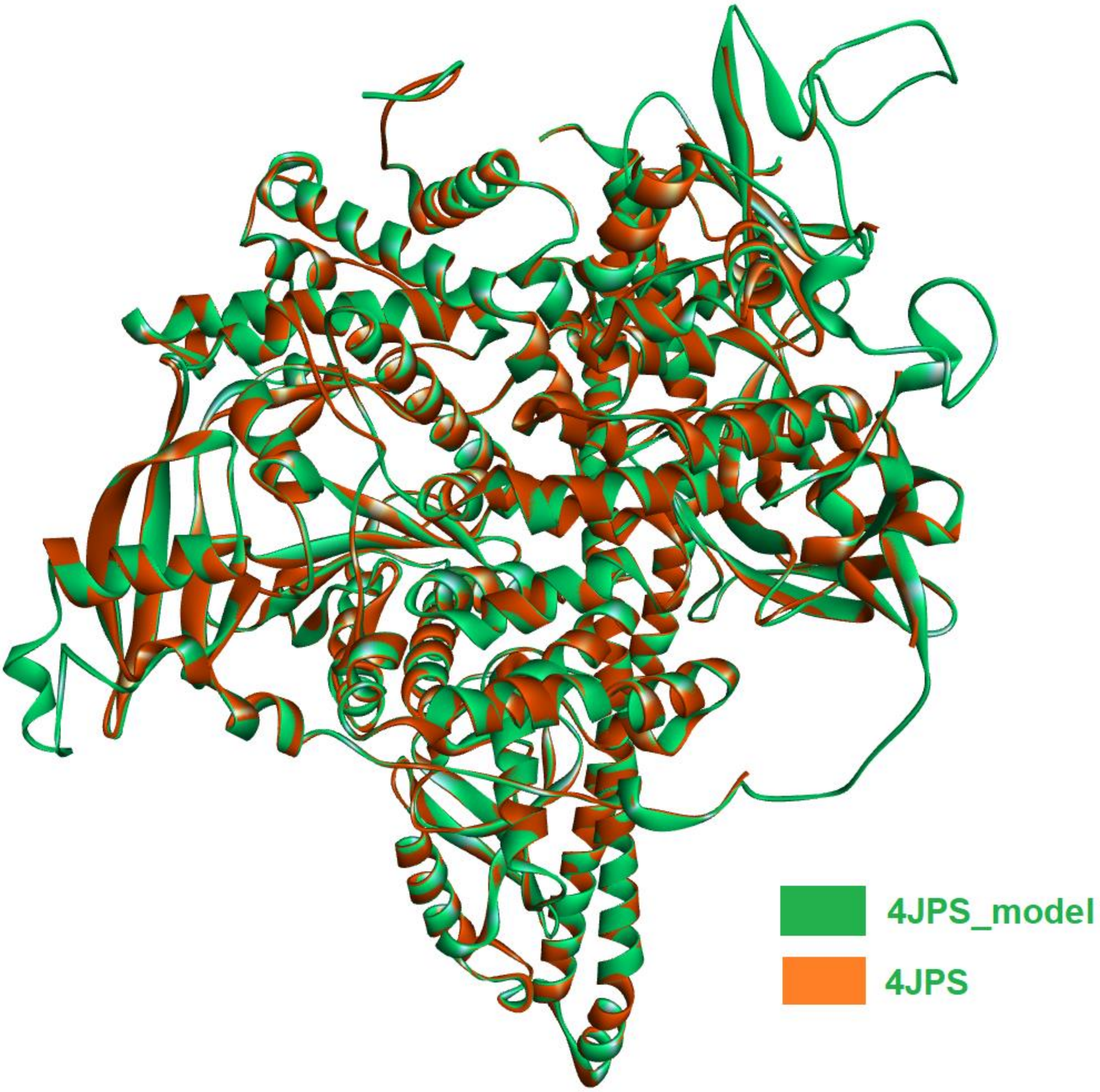

Supplementary Figure 1b: Alignment (overlay) of 4JPS (orange) obtained from the RCSB PDB with model (green) obtained from the SwissModel.

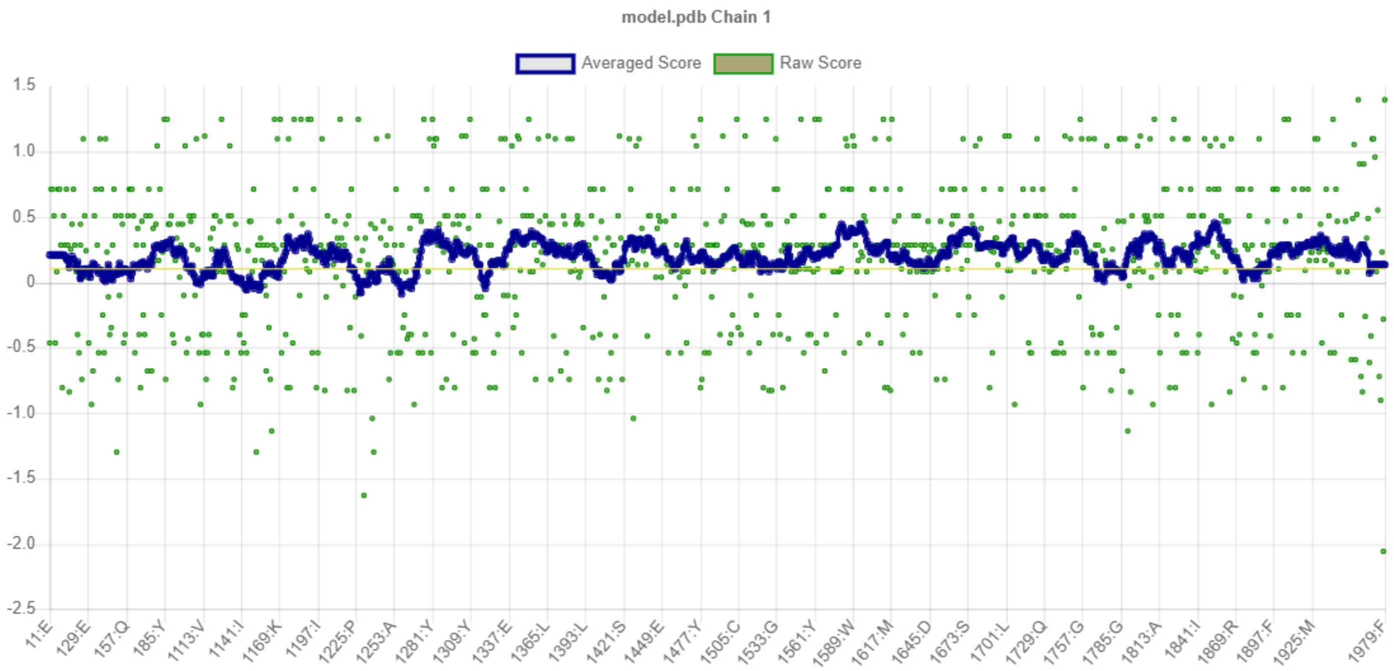

VERIFY3D

82.33% of the residues have averaged 3D-1D score >= 0.1

Pass

At least 80% of the amino acids have scored >= 0.1 in the 3D/1D profile.

Supplementary Figure 1c: Validation of 4JPS_model using Verify3D.

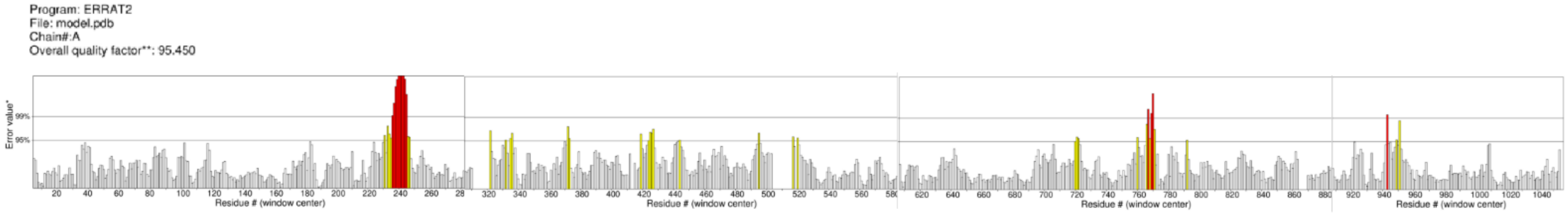

Supplementary Figure 1d: Validation of 4JPS_model using ERRAT2 showing 95.45% of quality factor.

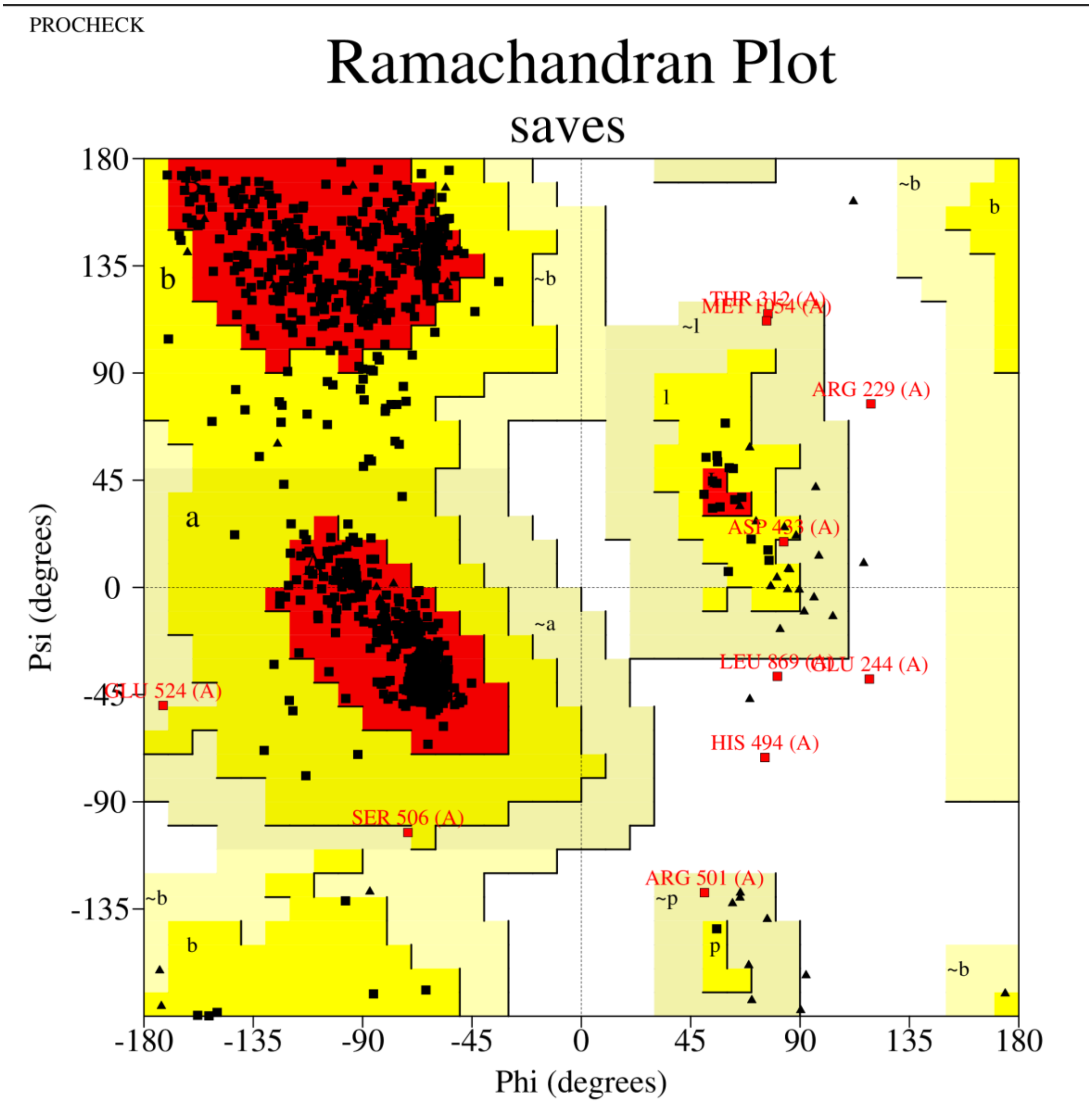

Plot statistics

Residues in most favoured regions [A,B,L]	873	91.0%
Residues in additional allowed regions [a,b,l,p]	76	7.9%
Residues in generously allowed regions [~a,~b,~l,~p]	6	0.6%
Residues in disallowed regions	4	0.4%
	----	------
Number of non-glycine and non-proline residues	959	100.0%
Number of end-residues (excl. Gly and Pro)	3	
Number of glycine residues (shown as triangles)	49	
Number of proline residues	51	

Total number of residues	1062	

Supplementary Figure 1d: Validation of 4JPS_model using Ramachandran Plot showing about 98.9% residues in most favoured and additionally allowed region.

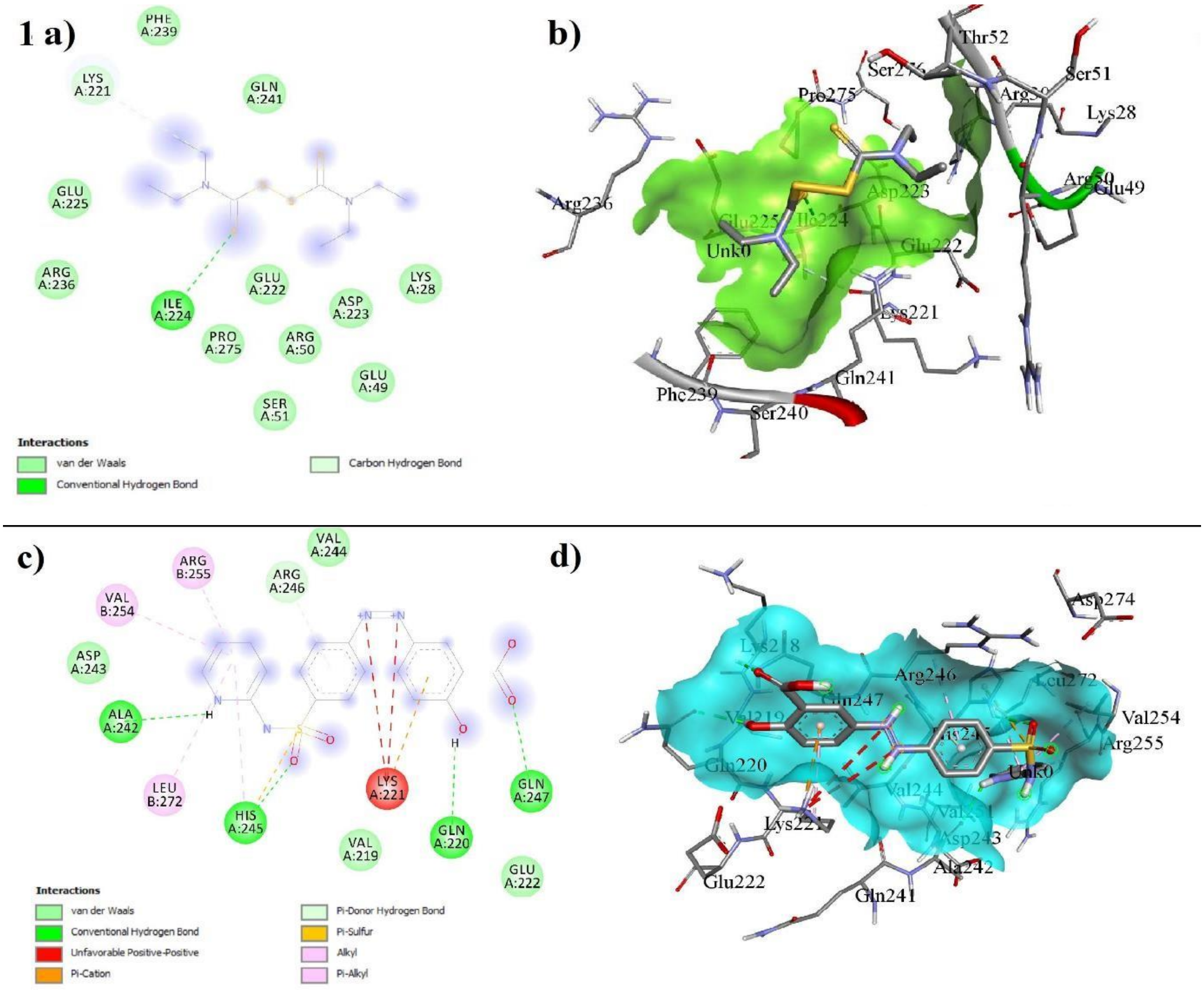

Supplementary Figure S2: 2D and 3D representation of Intermolecular interaction of DSF (a and b) and sulfasalazine (c and d) with NFKB. DSF formed one H-Bond with Ile224 and one hydrophobic bond with Lys221, whereas, sulfasalazine formed four H-Bond with Ala243, His245, Gln220, Gln247 and 6 hydrophobic bonds with Val254, Arg255, Leu272, His245 (2), Lys221.

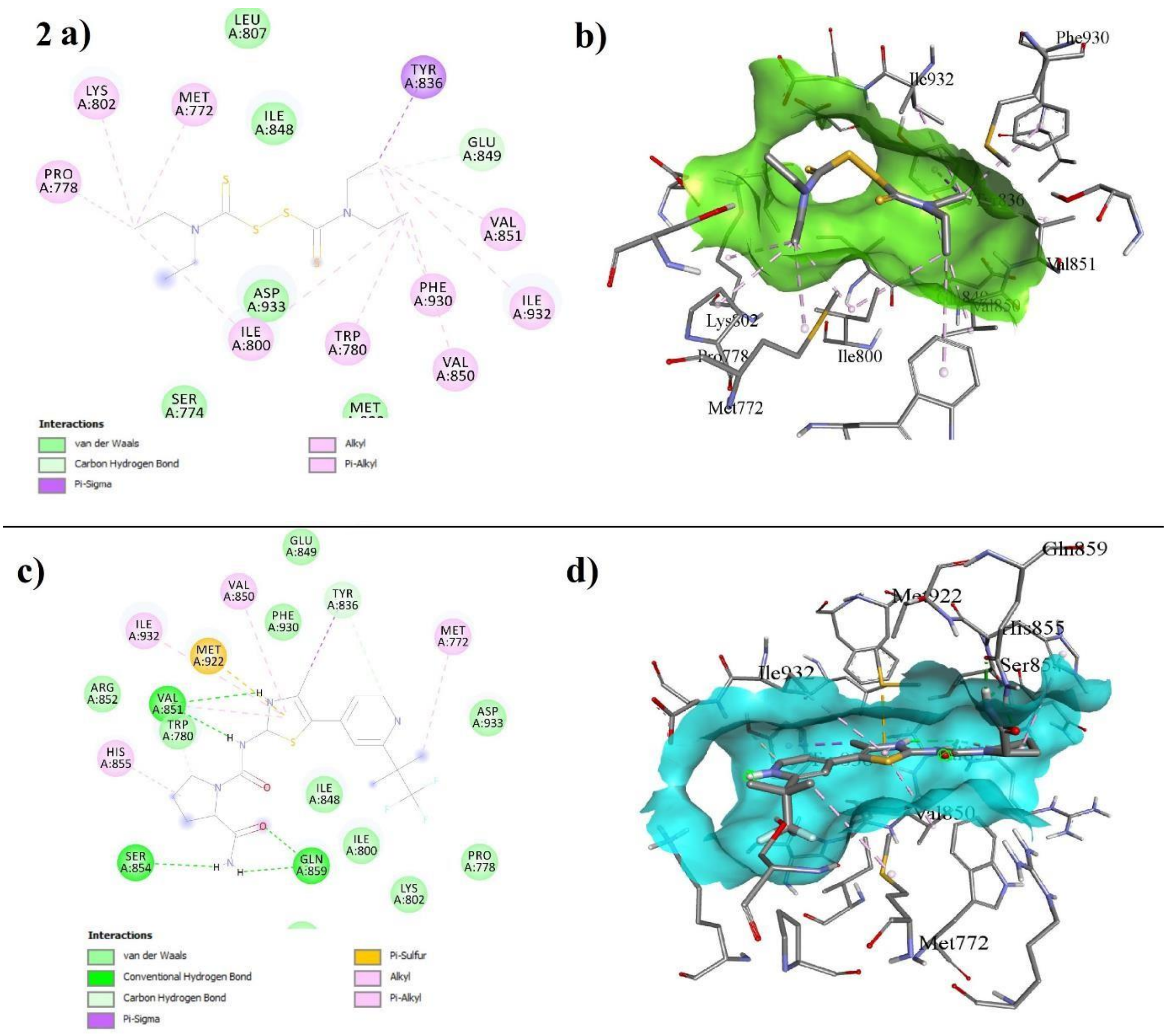

Supplementary Figure S3: 2D and 3D representation of Intermolecular interaction of DSF (a and b) and Alpelisib (c and d) with PIK3CA. DSF formed 11 hydrophobic bonds with Pro778, Lys802, Met772, Ile800, Trp780, Phe930, Val850, Ile932, Val851, Glu849, Tyr836, whereas, Alpelisib formed five H-Bond with Val851 (2), Ser854, Gln859 (2) and 11 hydrophobic bonds with His855, Val851 (4), Ile932, Met922, Val850, Tyr836 (2), Met772.

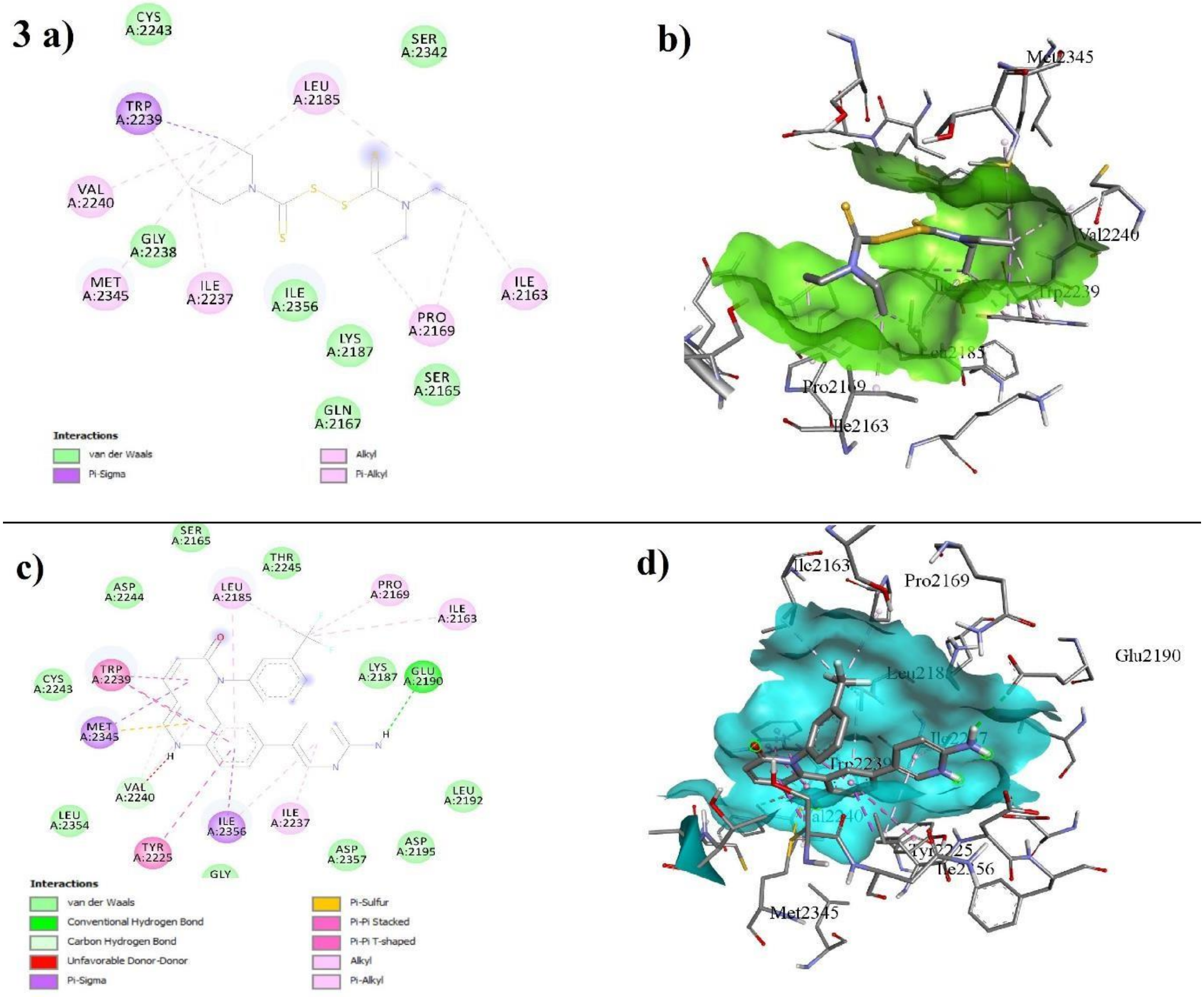

Supplementary Figure S4: 2D and 3D representation of Intermolecular interaction of DSF (a and b) and Torin- 2 (c and d) with MTOR. DSF formed 10 hydrophobic bonds with Leu2185 (2), Trp2239 (2), Val2240, Met2345, Ile2237, Pro2169 (2), Ile2163, whereas, Torin-2 formed one H-Bond with Glu2190 and 14 hydrophobic bonds with Ile2237, Ile2356 (2), Tyr2225, Val2240 (2), Met2345 (2), Trp2239 (2), Leu2185 (2), Pro2169, Ile2163.

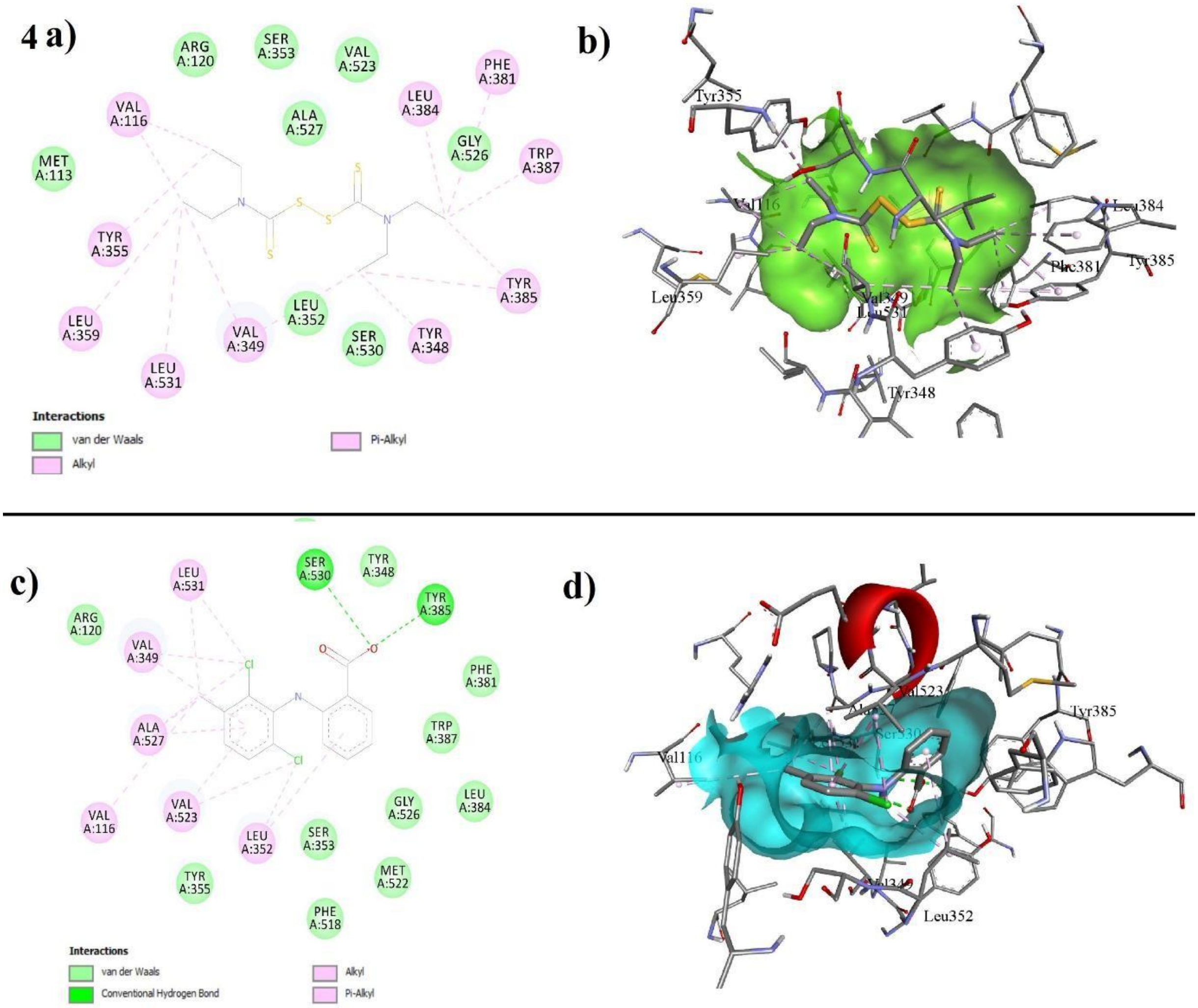

Supplementary Figure S5: 2D and 3D representation of Intermolecular interaction of DSF (a and b) and Meclofenamic acid (c and d) with PTGS2. DSF formed 13 hydrophobic bonds with Val116 (2), Tyr355, Leu359, Leu531, Val349 (2), Tyr348, Tyr385 (2), Trp387, Phe381, Leu384, whereas, meclofenamic acid formed 2 H-Bond with Ser530, Tyr385 and 11 hydrophobic bonds with Leu531 (2), Val341 (2), Ala527 (2), Val116, Val523 (2), Leu352 (2).

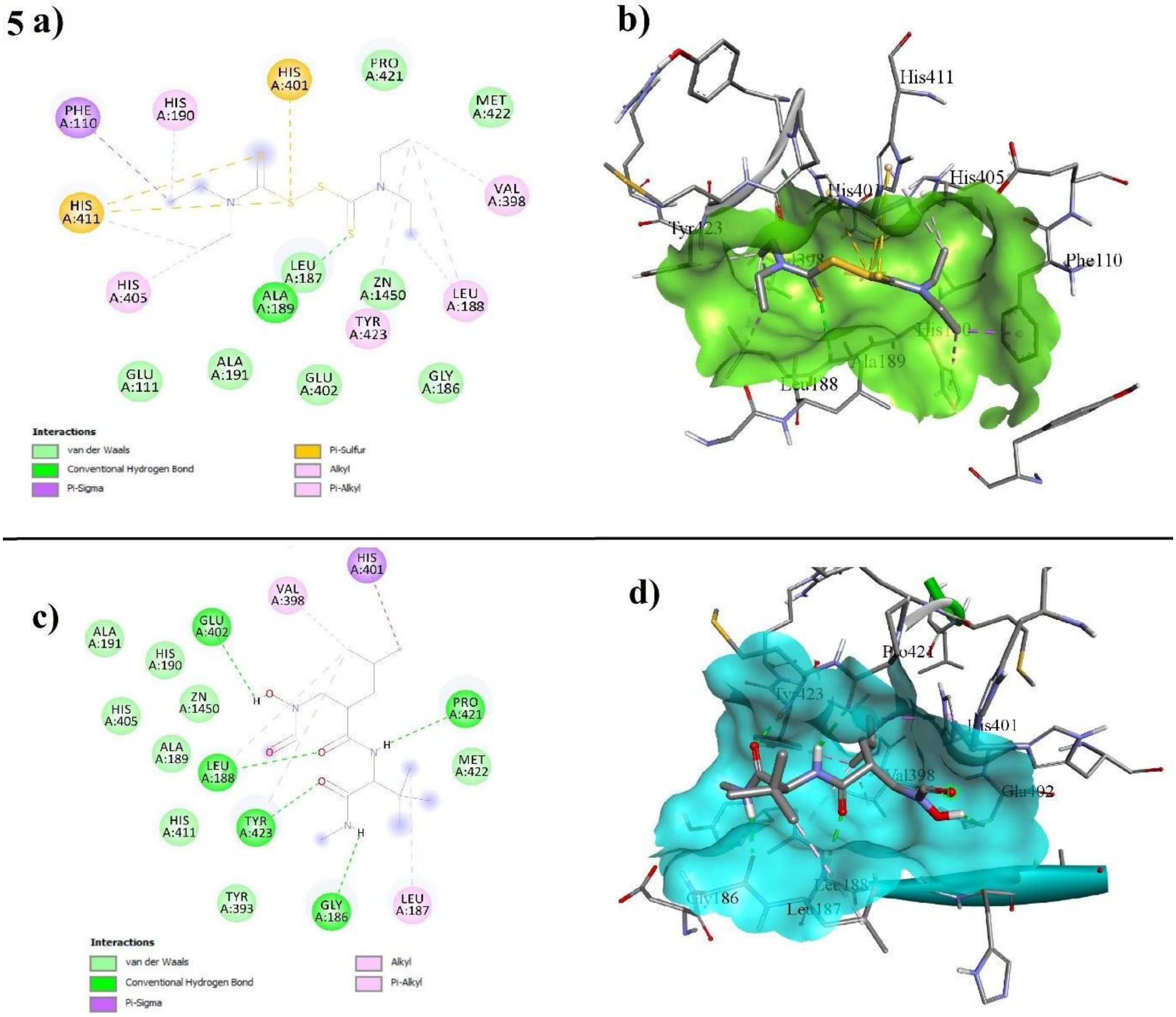

Supplementary Figure S6: 2D and 3D representation of Intermolecular interaction of DSF (a and b) and NFH (c and d) with MMP9. DSF formed 1 H-bond with Ala189 and 11 hydrophobic bonds with His411 (3), His405, Tyr423, Leu188 (2), Val398, His401, His190, Phe110, whereas, NFH formed 5 H-Bond with Glu402, Leu188, Tyr423, Gly186, Pro421 and 5 hydrophobic bonds with Leu188, Tyr423, Leu187, Val398, His401.

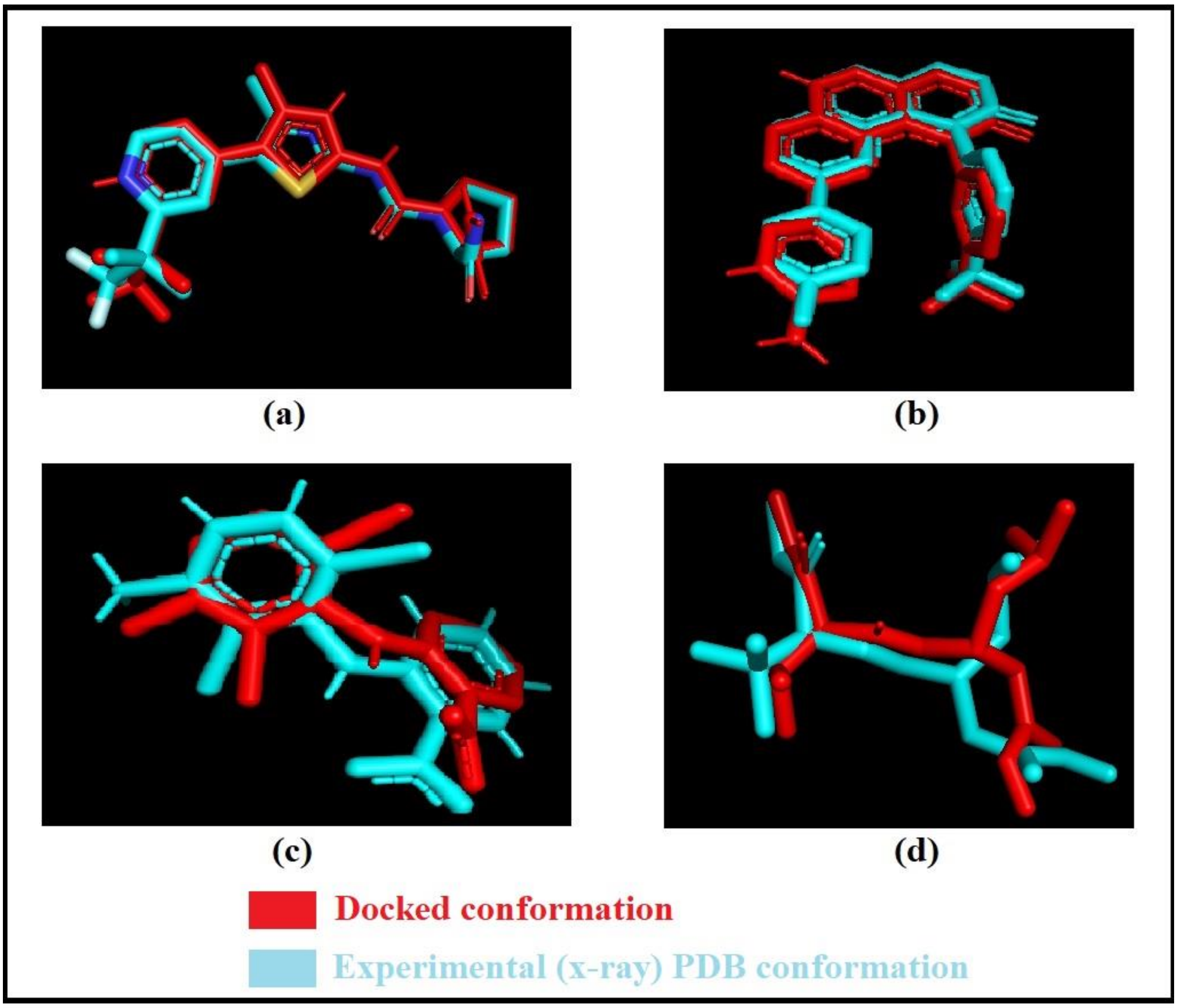

Supplementary Figure S7: Overlay of docked conformations (red) and experimental (X-ray) PDB conformations (cyan) for (a) Alpelisib (PIK3R1/PIK3CA), (b) Torin-2 (mTOR), (c) Meclofenamic acid (PTGS2), and (d) NFH (MMP9). The RMSD values between the conformations were found to be 0.498, 0.657, 0.625, and 1.336 Å, respectively, showing the accuracy of the docking protocol in predicting binding poses with close alignment to experimental structures.

.

Supplementary Figure S8: NFKB - DSF

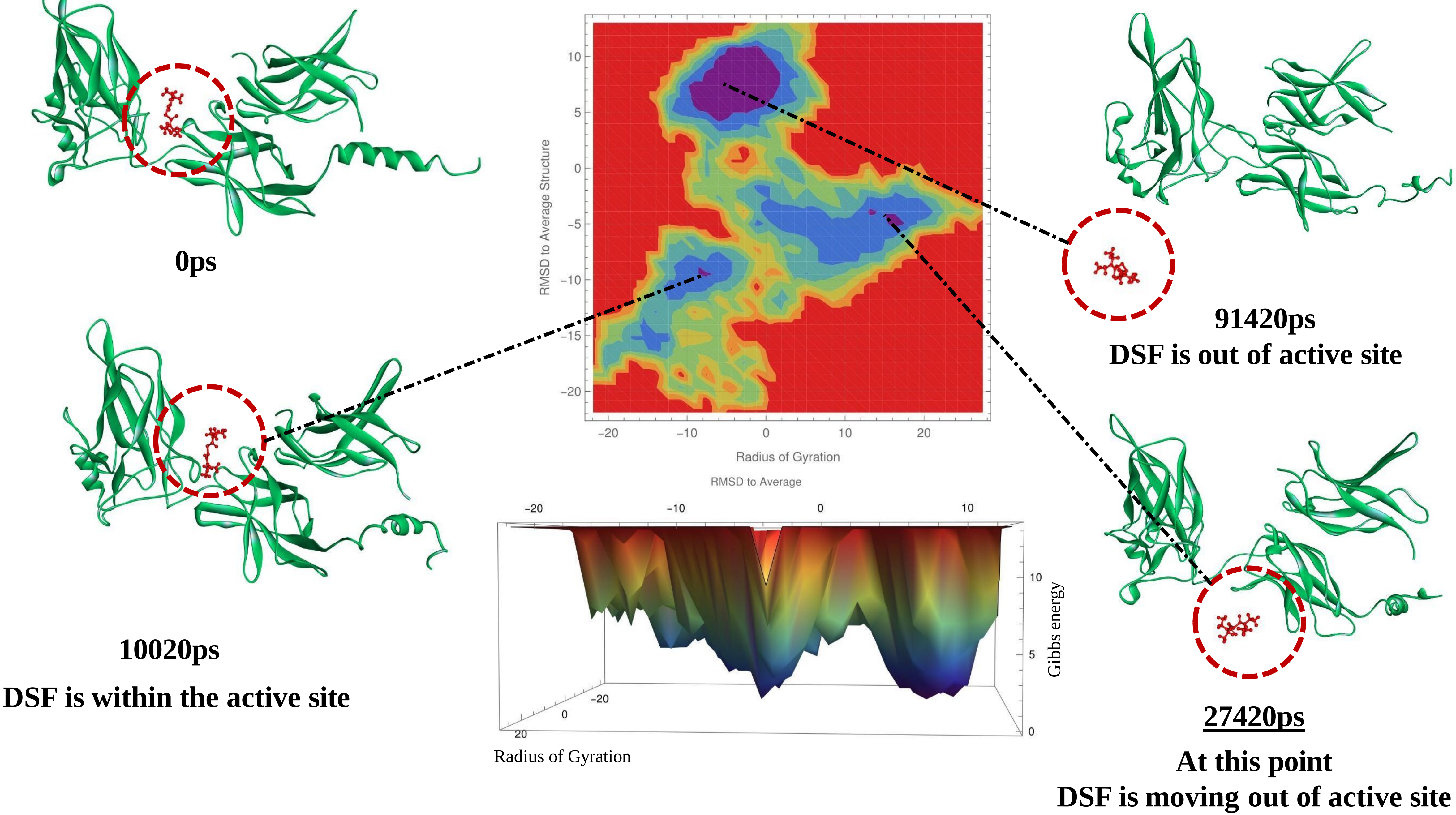

Supplementary Figure S9: NFKB - Sulfasalazine

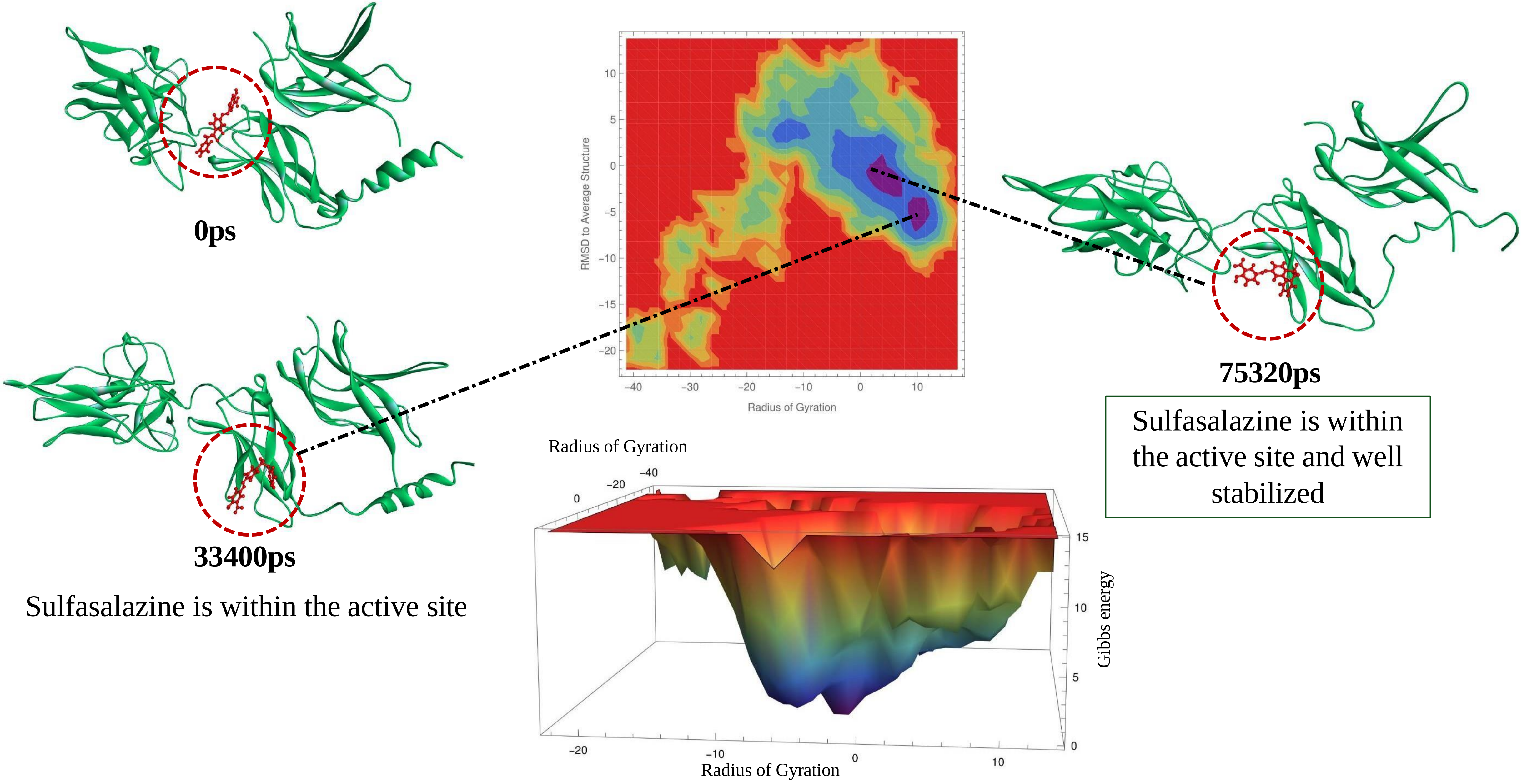

Supplementary Figure S10: PIK3R1/PIK3CA - DSF

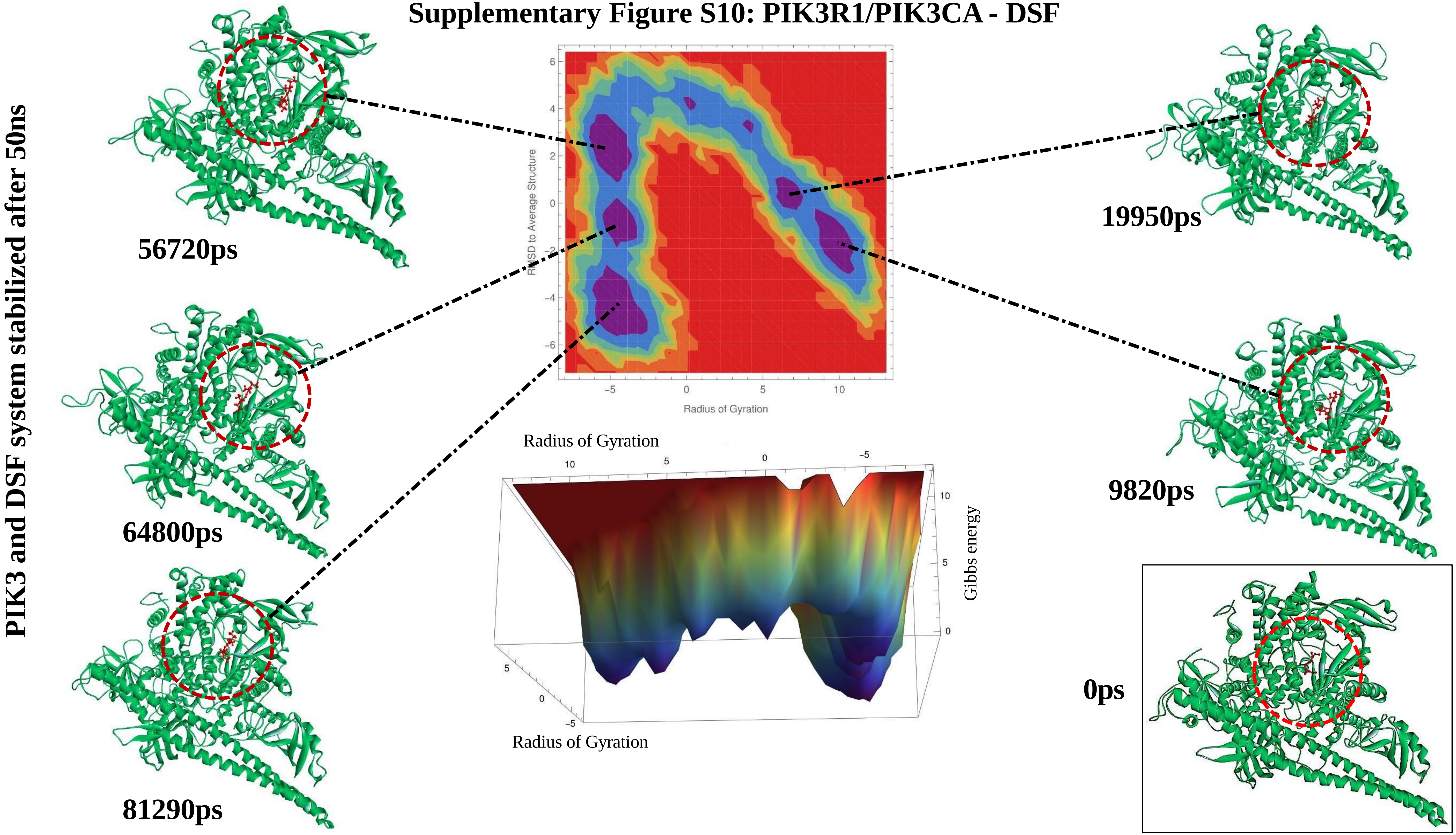

Supplementary Figure S11: PIK3R1/PIK3CA - Alpelisib

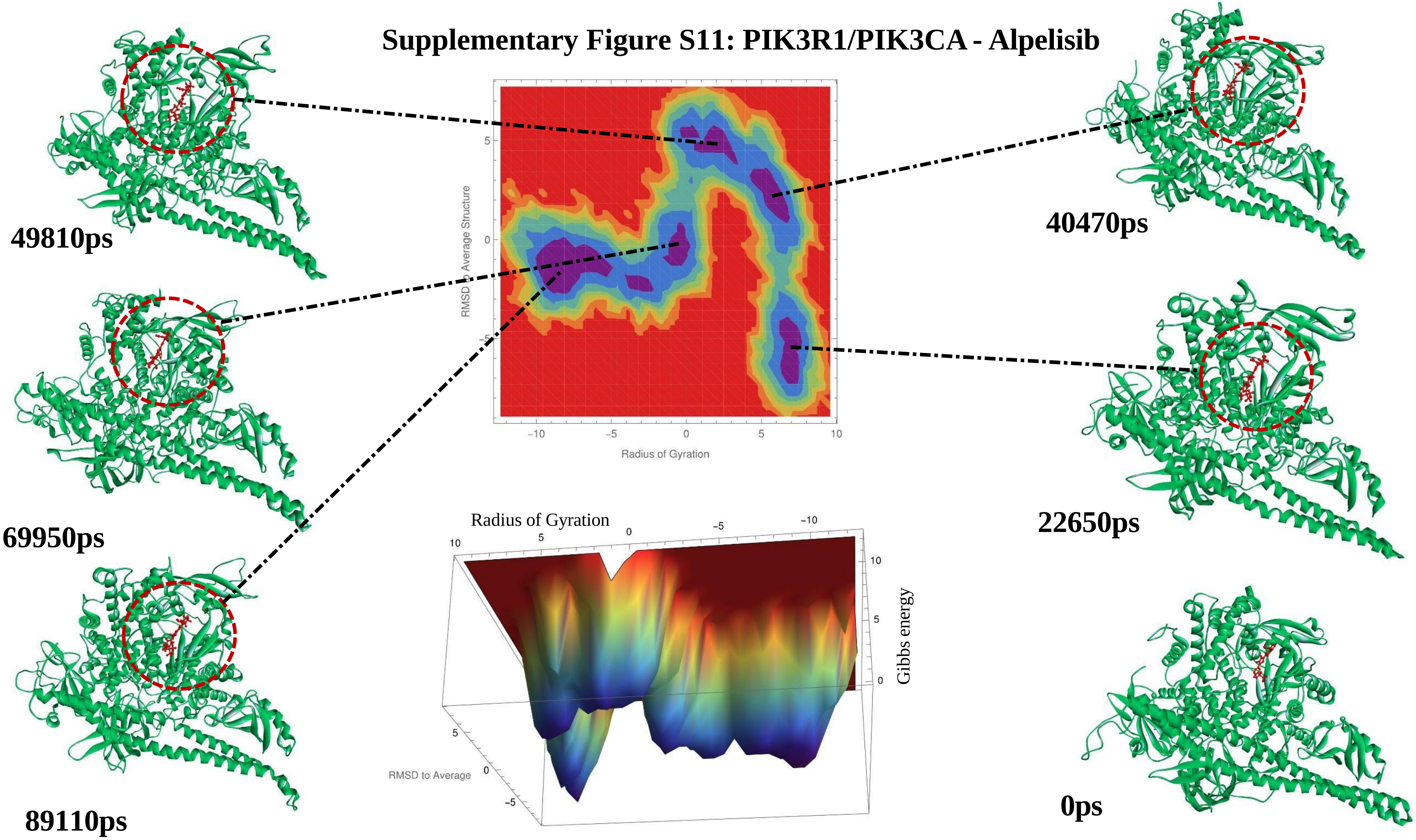

Supplementary Figure S12: MTOR - DSF

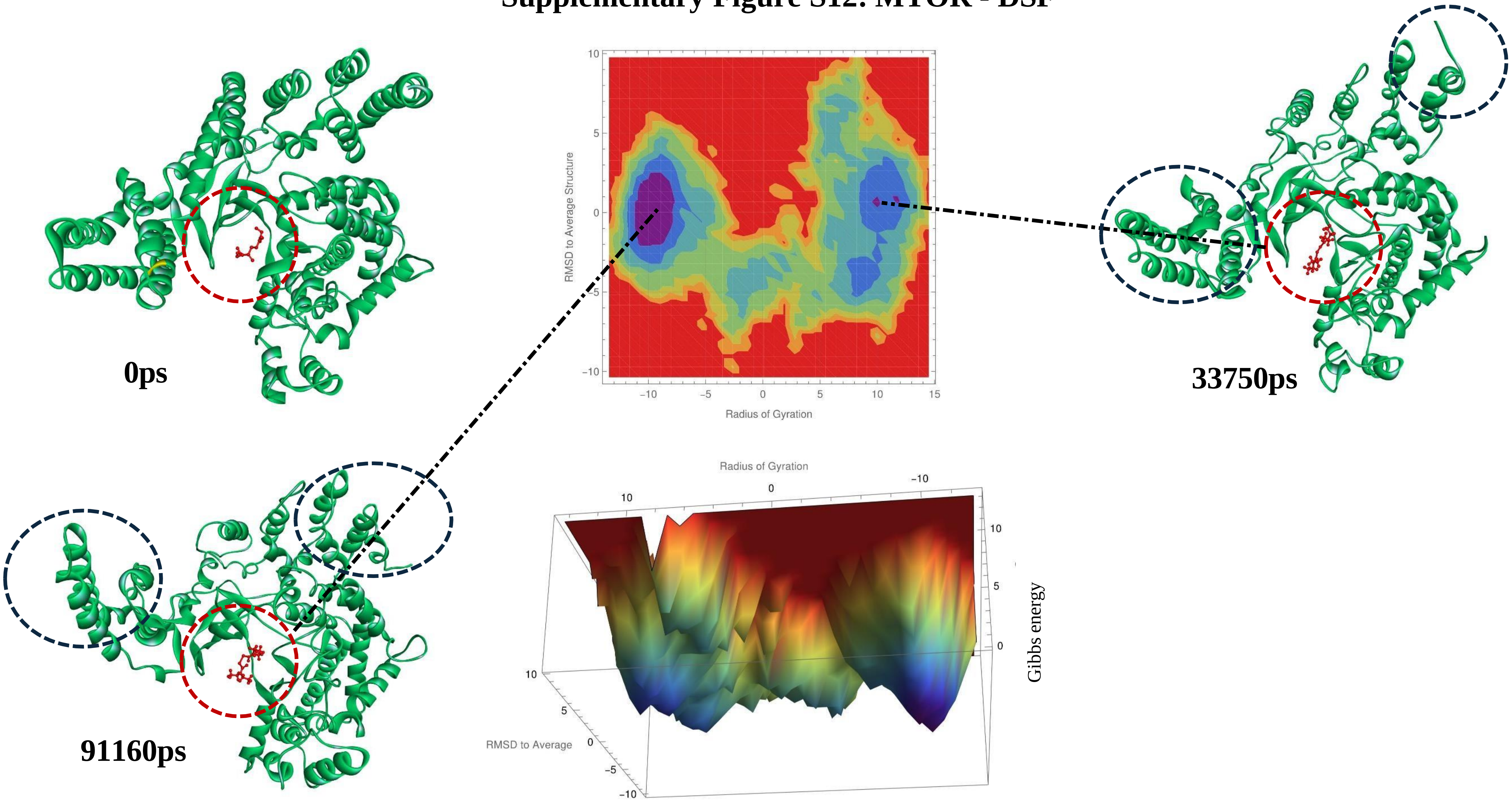

Supplementary Figure S13: MTOR - Torin-2

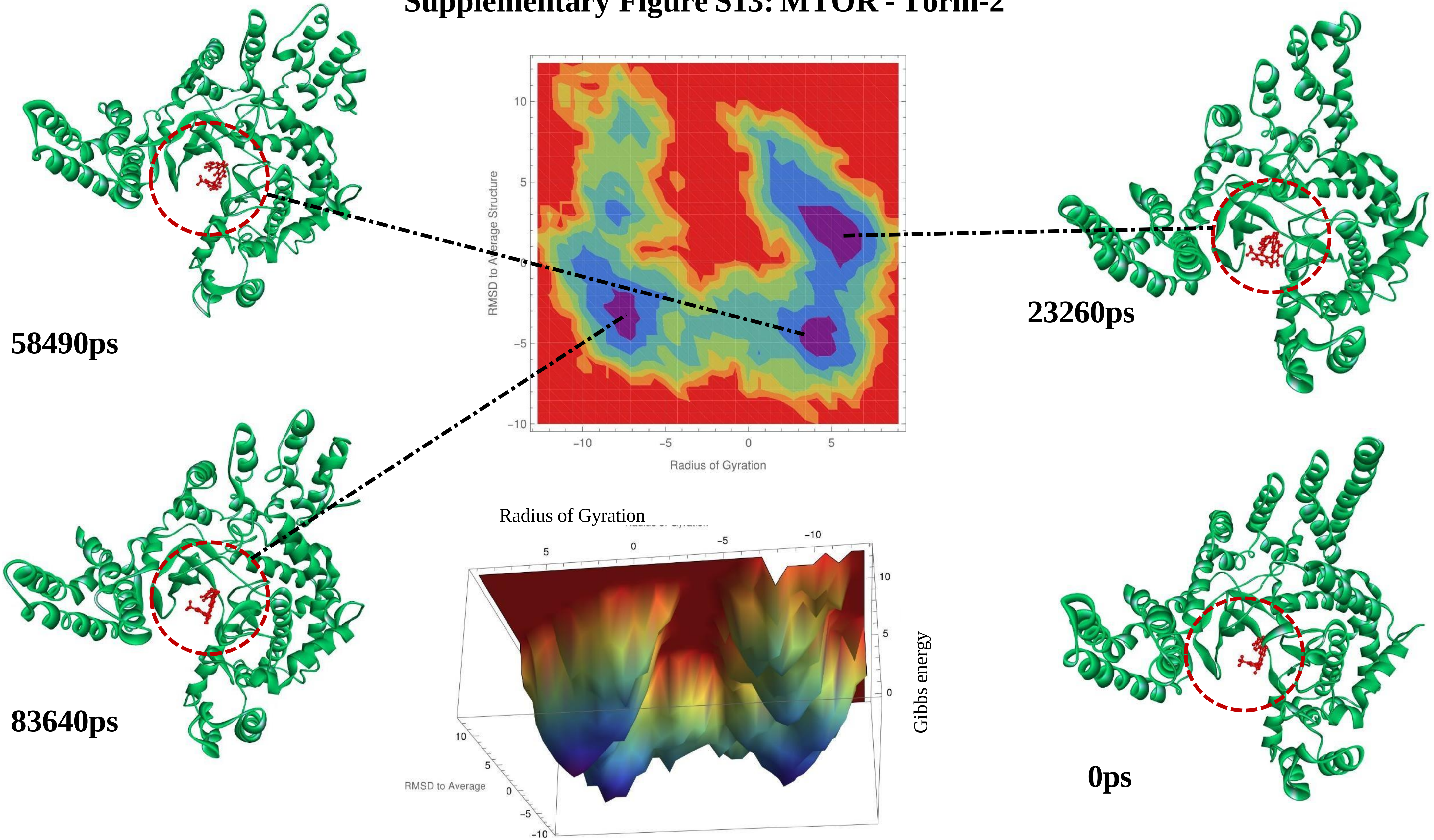

Supplementary Figure S14: PTGS2 - DSF

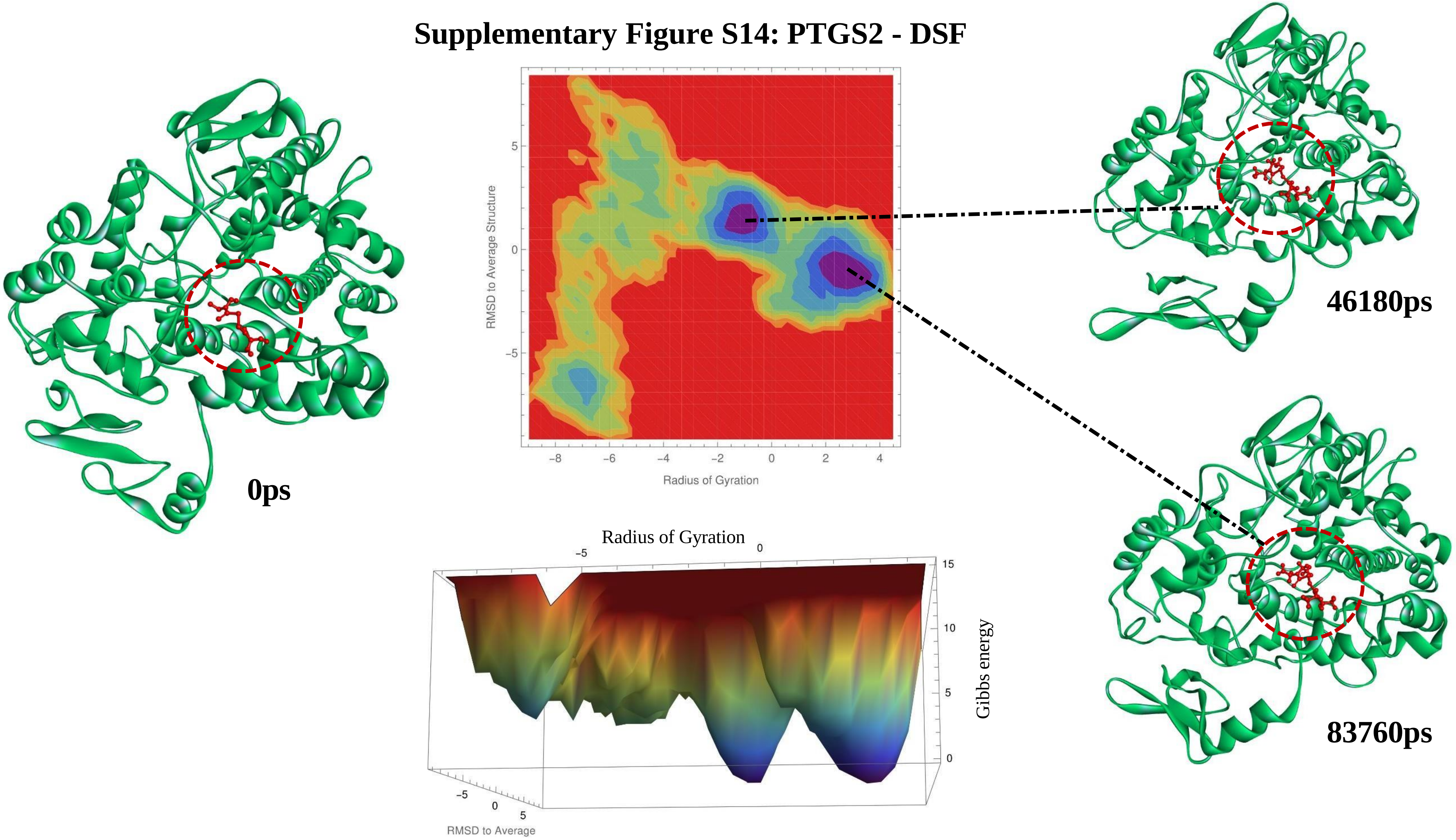

Supplementary Figure S15: PTGS2 – Meclofenamic acid

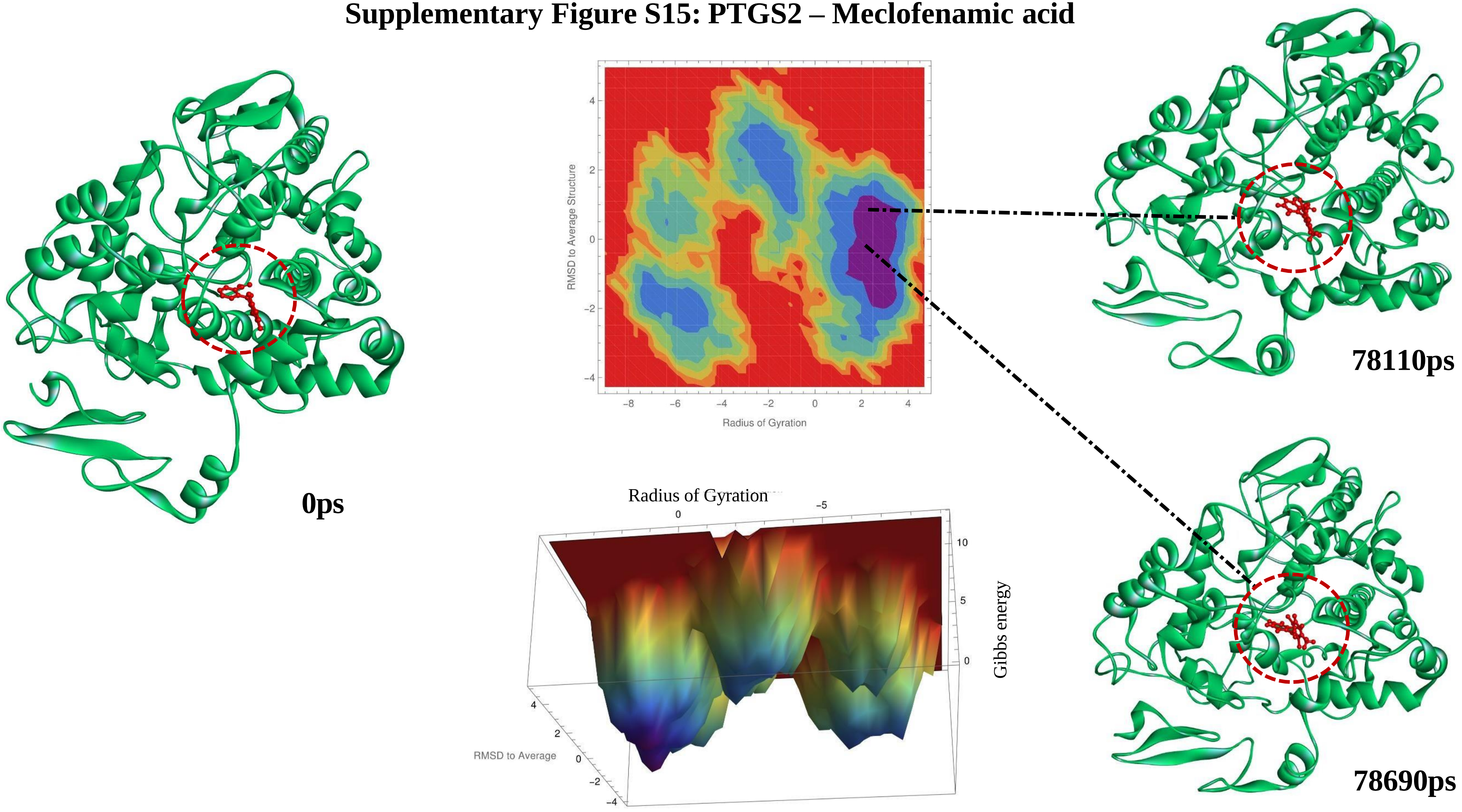

Supplementary Figure S16: MMP9 – DSF

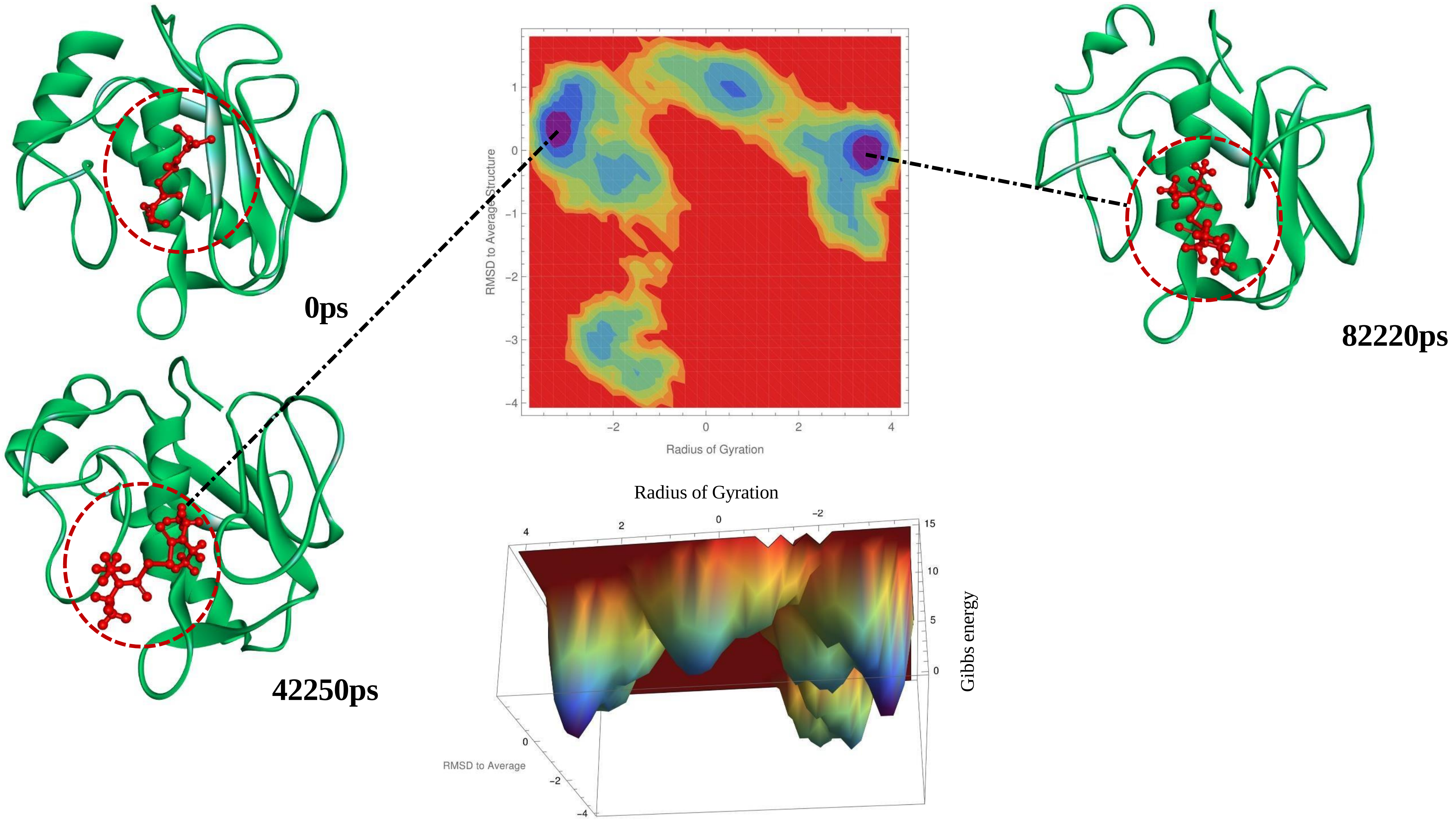

Supplementary Figure S17: MMP9 – NFH

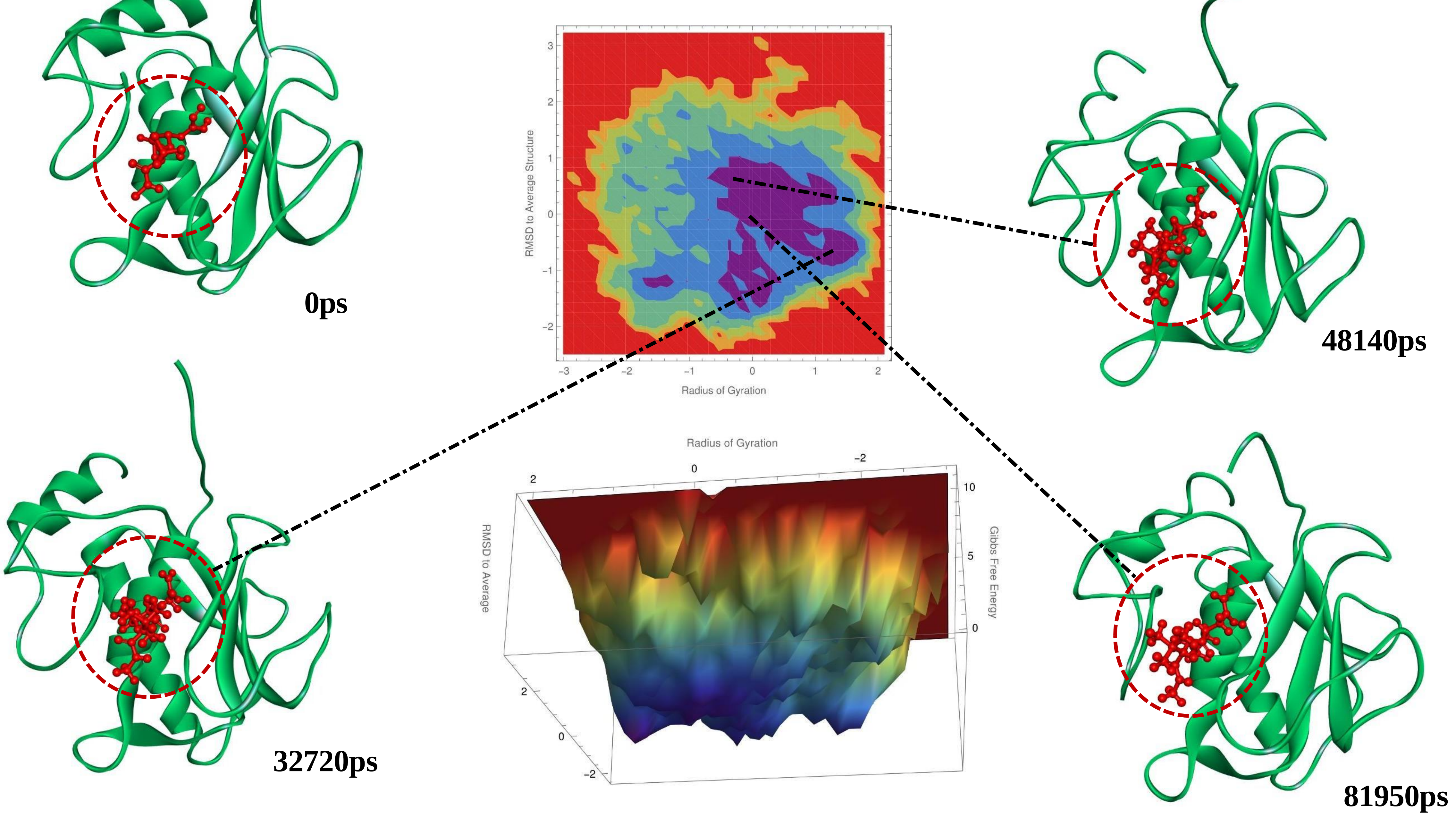

Supplement: Supplementary file 5 [file DataSheet1.pdf]
